# Prevalence of syphilis among men who have sex with men: a global systematic review and meta-analysis from 2000–20

**DOI:** 10.1016/S2214-109X(21)00221-7

**Published:** 2021-07-08

**Authors:** Motoyuki Tsuboi, Jayne Evans, Ella P Davies, Jane Rowley, Eline L Korenromp, Tim Clayton, Melanie M Taylor, David Mabey, R Matthew Chico

**Affiliations:** Department of Disease Control, Faculty of Infectious and Tropical Diseases, London School of Hygiene & Tropical Medicine, London, UK; Department of Disease Control, Faculty of Infectious and Tropical Diseases, London School of Hygiene & Tropical Medicine, London, UK; Department of Disease Control, Faculty of Infectious and Tropical Diseases, London School of Hygiene & Tropical Medicine, London, UK; London, UK; Avenir Health, Geneva, Switzerland; Department of Medical Statistics, Faculty of Epidemiology and Population Health, London School of Hygiene & Tropical Medicine, London, UK; Department of Reproductive Health and Research, World Health Organization, Geneva, Switzerland; Centers for Disease Control and Prevention, Division of STD Prevention, Atlanta, GA, USA; Department of Clinical Research, Faculty of Infectious and Tropical Diseases, London School of Hygiene & Tropical Medicine, London, UK; Department of Disease Control, Faculty of Infectious and Tropical Diseases, London School of Hygiene & Tropical Medicine, London, UK

## Abstract

**Background:**

The WHO Global Health Sector Strategy aims to reduce worldwide syphilis incidence by 90% between 2018 and 2030. If this goal is to be achieved, interventions that target high-burden groups, including men who have sex with men (MSM), will be required. However, there are no global prevalence estimates of syphilis among MSM to serve as a baseline for monitoring or modelling disease burden. We aimed to assess the global prevalence of syphilis among MSM using the available literature.

**Methods:**

In this global systematic review and meta-analysis, we searched MEDLINE, Embase, LILACS, and AIM databases, and Integrated Bio-Behavioral Surveillance (IBBS) reports between April 23, 2019, and Feb 1, 2020, to identify studies done between Jan 1, 2000, and Feb 1, 2020, with syphilis point prevalence data measured by biological assay among MSM (defined as people who were assigned as male at birth and had oral or anal sex with at least one other man in their lifetime). Studies were excluded if participants were exclusively HIV-infected MSM, injection-drug users, only seeking care for sexually transmitted infections (STIs) or genital symptoms, or routine STI clinic attendees. Data were extracted onto standardised forms and cross-checked for accuracy and validity. We used random-effects models to generate pooled prevalence estimates across the eight regions of the Sustainable Development Goals. We calculated risk of study bias based on the Appraisal tool for Cross-Sectional Studies, and stratified results based on low versus high risk of bias. This systematic review and meta-analysis was registered with PROSPERO, CRD42019144594.

**Findings:**

We reviewed 4339 records, 228 IBBS reports, and ten articles from other sources. Of these, 1301 duplicate records were excluded, 2467 records were excluded after title and abstract screening, and 534 articles were excluded after full-text analysis. We identified 345 prevalence data points from 275 studies across 77 countries, with a total of 606 232 participants. Global pooled prevalence from 2000–20 was 7·5% (95% CI 7·0–8·0%), ranging from 1·9% (1·0–3·1%) in Australia and New Zealand to 10·6% (8·5–12·9%) in Latin America and the Caribbean.

**Interpretation:**

Unacceptably high syphilis prevalence among MSM warrants urgent action.

**Funding:**

Wellcome Trust.

## Introduction

WHO routinely produces global estimates of curable sexually transmitted infections among men and women to provide policy makers and programme managers with evidence that can be used to tailor interventions and to enhance programme monitoring and evaluation. Syphilis is a curable sexually transmitted infection (STI) caused by *Treponema pallidum,* a motile Gram-negative spirochaete. The most recent global prevalence estimate from WHO for syphilis was 0·51% (uncertainty interval 0·43–0·60) among men in 2016.^1^ Stratified by regions of the Sustainable Development Goals (SDGs) as led by the UNDP, syphilis prevalence for men in the general population ranges from 0·01% (0·0–0·02) in Australia and New Zealand to 5·18% (2·83–7·54) in Oceania.^1^ However, syphilis can disproportionately affect men who have sex with men (MSM) due to sexual-network dynamics that include high-risk sexual practices, multiple sex partners, and concurrent use of controlled substances.^2,3^

In 2016, the 69th World Health Assembly adopted three Global Health Sector Strategies to guide the health-sector response to the SDGs. The strategy addressing STIs describes four ambitious targets, one of which is to reduce global syphilis incidence by 90% from 2018 to 2030.^4^ To achieve this goal, countries will need to prioritise reducing syphilis among high-burden groups including MSM. Moreover, reports of increasing syphilis infection among MSM in countries including Australia,^5^ New Zealand,^6^ South Korea,^7^ the UK,^8^ and the USA^9^ are cause for concern and action. Efforts to collate MSM prevalence data would help countries and the global community to set priorities. We therefore aimed to assess the global prevalence of syphilis among MSM using the available literature.

## Methods

### Search strategy and selection criteria

In this global systematic review and meta-analysis, we searched MEDLINE, Embase, LILACS, and AIM databases between April 23, 2019, and Feb 1, 2020, without language restriction, for studies done between Jan 1, 2000, and Feb 1, 2020. Search terms were combinations of syphilis related words (eg, “syphilis”, “*Treponema pallidum*”), sexuality related terms (eg, “gay”, “men who have sex with men”), and the names of each of the 235 countries and territories (one by one) based on SDG regional groupings.^10^ We tested the search strategy through an iterative process before finalising our combinations of terms (appendix p 38). We identified additional point prevalence data by reviewing reference lists from papers that met our inclusion criteria and searching the Key Populations Atlas^11^ and Integrated Bio-Behavioral Surveillance (IBBS) reports from UNAIDS. We inquired with corresponding authors to fill in information gaps if key details were not contained in the full publication.

We defined MSM as individuals who were assigned as male at birth and had oral or anal sex with at least one other man in their lifetime. Eligible studies included bisexual men and transgender individuals, and reported crude syphilis prevalence data based on a biological assay. Studies were excluded if participants were exclusively HIV-infected MSM, injection-drug users, only seeking care for STIs or genital symptoms, or routine STI clinic attendees. These studies would have introduced selection bias and skewed pooled prevalence estimates higher. We followed the PRISMA checklist (appendix pp 22–23) and registered our study with PROSPERO, CRD42019144594.

### Data analysis

The first author (MT) screened titles and abstracts of articles on the basis of eligibility criteria and then extracted relevant information onto a standardised data extraction form (appendix p 39). Two other coauthors (EPD and JE) reviewed all data extraction forms alongside full-text articles, cross-checking for accuracy and validity. Non-English articles were also reviewed. Discrepancies between the full-text and data extraction form were discussed by these three researchers. If consensus could not be reached, the last author (RMC) served as arbiter and made a final determination.

Various diagnostic methods were used across studies reporting point prevalence data, each representing a different degree of diagnostic accuracy (appendix p 24). If a study used more than one diagnostic test, we chose data from qualitative diagnostic methods that were based on a combination of non-treponemal and treponemal tests. If a study used an assay that has a reported range of sensitivity or specificity, we selected the midpoint measure. We then applied a standard method for correcting diagnostic errors for all point prevalence data using the sensitivities and specificities of individual assays (appendix pp 24, 40).^12^ The method allowed for standardising point estimates on the basis of a common definition (the WHO-recommended standard of active syphilis, defined as positivity on both a treponemal and non-treponemal test).^13^ We then applied these corrected point prevalence data to random-effects models using the metaprop command^14^ in Stata, which is capable of incorporating proportions close to or at the margins, ie, very low or very high prevalence data, into pooled prevalence estimates and 95% CIs.^14^ We stratified results into the eight SDG regions and the six WHO regions (appendix pp 3–11, 25–26). We assessed heterogeneity between studies by subgroup analyses using the χ^2^ test with Cochran’s Q statistic and quantified with *I*^2^.^15,16^ We further explored whether observed differences in the corrected prevalence estimates could be explained by other factors using multivariate random-effect meta-regression, adjusting for all the variables of interest. All statistical analyses were done using Stata/IC version 16.1.

We did several subgroup analyses, stratifying syphilis prevalence estimates by 10-year periods (2000–09 and 2010–20) and then 5-year periods (2000–04, 2005–09, 2010–14, 2015–20). We stratified the global pooled prevalence estimates of syphilis by country income level according to World Bank classifications (low, lower-middle, upper-middle, and high),^17^ HIV prevalence of study participants corrected using the same methods as that for syphilis (>5% *vs* ≤5%; appendix p 27), subgroups of MSM (studies exclusively with male sex workers, transgender women, and transgender women sex workers *vs* other MSM studies), legality of same-sex sexual acts in the country from where data came (legal *vs* illegal),^18^ sampling methods used (snowball sampling, respondent-driven sampling, time-location sampling, voluntary counselling and testing, probability sampling, and convenience sampling), diagnostic methods used (qualitative [without cutoff titre value of non-treponemal test] *vs* quantitative [with the value of non-treponemal test]), sample size (>500 *vs* ≤500), and scoring for risk of study bias on the basis of the Appraisal tool for Cross-Sectional Studies (AXIS, high risk [0–10] *vs* low risk [11–20]; appendix p 41).^19,20^ Categories of income level and legality of same-sex sexual acts were based on circumstances within each country or territory at the midpoint of the study period. We further assessed how inclusion or exclusion of each subgroup influenced our overall estimates as a sensitivity analysis.

### Role of the funding source

There was no direct funding source for this study.

## Results

**Figure 1:**
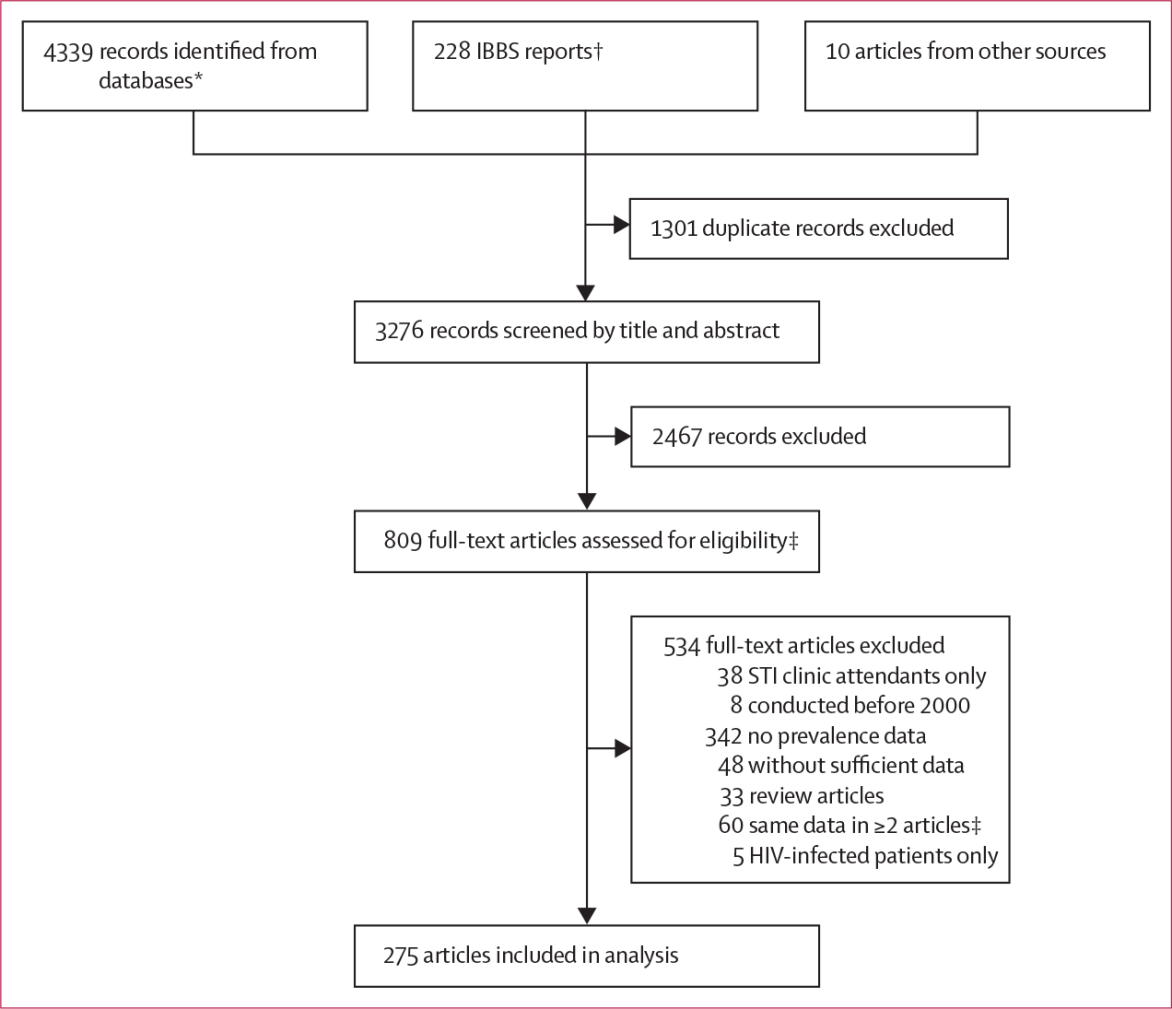
Study selection IBBS=Integrated Bio-Behavioral Surveillance. STI=sexually transmitted infection. *Obtained from MEDLINE, Embase, LILACS, and AIM. †Obtained from search on UNAIDS Key Populations Atlas and consultation with UNAIDS.^11^ ‡Data that appeared in more than one record were reviewed within full-text articles. We included the more or most informative article in the systematic review and meta-analysis, and excluded the others.

We identified 4339 records through searches of MEDLINE, Embase, LILACS, and AIM databases. We also reviewed 228 IBBS reports and obtained ten articles from other sources. In total, we found 275 eligible studies that contained 345 data points for meta-analysis (figure 1).

**Figure 2:**
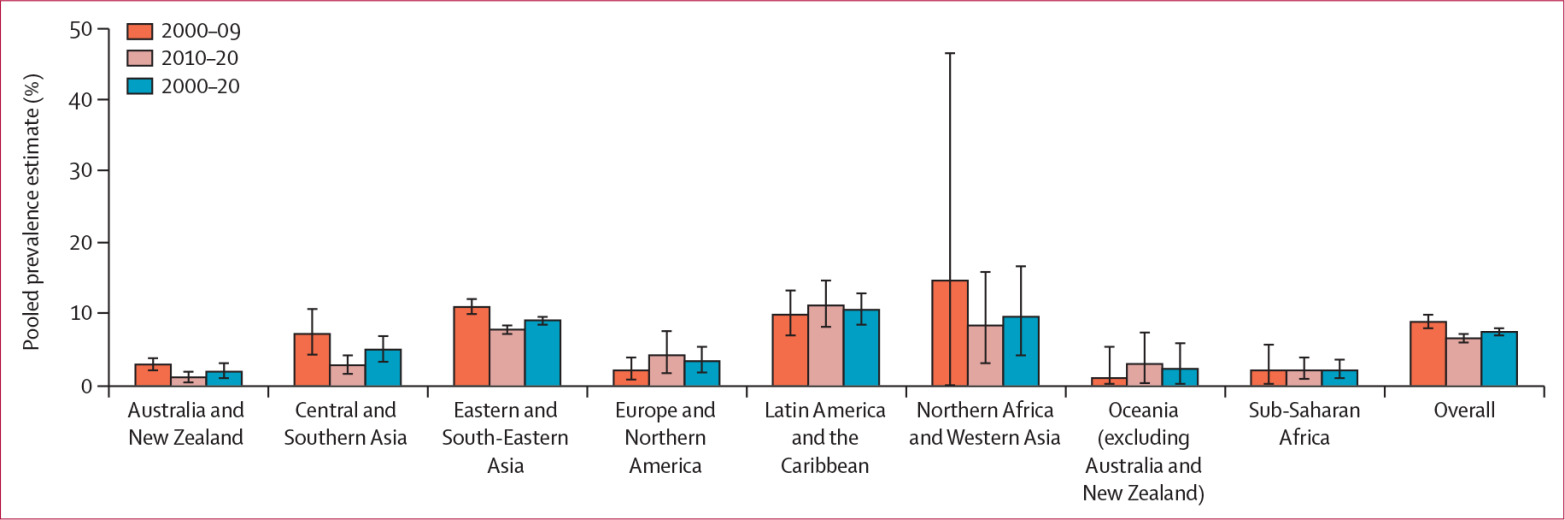
Histogram of pooled syphilis prevalence estimates among MSM, 2000–20, by regions of the Sustainable Development Goals Error bars indicate 95% CI. MSM=men who have sex with men.

**Figure 3:**
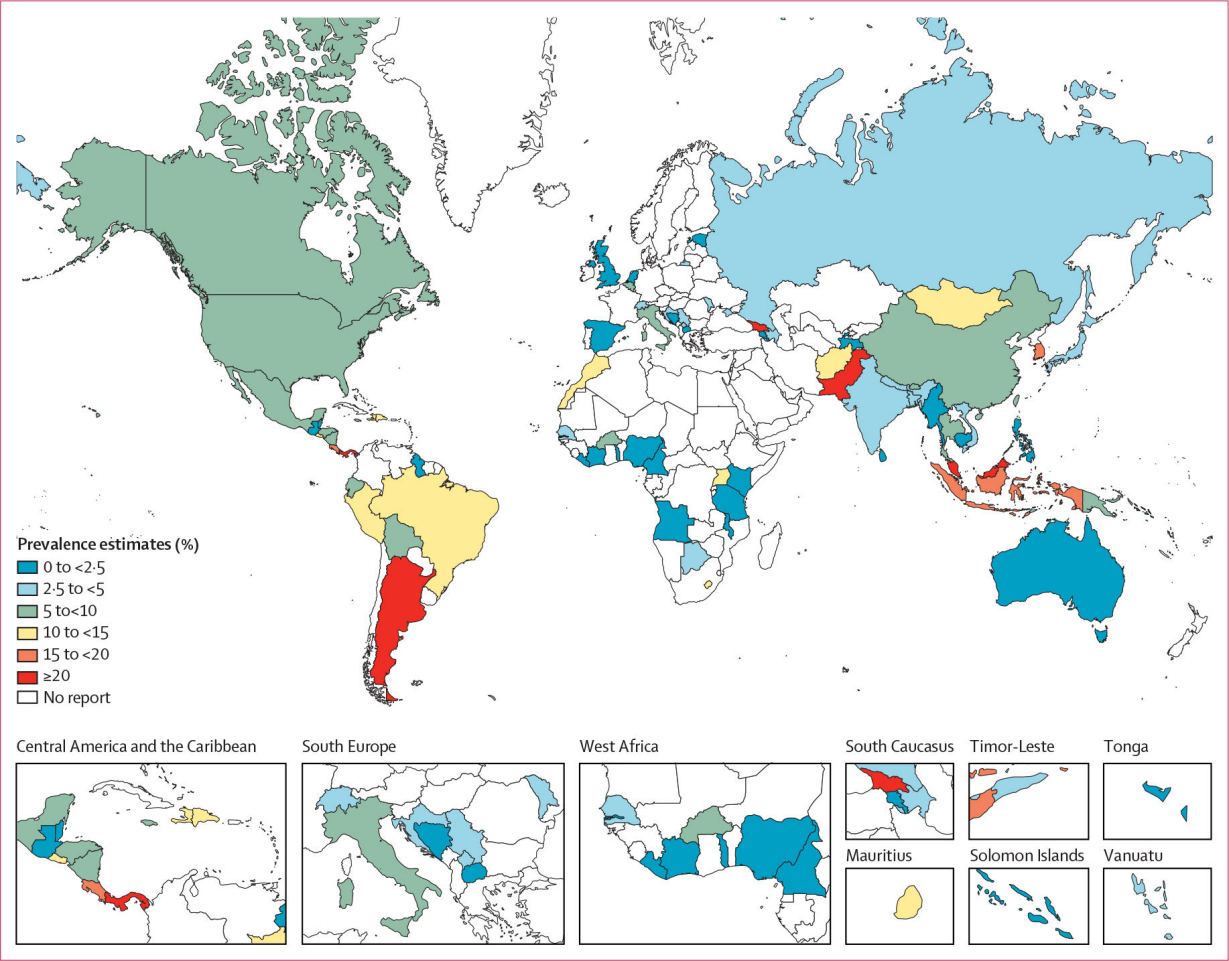
Map of syphilis prevalence estimates among men who have sex with men by country

Global and SDG regional pooled prevalence estimates from 2000–20, 2010–20, and 2000–09 are presented in table 1 and figure 2. Pooled prevalence estimates stratified by SDG region into 5-year intervals can be found in the appendix (p 28). Pooled prevalence estimates by country are shown in figure 3 and the appendix (p 29).

The global pooled syphilis prevalence estimate was 7·5% (95% CI 7·0–8·0, 345 data points; n=606 232) in MSM between 2000 and 2020 (table 1 and figure 2). Three of eight SDG regions (Latin America and the Caribbean, Northern Africa and Western Asia, and Eastern and South-Eastern Asia) had pooled prevalence estimates higher than the global figure. The highest pooled estimate was from Latin America and the Caribbean at 10·6% (8·5–12·9, 49 data points; n=32 316) and the lowest was from Australia and New Zealand at 1·9% (1·0–3·1, 5 data points; n=2494). The pooled prevalence in 2000–09 was slightly higher than that of the overall 20-year period, at 8·9% (8·0–9·9, 145 data points; n=185 877). In 2010–20, the global pooled prevalence was 6·6% (6·0–7·2, 200 data points; n=420 355) which is significantly lower than that in 2000–09 (p<0·0001). Three SDG regions (Northern Africa and Western Asia, Eastern and South-Eastern Asia, and Latin America and the Caribbean) had estimates above the global pooled estimate for 2000–09. The highest regional pooled estimate was 14·7% (0·0–46·6, 3 data points; n=310) in Northern Africa and Western Asia, and the lowest was 1·0% (0·2–5·4, 1 data point; n=100) in Oceania (excluding Australia and New Zealand). As in 2000–09, three SDG regions (Latin America and the Caribbean, Northern Africa and Western Asia, and Eastern and South-Eastern Asia) had pooled prevalence estimates that were higher than the global prevalence estimate in 2010–20 (table 1 and figure 2). The highest estimate was from Latin America and the Caribbean at 11·2% (95% CI 8·2–14·7, 24 data points; n=16 707), and the lowest was from Australia and New Zealand at 1·1% (0·4–1·9, 3 data points; n=915).

The pooled prevalence estimates in four SDG regions (Australia and New Zealand, Central and Southern Asia, Eastern and South-Eastern Asia, and Northern Africa and Western Asia) were lower in 2010–20 than in 2000–09. However, the pooled prevalence estimates for 2015–20 were higher in four SDG regions (Europe and Northern America, Latin America and the Caribbean, Oceania [excluding Australia and New Zealand], and sub-Saharan Africa) compared with 2010–14 (appendix pp 12, 28).

Results from subgroup analyses are in table 2. The lowest pooled prevalence estimate was from low-income countries, at 3·8% (95% CI 2·4–5·4, 48 data points; n=34 440), whereas the highest pooled estimate was from lower-middle income countries, at 8·7% (7·7–9·7, 128 data points; n=186 450). Of 345 data points for syphilis, 331 (95·9%) also reported HIV prevalence. The pooled prevalence of syphilis was 8·7% (95% CI 8·1–9·4, 208 data points; n=444 914) in studies where HIV prevalence was greater than 5%, versus 5·8% (4·9–6·8, 123 data points; n=153 458) in studies that reported an HIV prevalence of 5% or less. Pooled prevalence of studies exclusively among subgroups of MSM (male sex workers, transgender women, and transgender women sex workers) was 16·6% (10·4–23·9, 18 data points; n=7096), although this represented just 1·2% of all MSM tested. Countries where same-sex sexual acts are not prohibited by law had a pooled prevalence of 8·4% (7·9–8·9, 288 data points; n=552 674), compared with 3·7% (2·6–4·9, 57 data points; n=53 558) where same-sex sexual acts are illegal. Convenience sampling was the most common method, accounting for 35·1% (121 data points) of syphilis data points and yielding a pooled prevalence of 7·4% (95% CI 6·5–8·4, n=142 709). Among convenience sampling methods, pooled prevalence estimates based on any combination of multiple convenience sampling methods was 8·7% (7·6–9·9, 64 data points; n=109 065), followed by convenience sampling used by non-profit or charity organisations focused on the needs of MSM or that provide HIV prevention services, at 7·8% (5·2–10·8, 13 data points; n=13 988), venue-specific sampling (eg, saunas and bathhouses, clubs, and one-off public events) at 6·1% (3·7–9·1, 29 data points; n=13 229), and other convenience sampling at 4·7% (1·4–9·4, 15 data points; n=6427). Time-location sampling methods produced the highest pooled prevalence estimate, at 12·3% (8·7–16·5, 8 data points; n=10 359), derived from 2·3% of all data points. The lowest pooled prevalence estimates came from probability sampling, at 6·0% (1·0–14·6, 7 data points; n=3648), which contributed 2·0% of all data points. Studies using qualitative diagnostic methods showed pooled prevalence estimates of 7·7% (7·1–8·2, 314 data points; n=584 191), and inclusion of studies that used quantitative methods did not change overall prevalence estimates (7·5%, 7·0–8·0; 345 data points, n=606 232). Pooled estimates were not different between sample sizes generated with more than 500 participants (7·6%; 95% CI 7·0–8·2, 175 data points, n=555 571) and sample sizes with 500 or fewer participants (7·5%; 6·2–8·9, 170 data points, n=50 661). Stratification of studies using the AXIS classification system (score 0–10 *vs* 11–20), suggests that risk of individual study bias did not alter pooled prevalence estimates. Multivariate random-effects meta-regression subgroup analyses showed that country income level (p=0·0009), HIV prevalence (p=0·0004), subpopulation groups of MSM (p<0·0001), and legality of same-sex acts (p<0·0001) might be a source of heterogeneity. Forest plots for pooled prevalence estimates with corresponding 95% CIs stratified by SDG region and study period are shown in the appendix (pp 13–21).

## Discussion

To our knowledge, this is the first systematic review and meta-analysis assessing global syphilis prevalence among MSM. The number of MSM tested in published studies has increased between 2000 and 2020. While the global syphilis prevalence among MSM was 7·5% over 20 years, SDG regional estimates ranged from 1·9% to 10·6%. Global pooled prevalence estimates were lower in 2010–20 than in 2000–09, whereas pooled prevalence estimates were higher in 2015–20 in four of eight SDG regions compared with 2010–14. Syphilis prevalence estimates were high in studies exclusively involving male sex workers, transgender women, and transgender women sex workers, and studies in which HIV prevalence was greater than 5·0%.

We observed a huge rise in the number of MSM tested in published studies, from 185 877 in 2000–09 to 420 355 in 2010–20, which was reflected by increases in all the SDG regions except for Australia and New Zealand. Improved access to testing since 2000 has been advanced by the availability of rapid point-of-care tests for syphilis, which might have contributed to reducing prevalence.^21,22^ For example, one serial cross-sectional study among HIV-positive MSM (113 272 clinic visits in total) and HIV-negative MSM (246 041 clinic visits in total) in Australia in 2007–14 reported that increased screening was associated with increased detection of early latent syphilis (p<0·0001 for both HIV-positive and HIV-negative MSM), and reductions in secondary syphilis (p=0·0003 for HIV-positive MSM and p=0·03 for HIV-negative MSM).^23,24^

The pooled prevalence of syphilis was the lowest in low-income countries. This observed relationship between wealth and disease burden is difficult to explain. Wealth can increase geographical mobility which, in turn, could increase opportunities for MSM to engage in sexual activity, increasing exposure to syphilis in higher-income settings. However, increased exposure could be offset by better access to syphilis testing and treatment in these same settings. Similarly, the relationship between syphilis prevalence and laws that criminalise same-sex acts, including some that include the death penalty for homosexuality, needs to be interpreted with caution. These laws might have the effect of reducing the willingness of individuals to self-identify as MSM and engage in sexual activity,^18^ limiting exposure to syphilis. Moreover, the same laws could also discourage the most sexually active and at-risk MSM from participating in prevalence surveys. In our analyses, 35 (72·9%) of 48 data points from low-income countries were in sub-Saharan Africa and Central and Southern Asia. Of these, 16 (94·1%) of 17 data points from sub-Saharan Africa, and 13 (72·2%) of 18 data points from Central and Southern Asia were from countries that criminalise same-sex sexual acts.

The highest prevalence of syphilis was reported in lower-middle-income countries, although upper-middle-income and high-income countries are areas of concern, particularly with reports that suggest syphilis transmission among MSM is increasing.^5–9^ Multiple factors are probably at play, including changes in MSM sexual behaviour, as reflected in increasing rates of new partner acquisition and concurrent partnerships, mobility of the MSM population, and reduced use of condoms due to pre-exposure prophylaxis for HIV acquisition.^25,26^ One study suggests that annual screening and treatment of at least 62% of a population of sexually active MSM is necessary to achieve local elimination (defined as less than one case per 100 000 people).^27^ Screening and treatment are interventions that, when coupled with other prevention methods such as consistent condom use and partner notification and treatment, can reduce the burden of syphilis among MSM to achieve the targeted reduction in global syphilis incidence of 90% by 2030.

Our study has several limitations. Firstly, more than third of prevalence data included were based on various convenience sampling methods. However, sub analyses of the corrected pooled prevalence estimates for each category of convenience sampling produced results that were similar to the overall pooled prevalence estimates for convenience sampling, and results from probability sampling were similar to the overall pooled prevalence estimate for MSM. A second limitation is the fact that the region of Eastern and South-Eastern Asia contributed 188 (54·5%) of the 345 global survey data points, mainly from China (153 data points), and had a pooled prevalence of 9·7%. This disproportionate number of data points, including one study with 47 231 MSM,^28^ suggests that syphilis surveillance among MSM is common and prioritised in Eastern and South-Eastern Asia. However, such numbers skew the global pooled prevalence slightly upward. Another limitation relates to high heterogeneity across SDG regions and other subgroupings, despite correcting the data for diagnostic test differences. Of 345 data points, 30 were categorised as being at high risk of bias, and 18 of 345 were from studies limited to MSM who were also male sex workers, transgender women, or transgender women sex workers, who appear to be at higher risk of syphilis infection when compared with other MSM studies. Of 345 data points, 31 were based on quantitative diagnostic methods, which showed lower prevalence estimates than qualitative methods. However, our sensitivity analyses suggest that the prevalence data from these data points did not influence our overall estimates. Lastly, even when considering the corrected prevalence estimates of active syphilis based on the global reference standard definition, a positive result from a non-treponemal test and treponemal test, regardless of non-treponemal titre cutoff, might not represent an incident infection. The reference standard definition of active syphilis can include some infections that are old and already treated. As a result, the proportion of cases at any given time could be influenced by the local historic prevalence of syphilis. In areas where syphilis prevalence has been historically high, we might be overestimating infection, and syphilis outbreaks might not be detectable until the rise of new cases is substantial.

The COVID-19 pandemic has affected MSM sexual behaviours, with evidence that sexual activity among networks of MSM has reduced because of lockdowns. This behaviour change could translate into fewer new syphilis infections in the near term.^29,30^ However, there have also been reports^30^ that MSM have had difficulty accessing STI testing and treatment during the pandemic, potentially suggesting that fewer syphilis cases are being diagnosed, treated, and subtracted from the MSM population. In light of these reports, it is difficult to know if syphilis prevalence among MSM will be indirectly altered by COVID-19. Regardless, syphilis prevalence among MSM is unacceptably high both globally and in SDG regions, particularly in lower-middle-income, upper-middle-income, and high-income countries where cases appear to be increasing and where HIV prevalence is high. Achieving the goal of reducing global syphilis incidence by 90% by 2030 will require sustained commitment to interventions that can accelerate syphilis prevention, screening, and treatment in this population.

## Supplementary Material

Appendix

## Figures and Tables

**Table 1:** Pooled syphilis prevalence estimates among MSM 2000–20 in regions of the Sustainable Development Goals

	Number of MSM with positive syphilis test	Number of MSM tested, n (% of total)	Uncorrected pooled prevalence estimates (95% CI)	Corrected pooled prevalence estimates (95% CI)	Median number of positive diagnoses	Study sample size, range	Number of countries	Number of point prevalence data (% of total)	Heterogeneity *I*^2^*

**2000–20**									
Total	52 067	606 232 (100%)	8·4% (7·9–8·9%)	7·5% (7·0–8·0%)	48	31–47 231	77	345 (100%)	98·1
Australia and New Zealand	60	2494 (0·4%)	2·0% (1·0–3·4%)	1·9% (1·0–3·1%)	6	98–1396	1	5 (1·4%)	58·4
Central and Southern Asia	2008	37 376 (6·2%)	5·5% (3·8–7·5%)	5·0% (3·3–6·9%)	22	42–11 997	8	27 (7·8%)	98·2
Eastern and South-Eastern Asia	43 872	498 371 (82·2%)	9·7% (9·1–10·2%)	9·1% (8·5–9·6%)	66	50–47 231	12	188 (54·5%)	97·6
Europe and Northern America	763	13 618 (2·2%)	5·7% (3·7–8·1%)	3·4% (1·8–5·4%)	13	31–2296	15	34 (9·9%)	96·6
Latin America and the Caribbean	4144	32 316 (5·3%)	11·4% (9·5–13·5%)	10·6% (8·5–12·9%)	54	62–5101	17	49 (14·2%)	97·5
Northern Africa and Western Asia	596	4965 (0·8%)	11·5% (5·5–19·3%)	9·6% (4·2–16·7%)	32	70–700	4	14 (4·1%)	98·2
Oceania (excluding Australia and New Zealand)	51	1038 (0·2%)	2·3% (0·2–5·9%)	2·3% (0·2–5·9%)	1	39–860	4	4 (1·2%)	66·3
Sub-Saharan Africa	573	16 054 (2·6%)	2·9% (1·6–4·6%)	2·1% (1·0–3·6%)	7	109–2442	16	24 (7·0%)	96·7
**2010–20**									
Subtotal	32 366	420 355 (100%)	7·5% (6·9–8·0%)	6·6% (6·0–7·2%)	43	39–42 680	61	200 (100%)	98·0
Australia and New Zealand	11	915 (0·2%)	1·1% (0·4–1·9%)	1·1% (0·4–1·9%)	4	98–617	1	3 (1·5%)	··
Central and Southern Asia	635	20 978 (5·0%)	3·3% (2·1·47%)	2·8% (1·6–4·2%)	16	42–11 997	7	13 (6·5%)	95·0
Eastern and South-Eastern Asia	27 830	354 858 (84·4%)	8·3% (7·8–8·9%)	7·8% (7·2–8·4%)	71	102–42 680	9	107 (53·5%)	97·3
Europe and Northern America	570	8319 (2·0%)	6·5% (3·4–10·3%)	4·2% (1·7–7·6%)	15	43–2296	12	21 (10·5%)	97·5
Latin America and the Caribbean	2249	16 707 (4·0%)	12·2% (9·2–15·5%)	11·2% (8·2–14·7%)	52	62–5101	12	24 (12·0%)	97·5
Northern Africa and Western Asia	525	4655 (1·1%)	9·5% (3·6–17·7%)	8·4% (3·1–15·9%)	60	216–700	4	11 (5·5%)	98·4
Oceania (excluding Australia and New Zealand)	50	938 (0·2%)	3·0% (0·3–7·4%)	3·0% (0·3–7·4%)	1	39–860	3	3 (1·5%)	··
Sub-Saharan Africa	496	12 985 (3·1%)	3·1% (1·6–5·1%)	2·1% (0·9–3·9%)	7	109–2442	13	18 (9·0%)	97·0
**2000–09**									
Subtotal	19 701	185 877 (100%)	9·8% (8·9–10·8%)	8·9% (8·0–9·9%)	54	31–47 231	39	145 (100%)	978
Australia and New Zealand	49	1579 (0·8%)	3·0% (2·2–3·9%)	2·9% (2·1–3·8%)	25	183–1396	1	2 (1·4%)	··
Central and Southern Asia	1373	16 398 (8·8%)	7·7% (4·8–11·3%)	7·2% (4·3–10·7%)	62	132–3739	4	14 (9·7%)	98·3
Eastern and South-Eastern Asia	16 042	143 513 (77·2%)	11·6% (10·6–12·7%)	11·0% (10·0–12·1%)	63	50–47 231	10	81 (55·9%)	97·1
Europe and Northern America	193	5299 (2·9%)	4·3% (2·4–6·7%)	2·1% (0·8–3·9%)	12	31–1387	7	13 (9·0%)	91·1
Latin America and the Caribbean	1895	15 609 (8·4%)	10·7% (8·1–13·6%)	9·9% (7·0–13·3%)	54	78–3280	9	25 (17·2%)	97·6
Northern Africa and Western Asia	71	310 (0·2%)	21·1% (7·7–38·7%)	14·7% (0·0–46·6%)	19	70–140	2	3 (2·1%)	··
Oceania (excluding Australia and New Zealand)	1	100 (0·1%)	1·0% (0·2–5·4%)	1·0% (0·2–5·4%)	1	100–100	1	1 (0·7%)	··
Sub-Saharan Africa	77	3069 (1·7%)	2·4% (0·4–5·8%)	2·1% (0·2–5·7%)	12	290–879	5	6 (4·1%)	96·3

MSM=men who have sex with men.

* Heterogeneity between subgroups in all subgroup analyses was p<0·0001.

**Table 2:** Subgroup analyses of pooled syphilis prevalence estimates among MSM, 2000–20

	Number of MSM with positive syphilis test	Number of MSM tested (% of total)	Uncorrected pooled prevalence estimates (95% CI)	Corrected pooled prevalence estimates (95% CI)	Median number of positive diagnoses	Study sample size, range	Number of countries	Number of point prevalence data (% of total)	Heterogeneity *I*^2^

Country income level*									
Low	2186	34 440 (5·7%)	4·9% (3·5–6·5%)	3·8% (2·4–5·4%)	18	99–2442	21	48 (13·9)	98·1
Lower-middle	18711	186 450 (30·8%)	9·6% (8·6–10·6%)	8·7% (7·7–9·7%)	54	31–47 231	34	128 (37·1)	98·2
Upper-middle	30 469	372 495 (61·4%)	9·2% (8·6–9·9%)	8·6% (8·0–9·2%)	66	50–42 680	25	142 (41·2)	97·5
High	701	12 847 (2·1%)	5·8% (3·6–8·5%)	4·5% (2·6–6·9%)	15	43–2296	14	27 (7·8)	96·9
HIV prevalence									
≤5%	13 496	153 458 (25·3%)	6·8% (5·9–7·8%)	5·8% (4·9–6·8%)	31	31–47 231	41	123 (35·7)	98·1
>5%	37 987	444 914 (73·4%)	9·5% (8·9–10·1%)	8·7% (8·1–9·4%)	61	43–42 680	48	208 (60·3)	98·1
Not available	584	7860 (1·3%)	6·7% (4·0–10·0%)	5·2% (2·4–8·9%)	27	83–1387	10	14 (4·1)	97·5
Subpopulation groups of MSM									
Studies exclusively with male sex workers, transgender women, transgender women sex workers	1269	7096 (1·2%)	17·6% (11·5–24·6%)	16·6% (10·4–23·9%)	53	50–980	14	18 (5·2)	98·3
Other MSM study	50 798	599 136 (98·8%)	8·0% (7·5–8·5%)	7·1% (6·6–7·6%)	48	31–47 231	72	327 (94·8)	98·0
Legality of same-sex acts†									
Legal	49 348	552 674 (91·2%)	9·3% (8·8–9·8%)	8·4% (7·9–8·9%)	54	31–47 231	49	288 (83·5%)	97·8
Illegal	2719	53 558 (8·8%)	4·6% (3·5–5·9%)	3·7% (2·6–4·9%)	17	39–11 997	29	57 (16·5%)	97·9
Sampling methods									
Snowball sampling	32 113	366 855 (60·5%)	9·3% (8·6–10·0%)	8·5% (7·8–9·2%)	66	83–47 231	12	93 (27·0%)	97·9
Response-driven sampling	5042	67 259 (11·1%)	7·5% (6·1–9·0%)	6·7% (5·3–8·2%)	25	39–11 997	54	99 (28·7%)	98·3
Time-location sampling	1143	10 359 (1·7%)	12·4% (8·8–16·7%)	12·3% (8·7–16·5%)	90	108–3739	4	8 (2·3%)	96·7
Volunteer counselling and testing	1619	15 402 (2·5%)	7·4% (4·7–10·6%)	6·9% (4·3–10·0%)	43	90–3040	8	17 (4·9%)	97·9
Probability sampling	421	3648 (0·6%)	9·0% (3·2–17·2%)	6·0% (1·0–14·6%)	49	85–1279	7	7 (2·0%)	98·5
Convenience sampling‡	11 729	142 709 (23·5%)	8·5% (7·5–9·4%)	7·4% (6·5–8·4%)	41	31–32 701	30	121 (35·1%)	97·8
Diagnostic methods									
Qualitative methods	50 789	584 191 (96·4%)	8·7% (8·1–9·2%)	7·7% (7·1–8·2%)	53	31–47 231	74	314 (91·0%)	98·1
Quantitative methods	1278	22 041 (3·6%)	6·0% (4·3–8·1%)	6·0% (4·3–8·1%)	24	39–2276	16	31 (9·0%)	97·0
Sample size									
≤500	5163	50 661 (8·4%)	8·8% (7·5–10·1%)	7·5% (6·2–8·9%)	21	31–500	59	170 (49·3%)	96·8
>500	46 904	555 571 (91·6%)	8·1% (7·6–8·7%)	7·6% (7·0–8·2%)	104	502–47 231	40	175 (50·7%)	98·6
AXIS§ score classification									
Low risk of bias	47 604	551 596 (91·0%)	8·4% (7·9–9·0%)	7·5% (6·9–8·0%)	49	31–47 231	73	315 (91·3%)	98·2
High risk of bias	4463	54 636 (9·0%)	8·3% (6·7–10·1%)	7·8% (6·2–9·6%)	41	62–37 084	16	30 (8·7%)	96·4
Total	52 067	606 232 (100%)	8·4% (7·9–8·9%)	7·5% (7·0–8·0%)	48	31–47 231	77	345 (100%)	98·1

Heterogeneity between subgroups in all subgroup analyses was p<0·0001, except for subpopulation groups of MSM (p=0·0014), sampling methods (p=0·035), diagnostic methods (p=0·129), sample size (p=0·873), and AXIS score (p=0·688). MSM=men who have sex with men.

* Income levels by World Bank classification at the midpoint of the study period.^18^

† Classification of legal or illegal was based on the situation of the country at the midpoint of the study period.

‡ Convenience sampling included data collected by non-profit or charity organisations focused on the needs of MSM or that provide HIV prevention services, venue-specific sampling (eg, saunas and bathhouses, clubs, and one-off public events), via internet advertisement, peer recruitment, and any combination of multiple convenience sampling methods.

§ Appraisal tool for Cross-Sectional Studies (risk of study bias assessment).

## Data Availability

The study protocol and statistical analysis plan are publicly available at PROSPERO (CRD42019144594). Prevalence data points used are provided in the appendix (pp 30–37). Data related to income level of countries, and areas and in-country legality of same-sex acts are publicly available from the World Bank and the International Lesbian, Gay, Bisexual, Trans and Intersex Association.

## Research in context

### Evidence before this study

We searched MEDLINE, Embase, LILACS, and AIM databases, and the Key Population Atlas and Integrated Bio-Behavioral Surveillance reports from UNAIDS, without language restriction, to identify point prevalence estimates of syphilis based on biological assays between April 23, 2019, and Feb 1, 2020, to identify studies done between Jan 1, 2000, and Feb 1, 2020, that contained syphilis point prevalence data among men who have sex with men (MSM). We combined syphilis related word (eg, “syphilis”, “*Treponema pallidum*”), sexuality related terms (eg, “gay”, “men who have sex with men”), and individual names of 235 countries and territories (one by one) worldwide that constitute the regional groupings of the Sustainable Development Goals. We found no existing systematic reviews of global pooled prevalence estimates of syphilis among MSM.

### Added value of this study

To the best of our knowledge, this is the first systematic review and meta-analysis of global syphilis prevalence among MSM. We provide pooled prevalence estimates of syphilis in MSM for the eight regions of the Sustainable Development Goals and six WHO regions over 20 years. Our results underscore the disproportionately high burden of syphilis in MSM. The most recent global estimate for syphilis among men in the general population was from 2016, at 0·5% (uncertainty interval 0·4–0·6) with regional values ranging from 0·01% to 5·18%. By contrast, our global estimate for MSM was 7·5% (95% CI 7·0–8·0) with regional values ranging from 1·9% to 10·6%. We also present global pooled prevalence estimates stratified by country income level according to World Bank classifications (low, lower-middle, upper-middle, and high), HIV prevalence of study participants (>5% *vs* ≤5%), subgroups of MSM (studies exclusively with male sex workers, transgender women, and transgender women sex workers *vs* other MSM studies), in-country legality of same-sex acts, sampling methods used (snowball sampling, respondent-driven sampling, time-location sampling, voluntary counselling and testing, probability sampling, and convenience sampling), diagnostic methods used (qualitative *vs* quantitative methods), sample size (>500 *vs* ≤500), and risk of study bias based on the Appraisal tool for Cross-Sectional Studies (low *vs* high risk of bias).

### Implications of all the available evidence

The global syphilis prevalence among MSM from 2000–20 is alarmingly high. To reduce syphilis incidence 90% globally from 2018 to 2030, as per the Global Health Sector Strategy, will require interventions that target MSM with high rates of annual screening and treatment for syphilis, particularly in lower-middle, upper-middle, and high-income countries where cases appear to be increasing, and where HIV prevalence in MSM is greater than 5%.
