# Supplementary material for: Prevalence of syphilis among men who have sex with men: a global systematic review and meta-analysis from 2000–20
Source: Lancet Glob Health. Author manuscript; Available in PMC 2022 May 30. (PMC9150735; doi:10.1016/S2214-109X(21)00221-7)
Supplement: Appendix [file NIHMS1804808-supplement-Appendix.pdf]

THE LANCET
Global Health

Supplementary appendix

This appendix formed part of the original submission and has been peer reviewed. We post it as supplied by the authors.

Supplement to: Tsuboi M, Evans J, Davies EP, et al. Prevalence of syphilis among men who have sex with men: a global systematic review and meta-analysis from 2000–20. *Lancet Glob Health* 2021; published online July 8. http://dx.doi.org/10.1016/S2214-109X(21)00221-7.

Supplementary appendix

Supplement to: Motoyuki Tsuboi, Jayne Evans, Ella P Davies, Jane Rowley, Eline L Korenromp, Tim Clayton, Melanie M Taylor, David Mabey, R Matthew Chico; **“**Prevalence of syphilis among men who have sex with men: A global systematic review and meta-analysis from 2000 to 2020.”

Supplementary Figures

Figure S1**.** Histogram of pooled syphilis prevalence estimates among MSM by regions of the World Health Organization between 2000-2020

Figure S2**.** Histogram of pooled syphilis prevalence estimates among MSM by regions of the World Health Organization between 2000-2020 in five-year intervals

Figure S3**.** Forest plots of syphilis prevalence estiamtes among MSM by regions of the World Health Organization between 2000-2020

- ***Figure S3.1*****.** Americas
- ***Figure S3.2*****.** Africa
- ***Figure S3.3*****.** Eastern Mediterranean
- ***Figure S3.4*****.** Europe
- ***Figure S3.5*****.** South-East Asia
- ***Figure S3-6a*****.** Western Pacific between 2000-2009
- ***Figure S3-6b*****.** Western Pacific between 2010-2020

Figure S4**.** Histogram of pooled syphilis prevalence estimates among MSM by regions of the Sustainable Development Goals between 2000-2020 in five-year intervals

Figure S5**.** Forest plots of syphilis prevalence estimates among MSM by regions of the Sustainable Development Goals between 2000-2020

- ***Figure S5.1*****.** Australia and New Zealand
- ***Figure S5.2*****.** Central and Southern Asia
- ***Figure S5.3a*****.** Eastern and South-Eastern Asia between 2000-2009
- ***Figure S5.3b*****.** Eastern and South-Eastern Asia between 2010-2020
- ***Figure S5.4*****.** Europe and Northern America
- ***Figure S5.5*****.** Latin America and the Caribbean
- ***Figure S5.6*****.** Northern Africa and Western Asia
- ***Figure S5.7*****.** Oceania (excluding Australia and New Zealand)
- ***Figure S5.8*****.** Sub-Saharan Africa

Supplementary Tables

Table S1**.** PRISMA Checklist

Table S2**.** Sensitivity and specificity of syphilis diagnostic assays used in individual studies

Table S3**.** Pooled syphilis prevalence estimates among MSM in regions of the World Health Organization between 2000-2020

Table S4**.** Pooled syphilis prevalence estimates among MSM in regions of the World Health Organization between 2000-2020 in five-year intervals

Table S5**.** Sensitivity and specificity of HIV diagnostic assays used in individual studies

Table S6. Pooled syphilis prevalence estimates among MSM in regions of the Sustainable Development Goals between 2000-2020 in five-year intervals

Table S7. Pooled syphilis prevalence estimates among MSM by country between 2000-2020

Table S8. Individual studies identified by systematic review containing syphilis prevalence estimates among MSM

Supplementary Text

Text S1**.** Search terms

Text S2**.** Data extraction form

Text S3**.** Correction of observed prevalence

Text S4**.** Appraisal tool for cross-sectional studies (AXIS)

Text S5**.** References

Figure S1. Histogram of pooled syphilis prevalence estimates among MSM by regions of the World Health Organization between 2000-2020

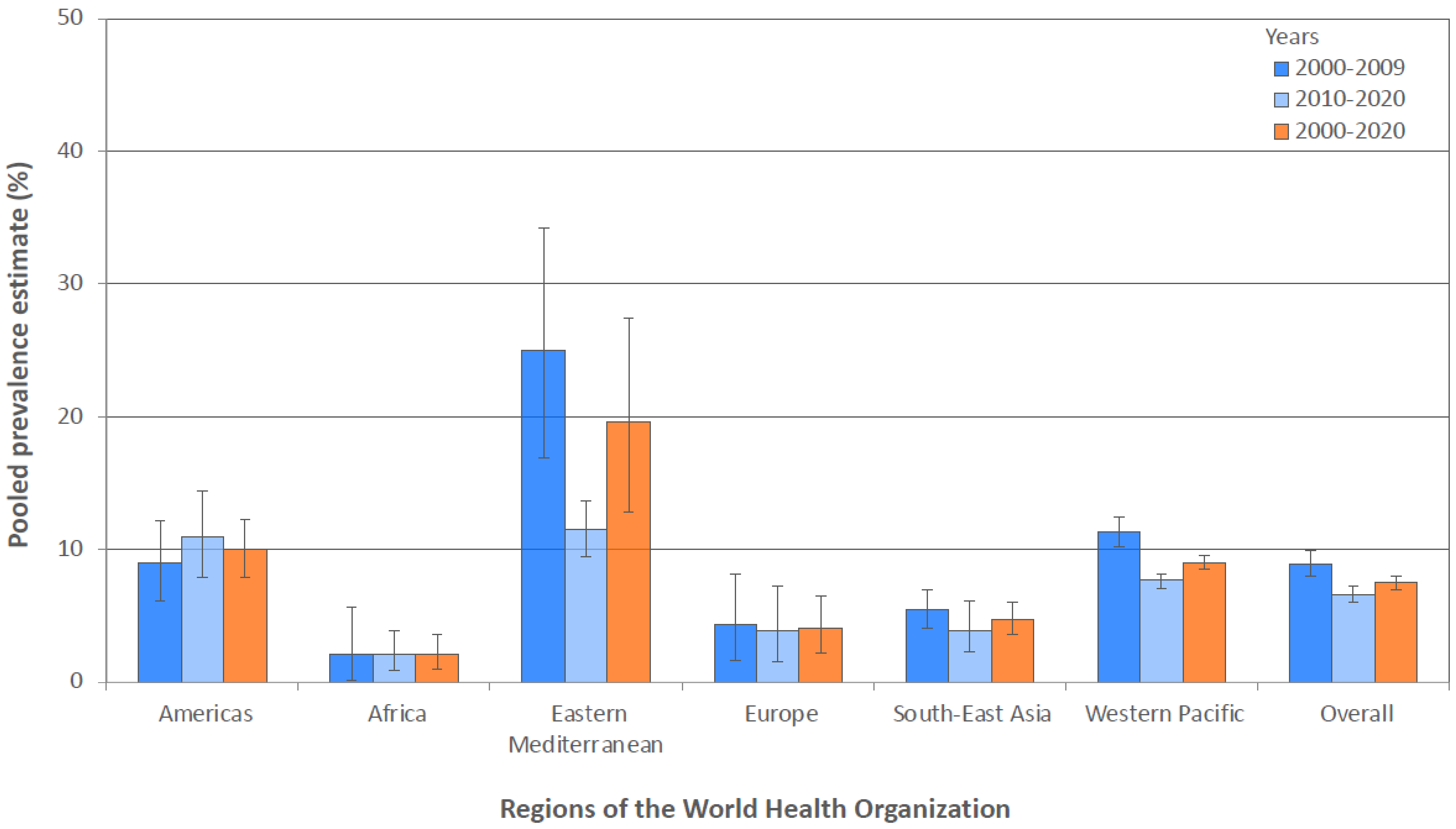

Figure S2. Histogram of pooled syphilis prevalence estimates among MSM by regions of the World Health Organization between 2000-2020 in five-year intervals

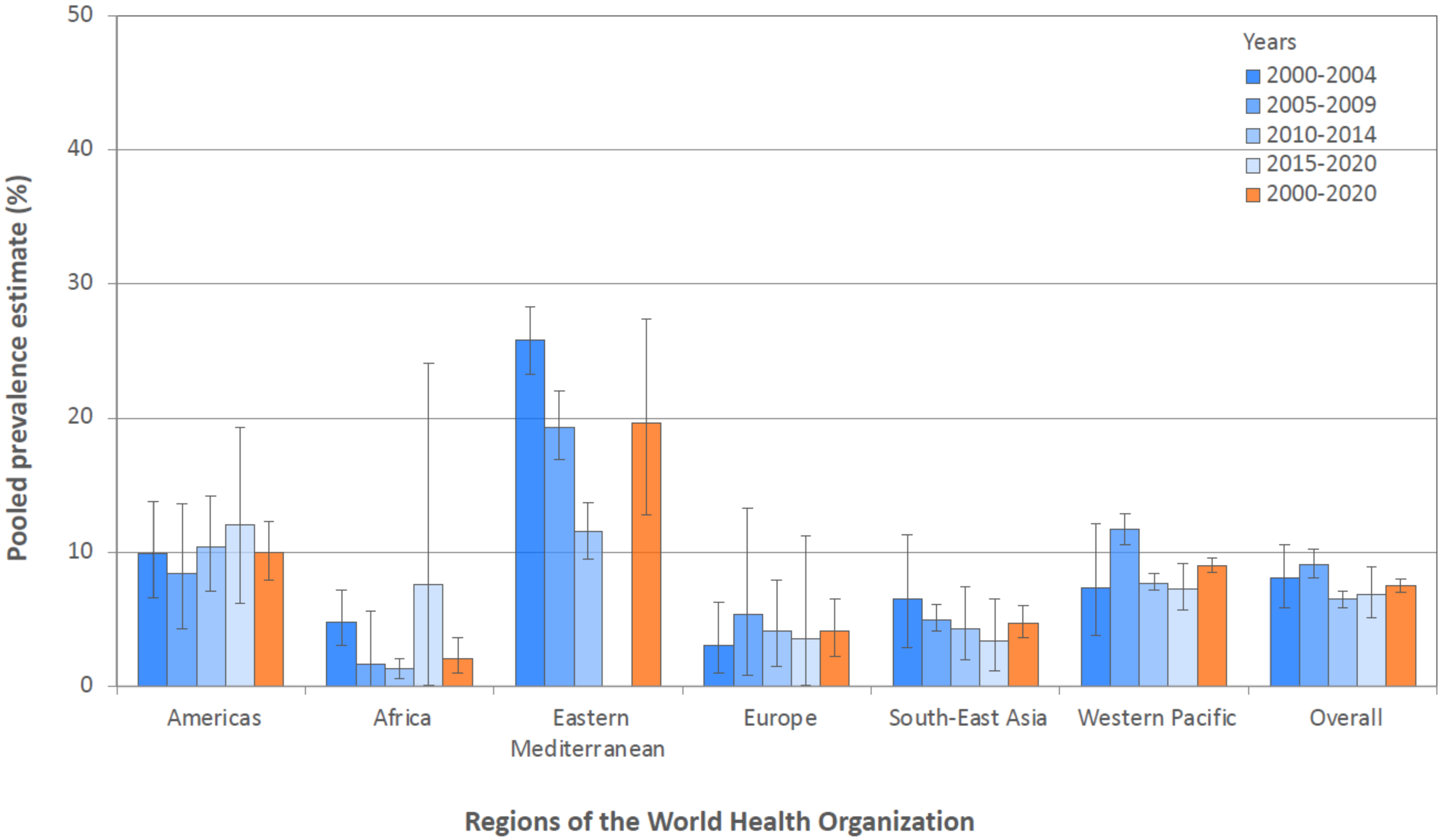

Figure S3.1. Americas WHO Region: Pooled syphilis prevalence estimates among MSM between 2000-2020

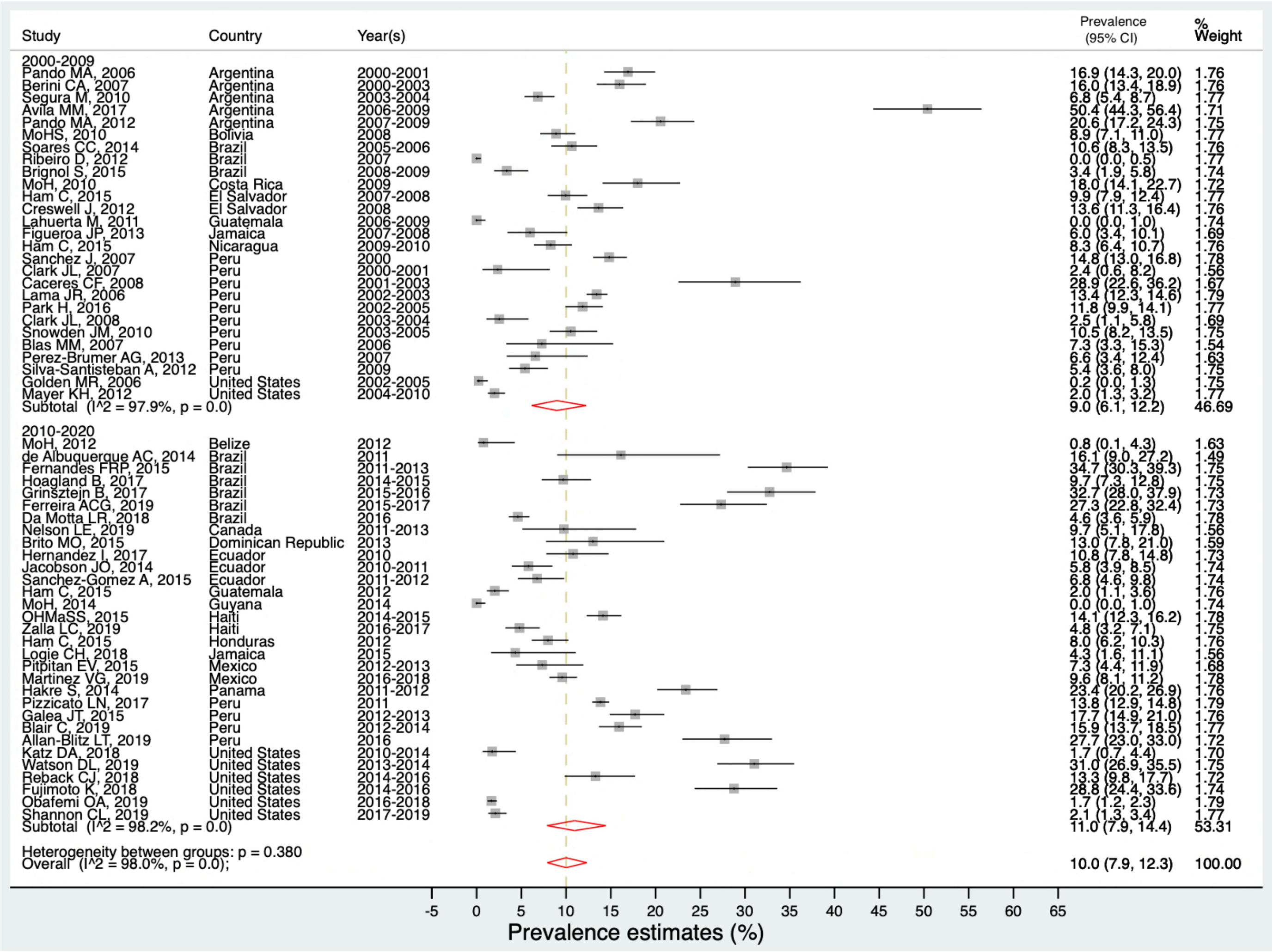

Diamonds indicate pooled mean prevalence estimates; the extremes of the diamonds indicate 95% CIs

Figure S3.2. Africa WHO Region: Pooled syphilis prevalence estimates among MSM between 2000-2020

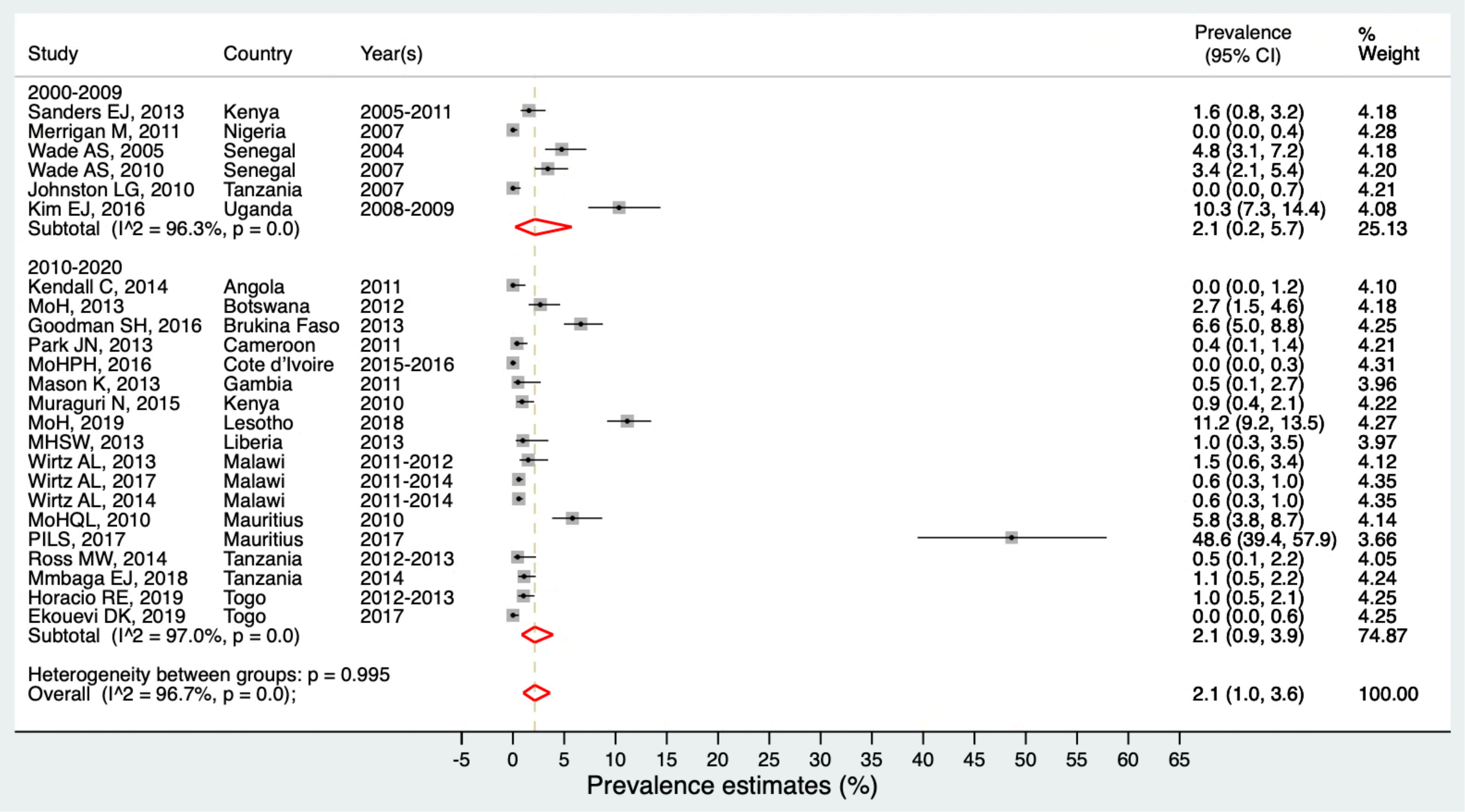

Diamonds indicate pooled mean prevalence estimates; the extremes of the diamonds indicate 95% CIs

Figure S3.3. Eastern Mediterranean WHO Region: Pooled syphilis prevalence estimates among MSM between 2000-2020

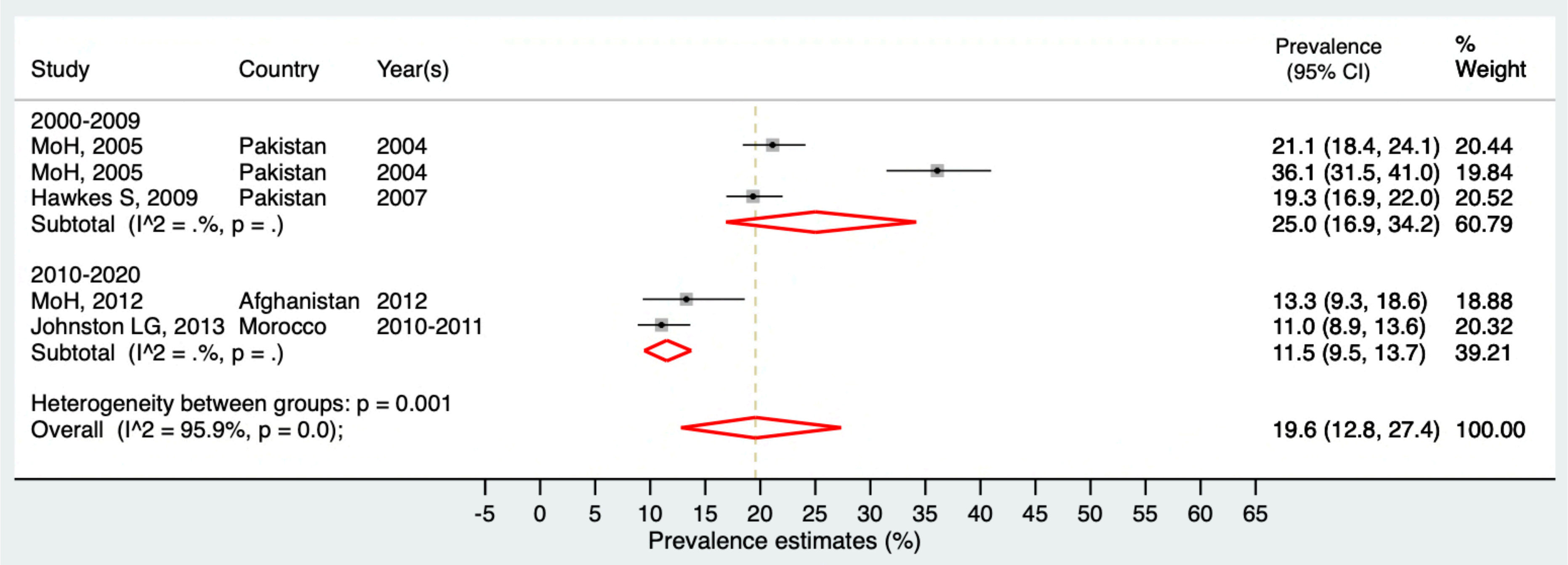

Diamonds indicate pooled mean prevalence estimates; the extremes of the diamonds indicate 95% CIs

Figure S3.4. Europe WHO Region: Pooled syphilis prevalence estimates among MSM between 2000-2020

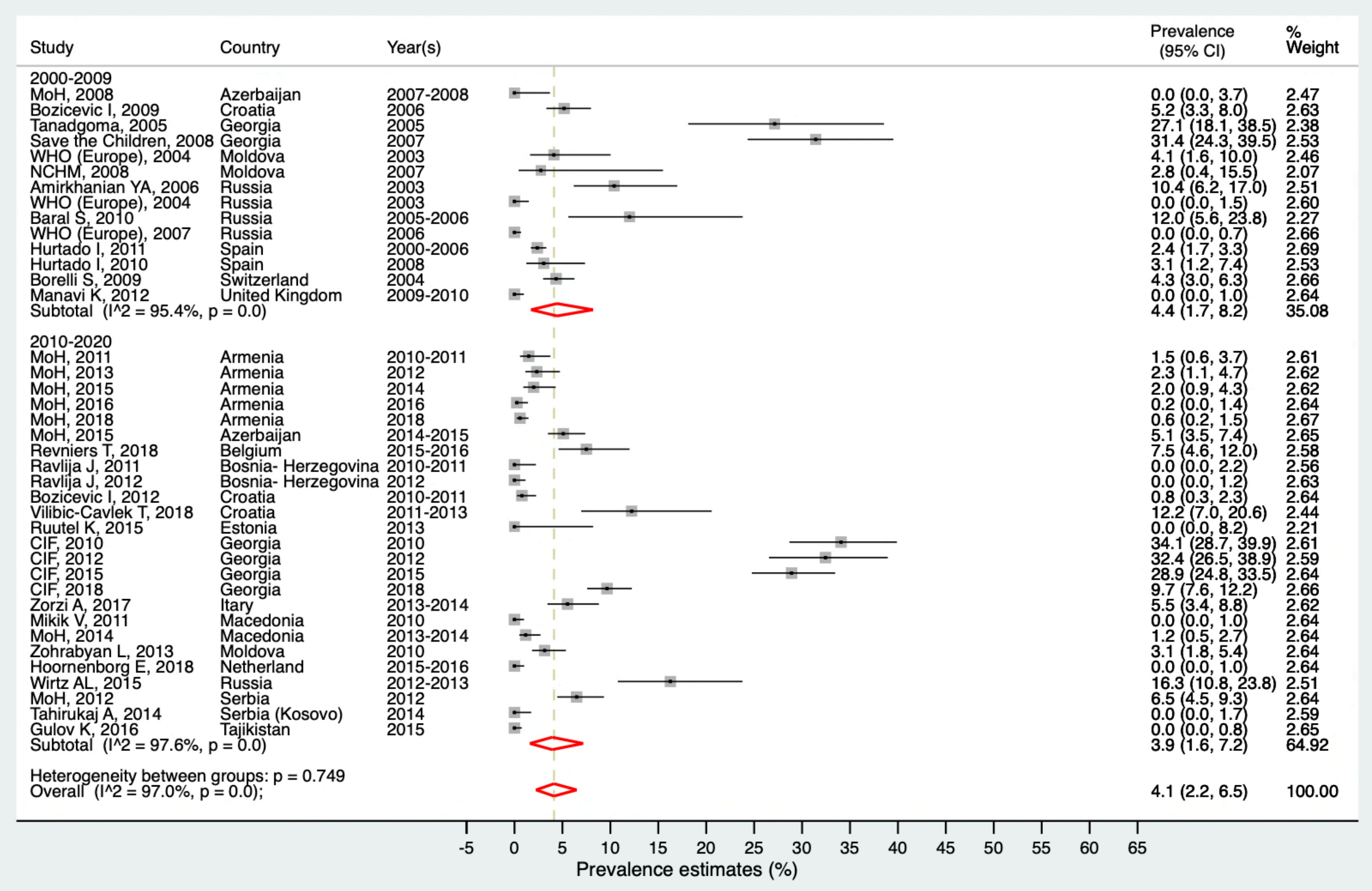

Diamonds indicate pooled mean prevalence estimates; the extremes of the diamonds indicate 95% CIs

Figure S3.5. South-East Asia WHO Region: Pooled syphilis prevalence estimates among MSM between 2000-2020

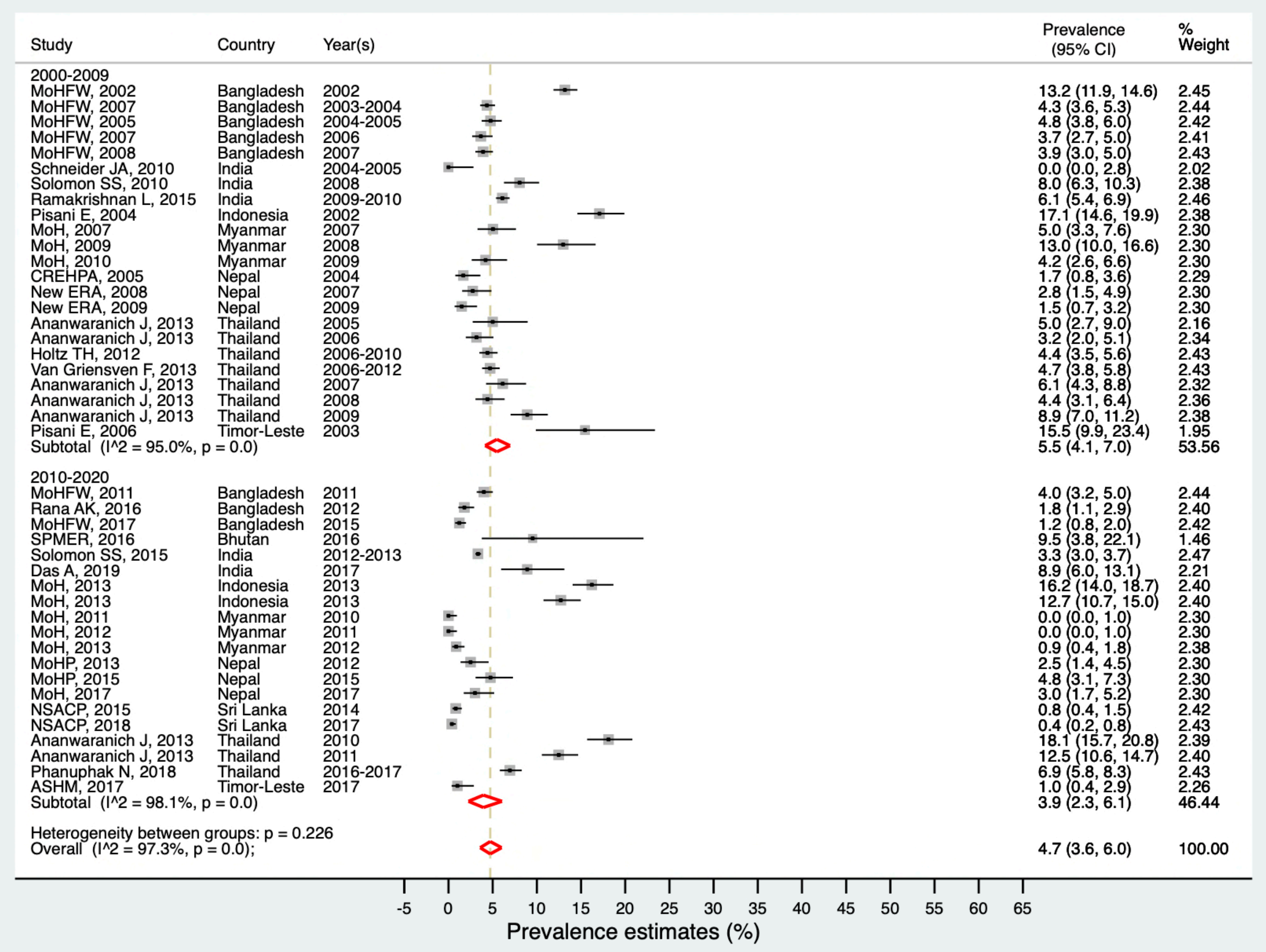

Diamonds indicate pooled mean prevalence estimates; the extremes of the diamonds indicate 95% CIs

Figure S3-6a. Western Pacific WHO Region: Pooled syphilis prevalence estimates among MSM between 2000-2009

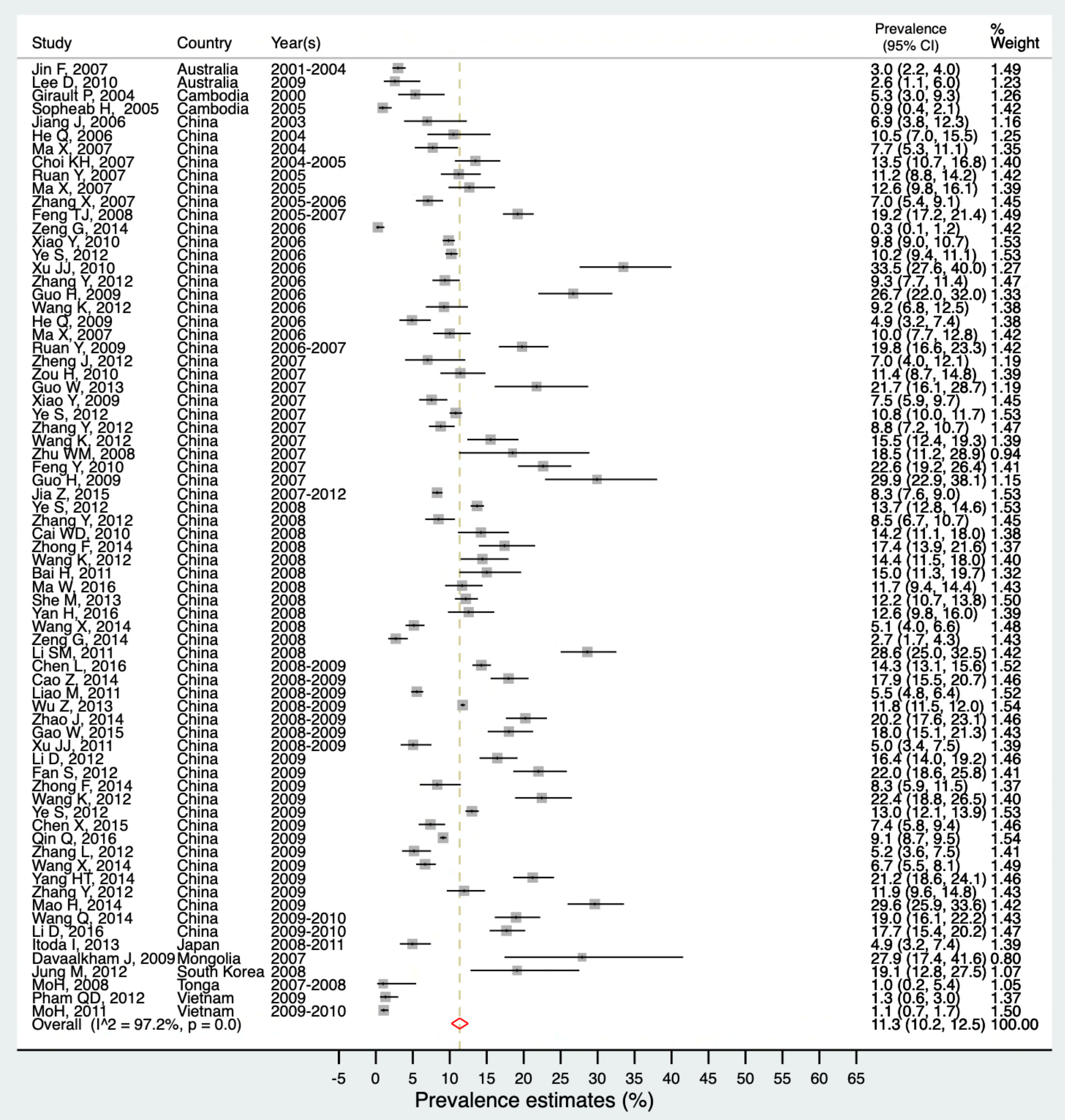

Diamonds indicate pooled mean prevalence estimates; the extremes of the diamonds indicate 95% CIs

Figure S3-6b. Western Pacific WHO Region: Pooled syphilis prevalence estimates among MSM between 2010-2020

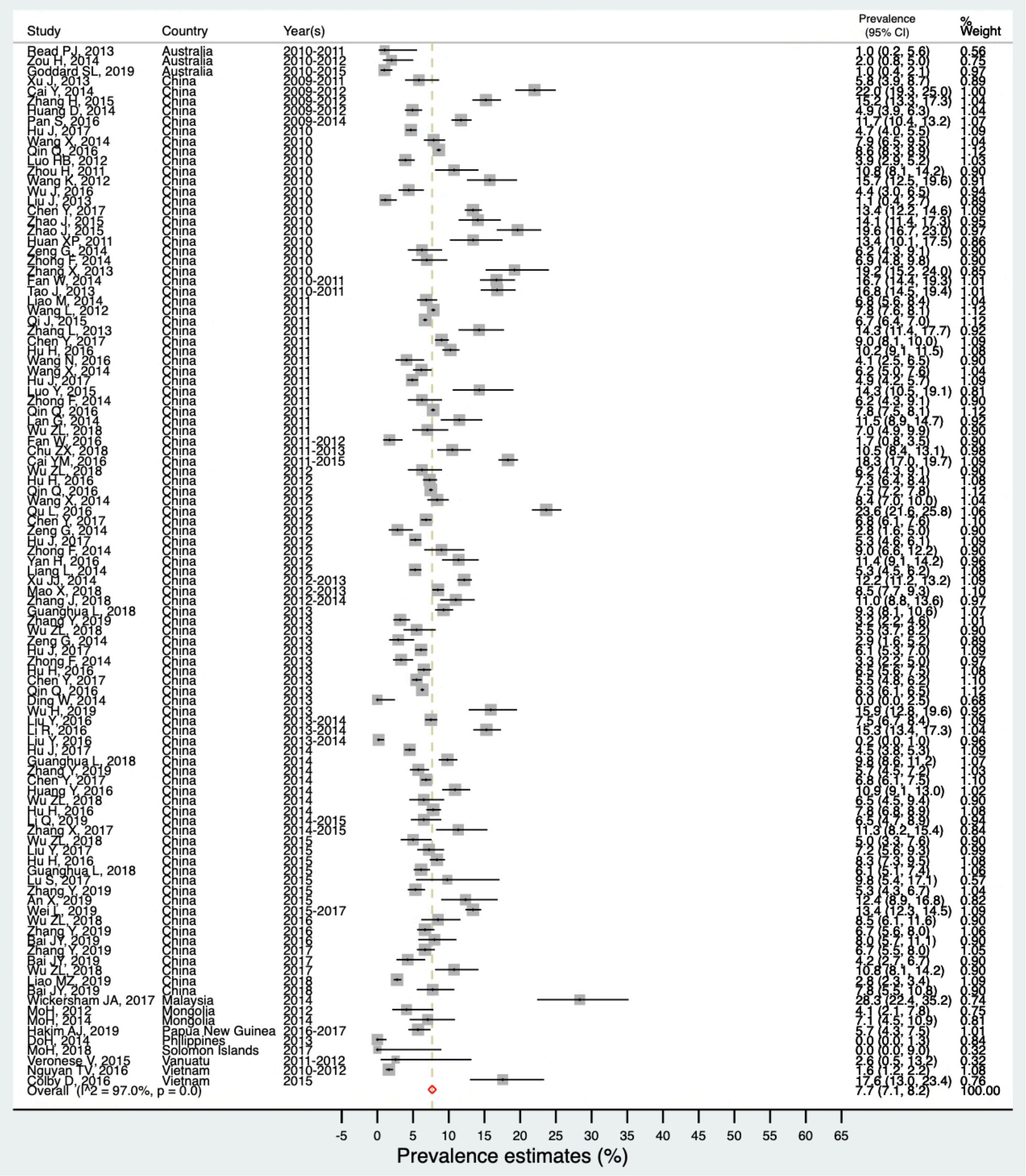

Diamonds indicate pooled mean prevalence estimates; the extremes of the diamonds indicate 95% CIs

Figure S4. Histogram of pooled syphilis prevalence estimates among MSM by regions of the Sustainable Development Goals between 2000-2020 in five-year intervals

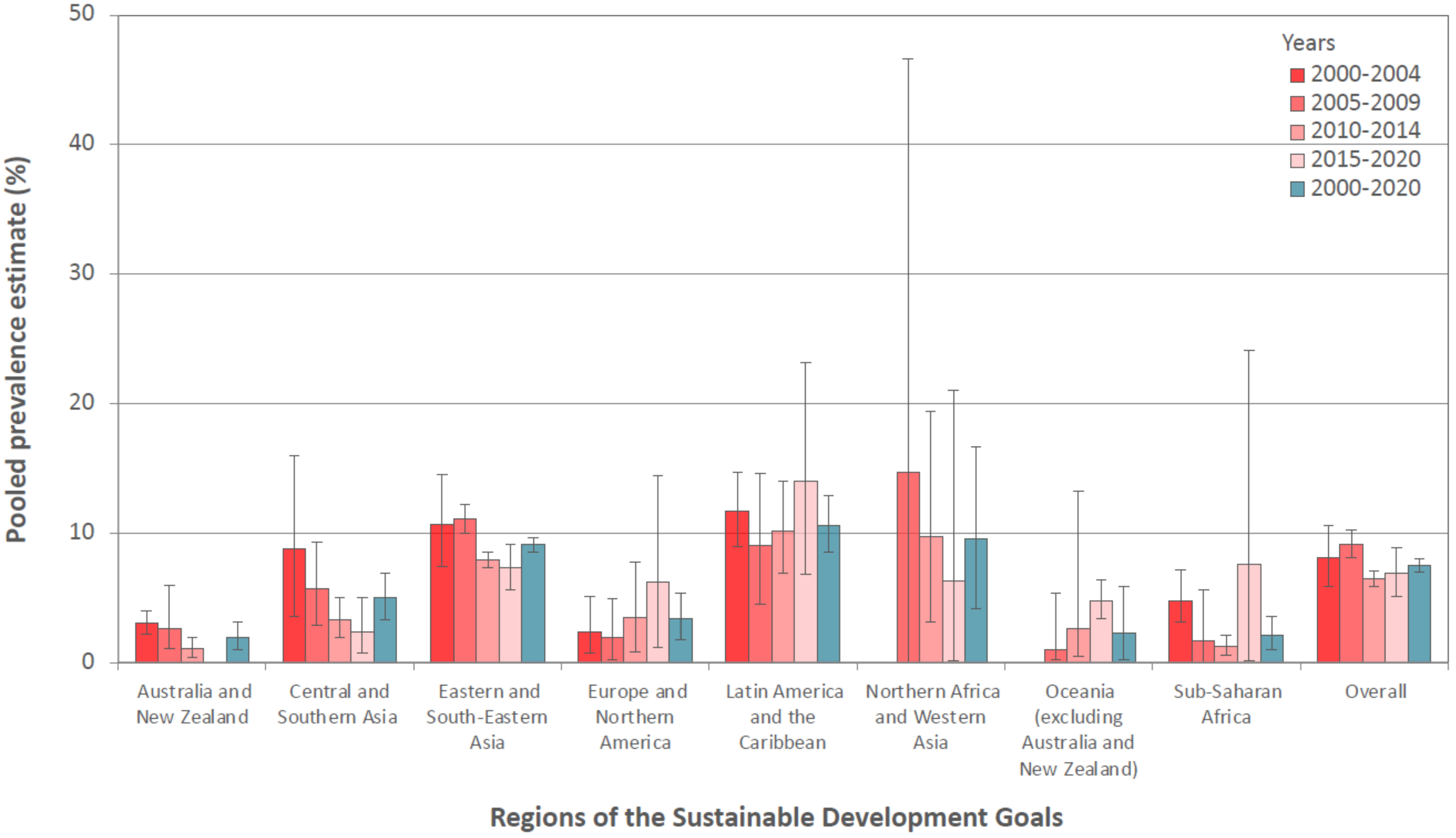

Figure S5.1. Australia and New Zealand SDG Region: Pooled syphilis prevalence estimates among MSM between 2000-2020

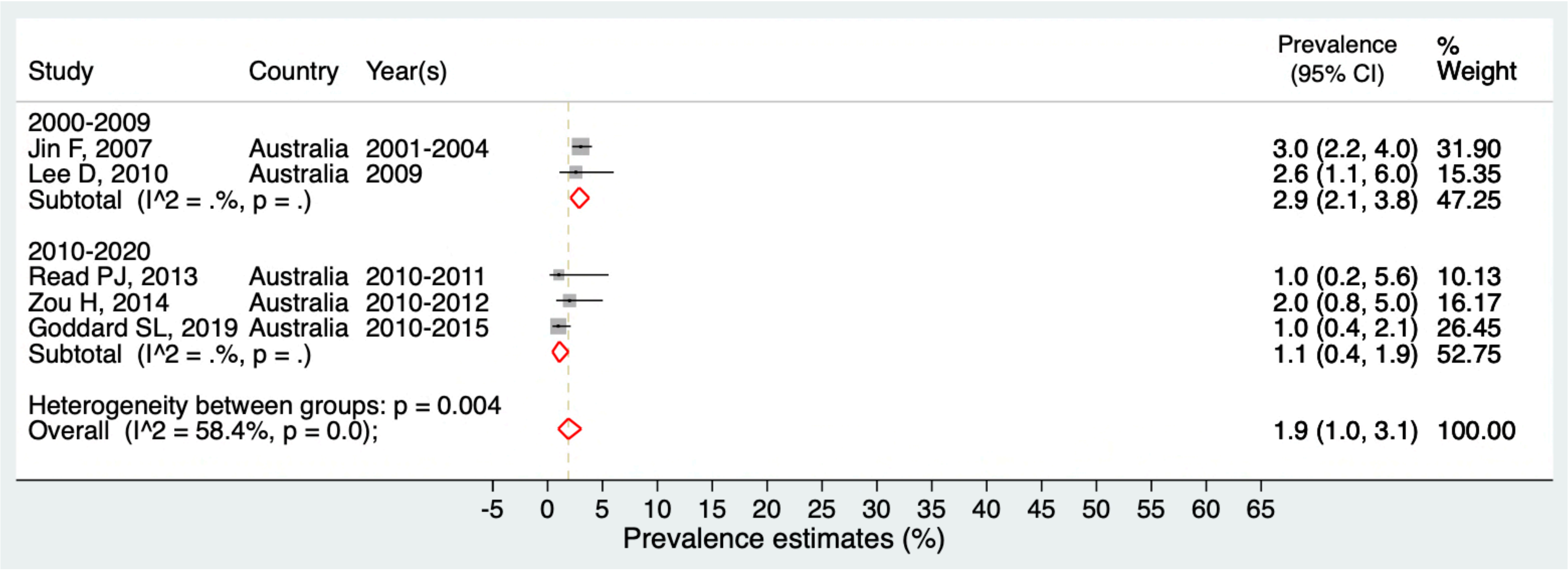

Diamonds indicate pooled mean prevalence estimates; the extremes of the diamonds indicate 95% CIs

Figure S5.2. Central and Southern Asia SDG Region: Pooled syphilis prevalence estimates among MSM between 2000-2020

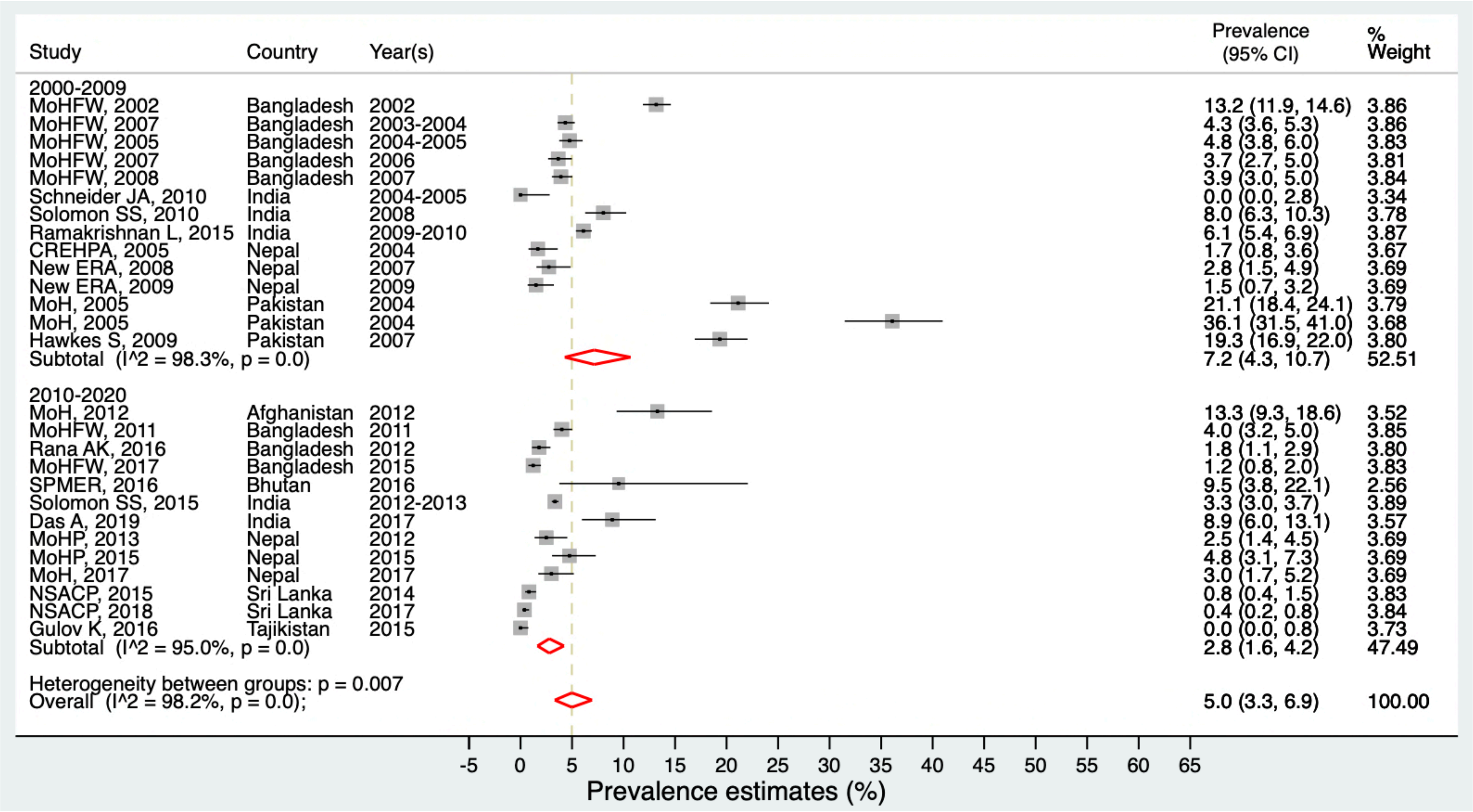

Diamonds indicate pooled mean prevalence estimates; the extremes of the diamonds indicate 95% CIs

Figure S5.3a. Eastern and South-Eastern Asia SDG Region: Pooled syphilis prevalence estimates among MSM between 2000-2009

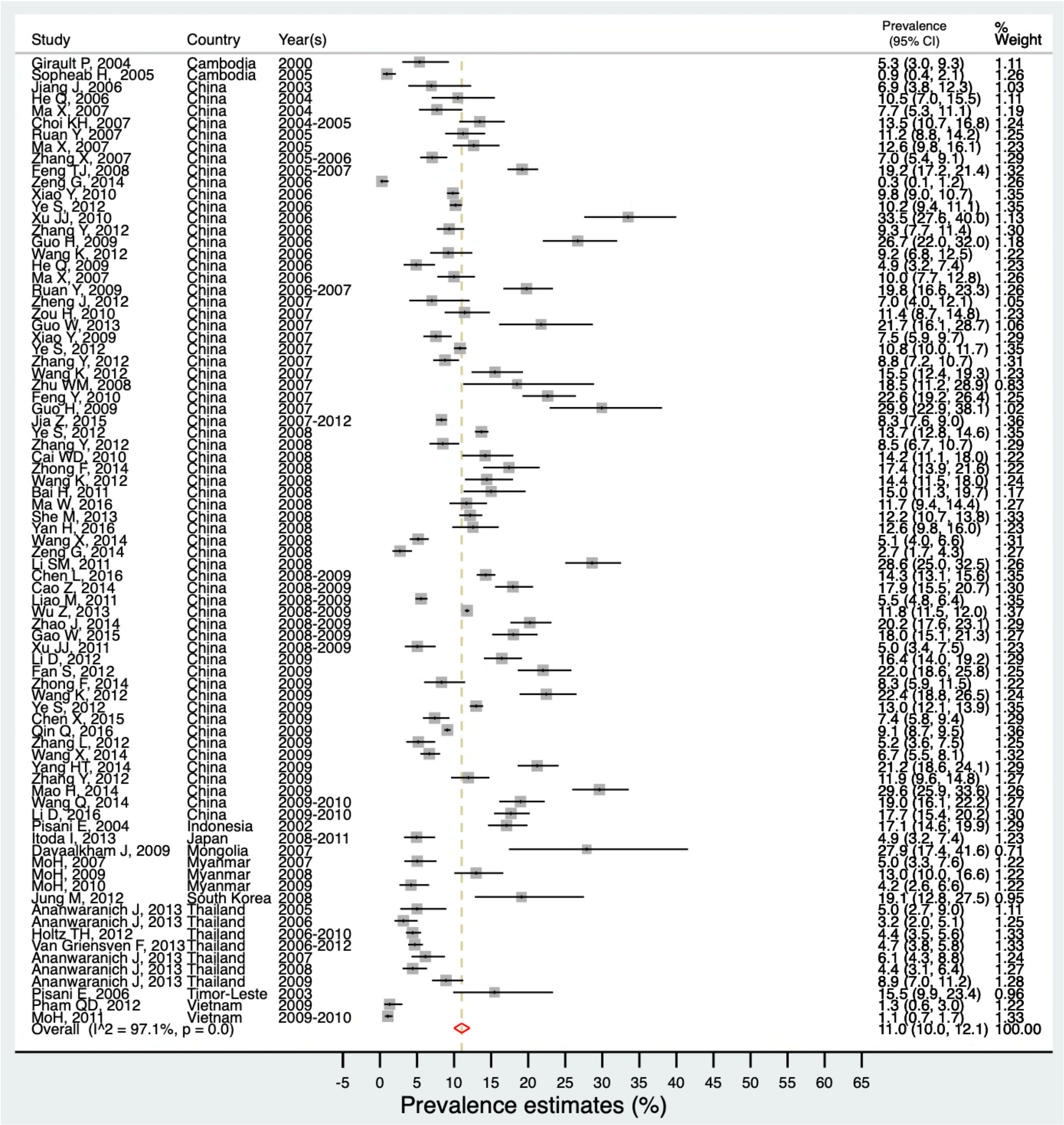

Diamonds indicate pooled mean prevalence estimates; the extremes of the diamonds indicate 95% CIs

Figure S5.3b. Eastern and South-Eastern Asia SDG Region: Pooled syphilis prevalence estimates among MSM between 2010-2020

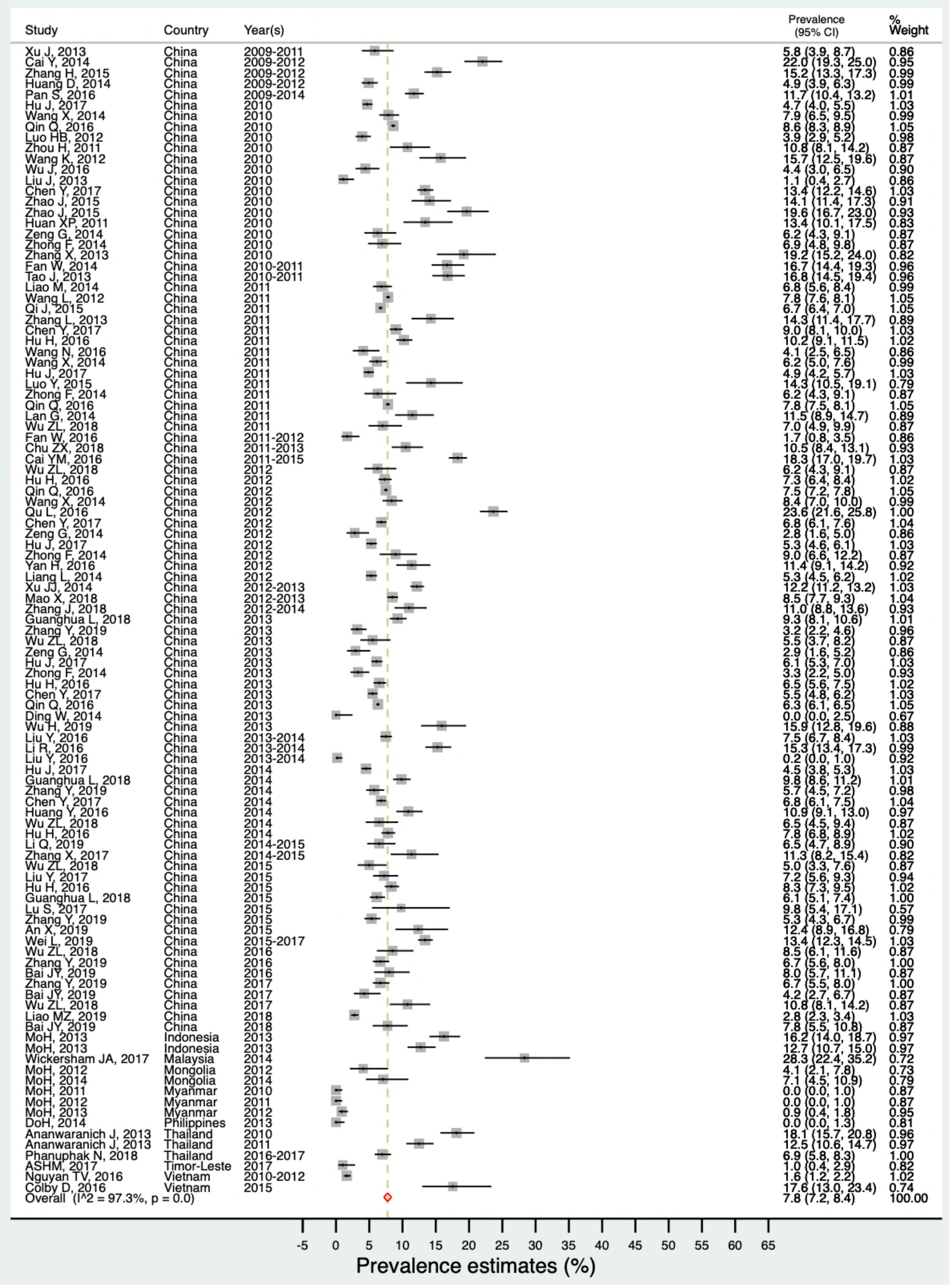

Diamonds indicate pooled mean prevalence estimates; the extremes of the diamonds indicate 95% CIs

Figure S5.4. Europe and Northern America SDG Region: Pooled syphilis prevalence estimates among MSM between 2000-2020

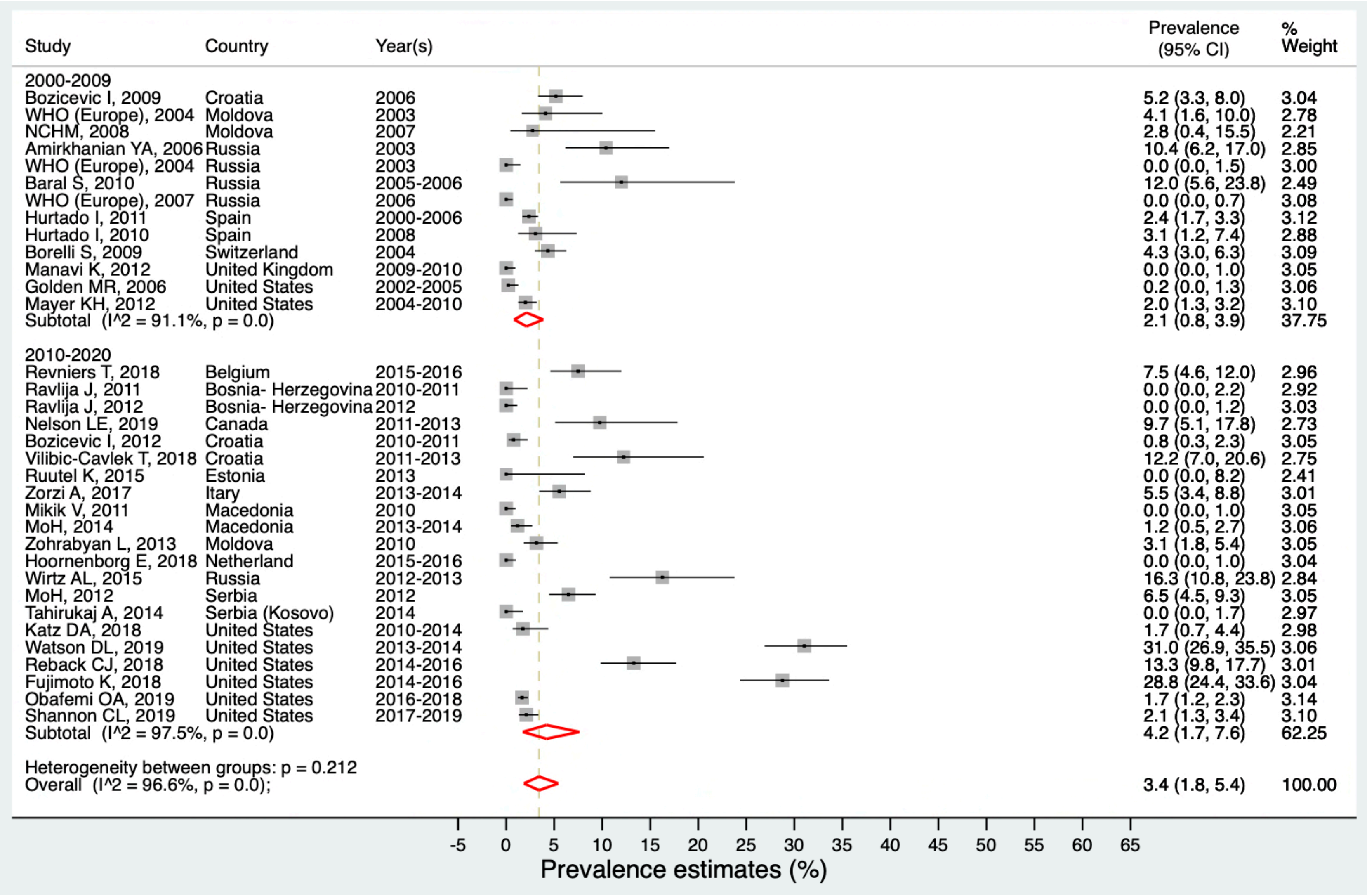

Diamonds indicate pooled mean prevalence estimates; the extremes of the diamonds indicate 95% CIs

Figure S5.5. Latin America and the Caribbean SDG Region: Pooled syphilis prevalence estimates among MSM between 2000-2020

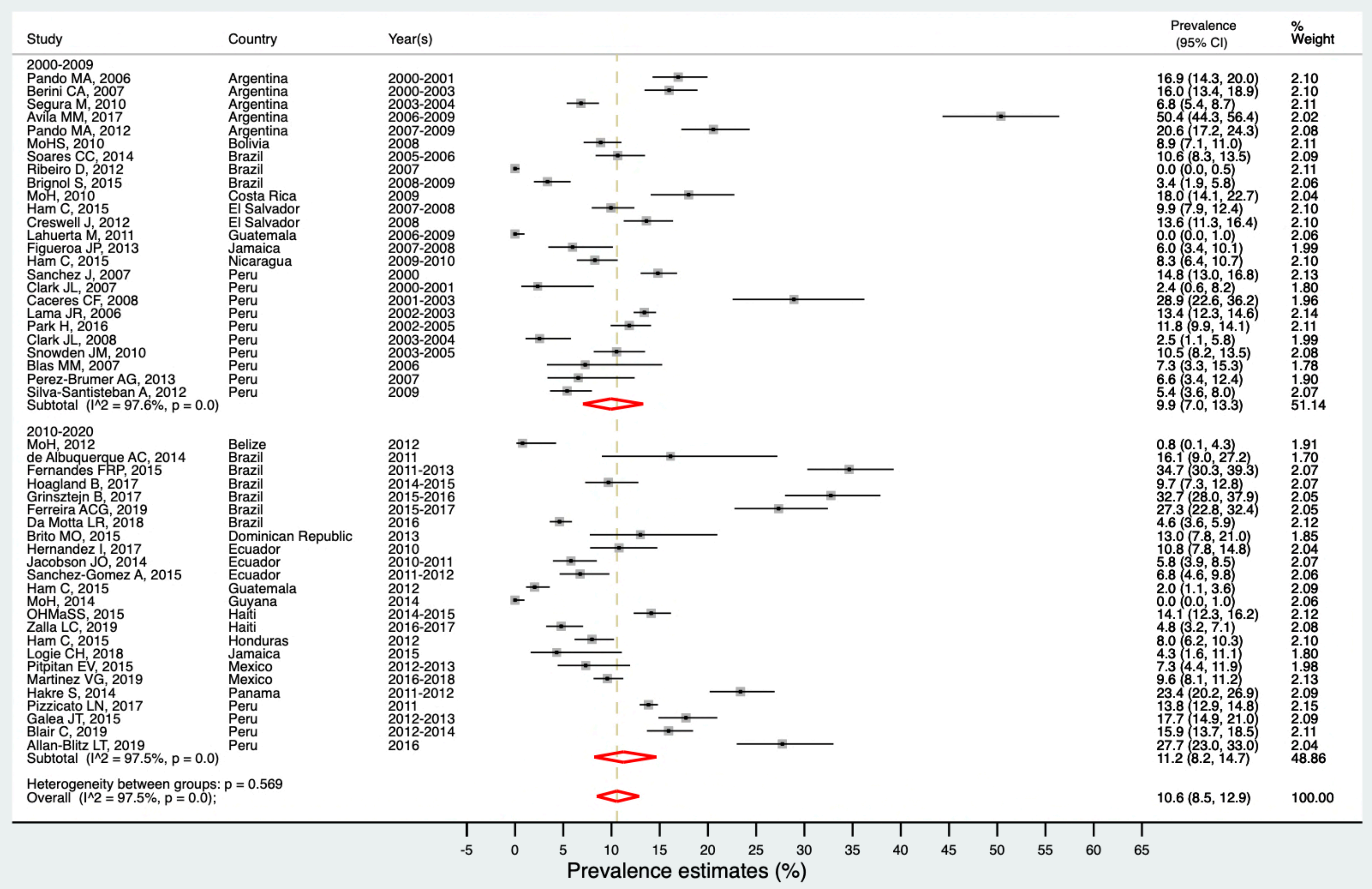

Diamonds indicate pooled mean prevalence estimates; the extremes of the diamonds indicate 95% CIs

Figure S5.6. Northern Africa and Western Asia SDG Region: Pooled syphilis prevalence estimates among MSM between 2000-2020

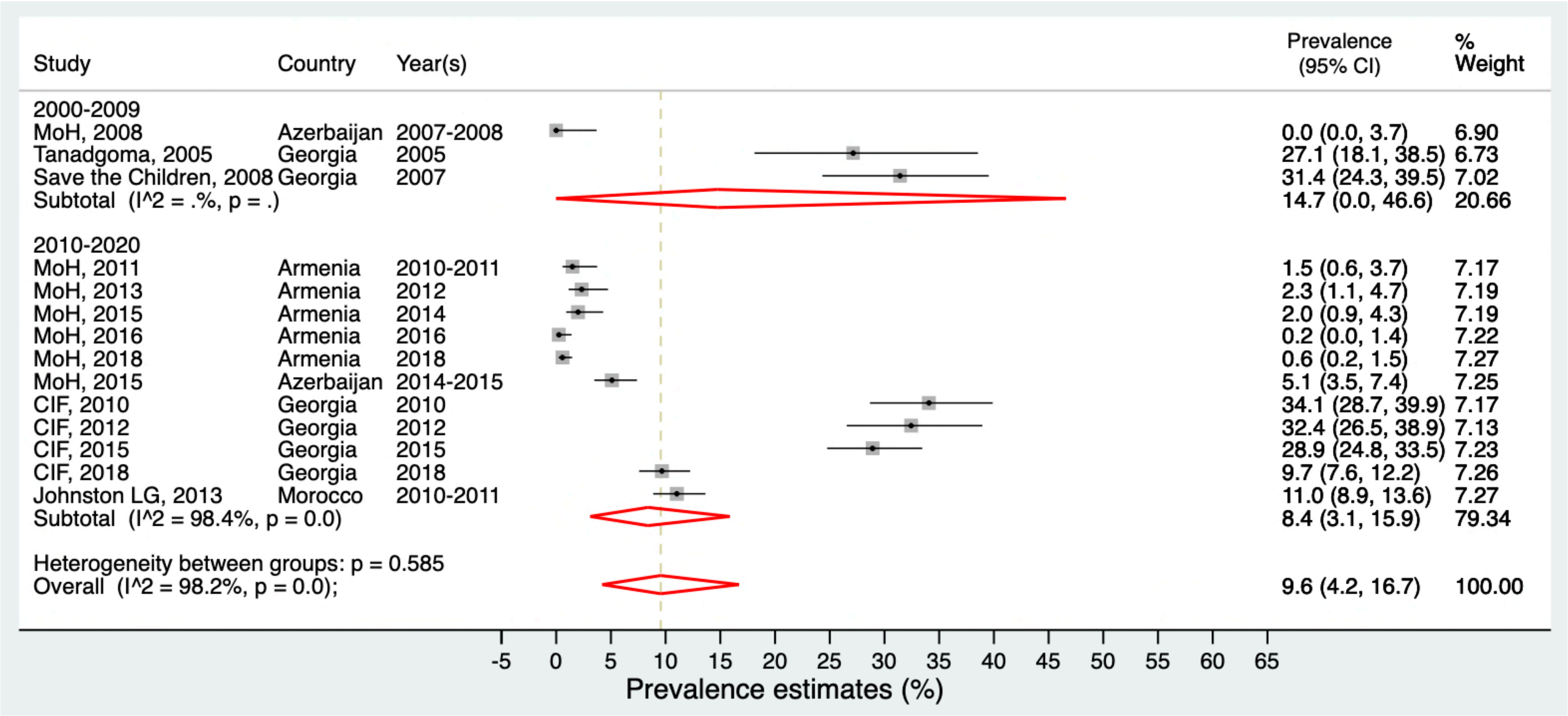

Diamonds indicate pooled mean prevalence estimates; the extremes of the diamonds indicate 95% CIs

Figure S5.7. Oceania (excluding Australia/New Zealand) SDG Region: Pooled syphilis prevalence estimates among MSM between 2000-2020

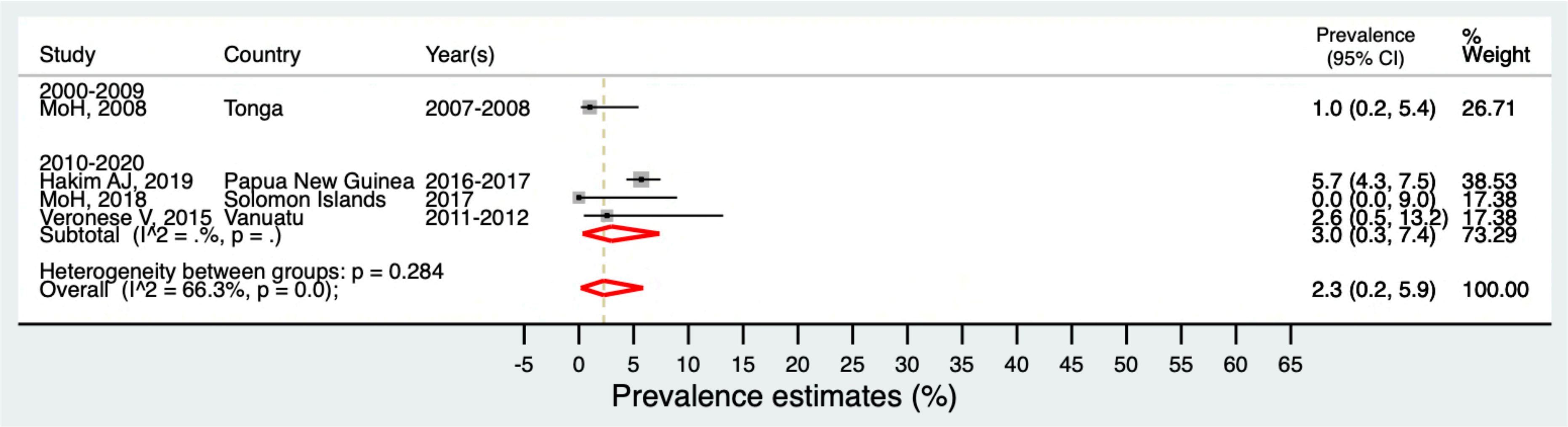

Diamonds indicate pooled mean prevalence estimates; the extremes of the diamonds indicate 95% CIs

Figure S5.8. Sub-Saharan Africa SDG Region: Pooled syphilis prevalence estimates among MSM between 2000-2020

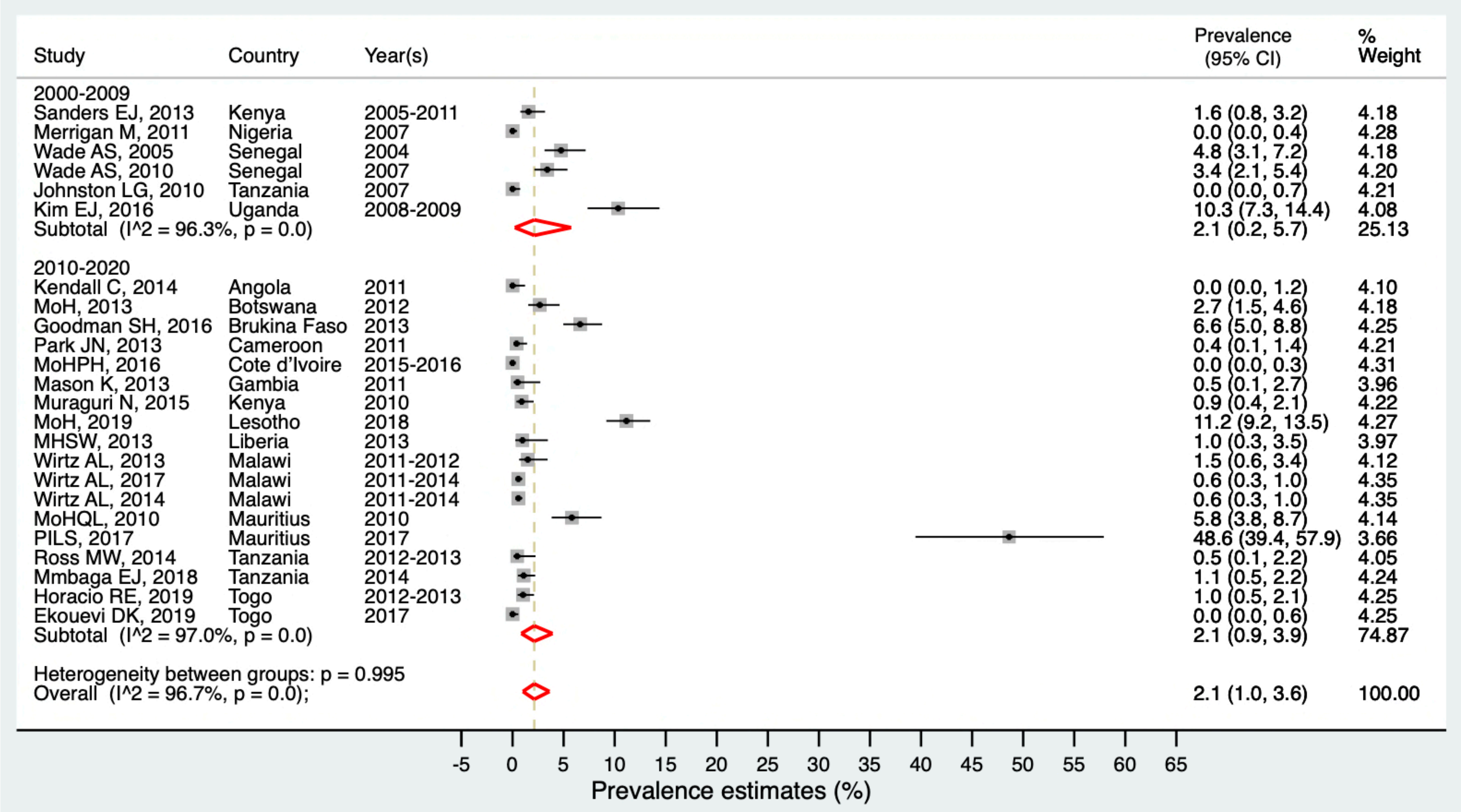

Diamonds indicate pooled mean prevalence estimates; the extremes of the diamonds indicate 95% CIs

Table S1. PRISMA Checklist

Section/topic	#	Checklist item	Reported on page #
**TITLE**			
Title	1	Identify the report as a systematic review, meta-analysis, or both.	1
**ABSTRACT**			
Structured summary	2	Provide a structured summary including, as applicable: background; objectives; data sources; study eligibility criteria, participants, and interventions; study appraisal and synthesis methods; results; limitations; conclusions and implications of key findings; systematic review registration number.	2
**INTRODUCTION**			
Rationale	3	Describe the rationale for the review in the context of what is already known.	5
Objectives	4	Provide an explicit statement of questions being addressed with reference to participants, interventions, comparisons, outcomes, and study design (PICOS).	5
**METHODS**			
Protocol and registration	5	Indicate if a review protocol exists, if and where it can be accessed (e.g., Web address), and, if available, provide registration information including registration number.	6
Eligibility criteria	6	Specify study characteristics (e.g., PICOS, length of follow-up) and report characteristics (e.g., years considered, language, publication status) used as criteria for eligibility, giving rationale.	6
Information sources	7	Describe all information sources (e.g., databases with dates of coverage, contact with study authors to identify additional studies) in the search and date last searched.	6
Search	8	Present full electronic search strategy for at least one database, including any limits used, such that it could be repeated.	6
Study selection	9	State the process for selecting studies (i.e., screening, eligibility, included in systematic review, and, if applicable, included in the meta-analysis).	6-7
Data collection process	10	Describe method of data extraction from reports (e.g., piloted forms, independently, in duplicate) and any processes for obtaining and confirming data from investigators.	7
Data items	11	List and define all variables for which data were sought (e.g., PICOS, funding sources) and any assumptions and simplifications made.	7-8
Risk of bias in individual studies	12	Describe methods used for assessing risk of bias of individual studies (including specification of whether this was done at the study or outcome level), and how this information is to be used in any data synthesis.	8, Supplementary materials p38
Summary measures	13	State the principal summary measures (e.g., risk ratio, difference in means).	7
Synthesis of results	14	Describe the methods of handling data and combining results of studies, if done, including measures of consistency (e.g., I^2) for each meta-analysis.	7

Section/topic	#	Checklist item	Reported on page #
Risk of bias across studies	15	Specify any assessment of risk of bias that may affect the cumulative evidence (e.g., publication bias, selective reporting within studies).	8
Additional analyses	16	Describe methods of additional analyses (e.g., sensitivity or subgroup analyses, meta-regression), if done, indicating which were pre-specified.	7-8
**RESULTS**			
Study selection	17	Give numbers of studies screened, assessed for eligibility, and included in the review, with reasons for exclusions at each stage, ideally with a flow diagram.	8, figure 1
Study characteristics	18	For each study, present characteristics for which data were extracted (e.g., study size, PICOS, follow-up period) and provide the citations.	Supplementary materials p28-34
Risk of bias within studies	19	Present data on risk of bias of each study and, if available, any outcome level assessment (see item 12).	Supplementary materials p28-34
Results of individual studies	20	For all outcomes considered (benefits or harms), present, for each study: (a) simple summary data for each intervention group (b) effect estimates and confidence intervals, ideally with a forest plot.	8, table 1 and 2, and Figure S5.1.-5.8.
Synthesis of results	21	Present results of each meta-analysis done, including confidence intervals and measures of consistency.	8-9
Risk of bias across studies	22	Present results of any assessment of risk of bias across studies (see Item 15).	10-11
Additional analysis	23	Give results of additional analyses, if done (e.g., sensitivity or subgroup analyses, meta-regression [see Item 16]).	9-11, table 1-3
**DISCUSSION**			
Summary of evidence	24	Summarize the main findings including the strength of evidence for each main outcome; consider their relevance to key groups (e.g., healthcare providers, users, and policy makers).	11-13
Limitations	25	Discuss limitations at study and outcome level (e.g., risk of bias), and at review-level (e.g., incomplete retrieval of identified research, reporting bias).	13
Conclusions	26	Provide a general interpretation of the results in the context of other evidence, and implications for future research.	13-14
**FUNDING**			
Funding	27	Describe sources of funding for the systematic review and other support (e.g., supply of data); role of funders for the systematic review.	8

From: Moher D, Liberati A, Tetzlaff J, Altman DG, The PRISMA Group (2009). Preferred Reporting Items for Systematic Reviews and Meta-Analyses: The PRISMA Statement. PLOS Med 6(7): e1000097. doi:10.1371/journal.pmed1000097

Table S2. Sensitivity and specificity of syphilis diagnostic tests

Diagnostic tests	Sensitivity (%)	Specificity (%)	No. of data points	No. of MSM tested
**Non-treponemal test combined with treponemal test**				
RPR plus any Treponemal test (regardless of RPR titer)*	~100	~100	211	501,078
VDRL plus any Treponemal test (regardless of VDRL titer)*	~100	~100	19	10,767
TRUST plus any Treponemal test (regardless of TRUST titer)*	~100	~100	33	56,486
Wasserman reaction assay plus TPHA	~100	~100	1	125
Chembio DPP Syphilis Screen & Confirm Assay	~100	~100	1	860
**Non-treponemal test only**				
RPR only [1-3]	84.6~100	98.3-99.6	3	515
RPR≥1:8	~100	~100	3	712
VDRL only [1-3]	83.8~100	96.1-98.9	6	2,760
**Treponemal test only**				
TPPA [4-6]	92.1~100	95~100	6	2,868
TPHA [5]	86~100	96	8	6,496
EIA/ELISA (overall)[4-6]	85.3~98.5	82.6~98.6	13	5,188
Immunochemiluminescene				
CLIA (LIAISON Treponema screen) [5,6]	95.8~96.9	94.5~99.1	1	374
POCT (Rapid test) (overall)	85.7~98.8	89.7~98.5	7	4,458
Determine Syphilis TP [7]	91.8	98.5	6	2,054
SD Bioine Syphilis 3.0 [8,9]	86.0-98.8	91.4-96.8	5	1,451
Syphilis Health Check [8,10]	85.7-95.3	89.7-98.1	1	788
SD Bioline HIV/Syphilis Duo [11]	95.5	99.9	1	678
Multiplex flow immunoassay [6]	96.9	96.7	1	442
Any combination of two treponemal tests	~100	~100	10	5,827
**Other tests**				
Syphilis antibody/ Treponema antibody/ RPGA†	83.8~100	82.6~100	8	1,923
PCR test for blood specimen [12]	31.2~52.2	83.3~95.7	1	382

Notes:
1. For studies that reported more than one data point for the same study population, we used prevalence data based on qualitative diagnostic methods that were based on both non-treponemal and treponemal tests to produce corrected point prevalence estimates. One exception was a study that had data based on qualitative and quantitative non-treponemal tests; we used quantitative non-treponemal test results because they are considered to be more accurate.
2. If there was a range for either the sensitivity or specificity of a specific biological assay, we used the midpoint to calculate the corrected point estimate.

*Including TPPA, TPHA, FTA-ABS, MHA-TP, EIA, ELISA, POCT (rapid test).
†Considered as the whole range of any non-treponemal and treponemal test because the definition of syphilis antibody or treponemal antibody was not clearly described and no sensitivity and specificity data of RPGA was obtained.

CLIA: chemiluminescence immunoassay, EIA: enzyme immunoassay, ELISA: enzyme linked immunosorbent assay, FTA-ABS: fluorescent treponemal antibody absorption, MHA-TP: microhemagglutination assay for Treponema pallidum, POCT: point of care test, RPGA: reaction of passive hemagglutination, RPR: rapid plasma reagin, TPHA: treponema pallidum hemagglutination test, TPPA: treponema pallidum particle agglutination test, TRUST: toluidine red unheated serum test, VDRL: venereal disease research laboratory.

Table S3. Pooled syphilis prevalence estimates among MSM in regions of the World Health Organization between 2000-2020

WHO Regions	No. of MSM tested positive	No. of MSM tested (% of total)	Uncorrected pooled prevalence estimates (95%CI)	Corrected pooled prevalence estimates (95%CI)	Median no. of positive diagnoses	Range of study sample size	No. of countries	No. of point prevalence data (% of total)	Heterogeneity I^2 (%)*
**2000-2020**									
Americas	4,567	38,104 (6·3)	11·0 (8·9-13·2)	10·0 (7·9-12·3)	54	62-5,101	19	58 (16·8)	98·0
Africa	573	16,054 (2·6)	2·9 (1·6-4·6)	2·1 (1·0-3·6)	7	109-2,442	16	24 (7·0)	96·7
Eastern Mediterranean	592	2,958 (0·5)	20·4 (13·7-28·1)	19·6 (12·8-27·4)	140	207-915	3	5 (1·4)	95·9
Europe	874	12,635 (2·1)	6·6 (4·3-9·4)	4·1 (2·2-6·5)	11	31-1,387	17	39 (11·3)	97·0
South-East Asia	2,763	49,700 (8·2)	5·4 (4·3-6·7)	4·7 (3·6-6·0)	27	42-11,997	9	43 (12·5)	97·3
Western Pacific	42,698	486,781 (80·3)	9·5 (9·0-10·1)	9·0 (8·5-9·6)	65.5	39-47,231	13	176 (51·0)	97·6
Total	52,067	606,232 (100)	8·4 (7·9-8·9)	7·5 (7·0-8·0)	48	31-47,231	77	345 (100)	98·1
**2010-2020**									
Americas	2,654	21,200 (5·0)	12·2 (9·1-15·7)	11·0 (7·9-14·4)	49	62-5,101	14	31 (15·5)	98·2
Africa	496	12,985 (3·1)	3·1 (1·6-5·1)	2·1 (0·9-3·9)	7	109-2,442	13	18 (9·0)	97·0
Eastern Mediterranean	109	869 (0·2)	12·4 (10·2-14·7)	11·5 (9·5-13·7)	54.5	207-662	2	2 (1·0)	-
Europe	628	8,321 (2·0)	5·9 (3·0-9·5)	3·9 (1·6-7·2)	9	43-700	14	25 (12·5)	97·6
South-East Asia	1,323	27,569 (6·6)	4·9 (3·1-7·1)	3·9 (2·3-6·1)	20	42-11,997	9	20 (10·0)	98·1
Western Pacific	27,156	349,411 (83·1)	8·0 (7·5-8·6)	7·7 (7·1-8·2)	66·5	39-42,680	9	104 (52·0)	97·0
Sub-total	32,366	420,355 (100)	7·5 (6·9-8·0)	6·6 (6·0-7·2)	43	39-42,680	61	200 (100)	98·0
**2000-2009**									
Americas	1,913	16,904 (9·1)	9·6 (7·0-12·6)	9·0 (6·1-12·2)	54	78-3,280	10	27 (18·6)	97·9
Africa	77	3,069 (1·7)	2·4 (0·4-5·8)	2·1 (0·2-5·7)	12	290-879	5	6 (4·1)	96·3
Eastern Mediterranean	483	2,089 (1·1)	25·0 (16·9-34·2)	25·0 (16·9-34·2)	166	388-915	1	3 (2·1)	-
Europe	246	4,314 (2·3)	8·1 (4·7-12·3)	4·4 (1·7-8·2)	12·5	31-1,387	8	14 (9·7)	95·4
South-East Asia	1,440	22,131 (11·9)	5·9 (4·6-7·5)	5·5 (4·1-7·0)	38	110-3,739	7	23 (15·9)	95·0
Western Pacific	15,542	137,370 (73·9)	12·0 (10·9-13·1)	11·3 (10.2-12·5)	64.5	50-47,231	8	72 (49·7)	97·2
Sub-total	19,701	185,877 (100)	9·8 (8·9-10·8)	8·9 (8·0-9·9)	54	31-47,231	39	145 (100)	97.8

*Heterogeneity between subgroups in all subgroup analyses had a P value <0.001

Table S4. Pooled syphilis prevalence estimates among MSM in regions of the World Health Organization between 2000-2020 in five-year intervals

WHO Regions	No. of MSM tested positive	No. of MSM tested (% of total)	Uncorrected pooled prevalence estimates (95%CI)	Corrected pooled prevalence estimates (95%CI)	Median no. of positive diagnoses	Range of study sample size	No. of countries	No. of point prevalence data (% of total)	Heterogeneity I^2 (%)*
**2015-2020**									
Americas	841	7,950 (19·7)	14·5 (8·2-22·2)	12·0 (6·2-19·3)	62	83-2,296	6	11 (22·4)	98·7
Africa	151	2,900 (7·2)	8·4 (0·5-23·8)	7·6 (0·1-24·1)	29·5	109-1,288	4	4 (8·2)	99·3
Eastern Mediterranean	-	-	-	-	-	-	-	-	-
Europe	235	3,212 (8·0)	5·3 (0·7-13·4)	3·6 (0·1-11·2)	11	200-700	5	7 (14·3)	98·6
South-East Asia	213	5,905 (14·6)	4·2 (1·7-7·6)	3·4 (1·2-6·5)	17·5	42-1,626	7	8 (16·3)	96·1
Western Pacific	1,534	20,390 (50·5)	7·3 (5·7-9·2)	7·3 (5·7-9·2)	43	39-3,613	4	19 (38·8)	95·3
Sub-total	2,974	40,357 (100)	7·9 (6·0-9·9)	6·9 (5·1-8·9)	38	39-3,613	26	49 (100)	98·2
**2010-2014**									
Americas	1,813	13,250 (3·5)	11·0 (7·8-14·7)	10·4 (7·1-14·2)	29	62-5,101	13	20 (13·2)	97·3
Africa	345	10,085 (2·7)	2·2 (1·3-3·4)	1·3 (0·6-2·1)	7	205-2,442	11	14 (9·3)	89·9
Eastern Mediterranean	109	869 (0·2)	12·4 (10·3-14·7)	11·5 (9·5-13·7)	54·5	207-662	2	2 (1·3)	-
Europe	393	5,109 (1·3)	6·1 (2·9-10·5)	4·1 (1·5-7·9)	8	43-500	11	18 (11·9)	96·9
South-East Asia	1,110	21,664 (5·7)	5·4 (2·9-8·5)	4·3 (2·0-7·4)	48·5	400-11,997	7	12 (7·9)	98·6
Western Pacific	25,622	329,021 (86·6)	8·2 (7·6-8·8)	7·7 (7·2-8·4)	71	39-42,680	7	85 (56·3)	97·2
Sub-total	29,392	379,998 (100)	7·4 (6·8-8·0)	6·5 (5·9-7·1)	44	39-42,680	51	151 (100)	98.0
**2005-2009**									
Americas	757	7,727 (4·8)	9·4 (5·5-14·1)	8·4 (4·3-13·6)	43	78-850	10	16 (14·2)	98·2
Africa	56	2,627 (1·6)	2·0 (0·1-5·7)	1·7 (0-5·6)	7	290-879	5	5 (4·4)	96·5
Eastern Mediterranean	177	915 (0·6)	19·3 (16·9-22·0)	19·3 (16·9-22·0)	177	915-915	1	1 (0·9)	-
Europe	127	1,840 (1·1)	8·8 (3·2-16·6)	5·4 (0·8-13·3)	8	31-558	7	9 (8·0)	96·7
South-East Asia	801	14,724 (9·1)	5·3 (4·3-6·4)	5·0 (4·1-6·1)	33	200-3,739	5	16 (14·2)	85·8
Western Pacific	15,369	134,624 (82·9)	12·4 (11·3-13·6)	11·7 (10·6-12·9)	68	50-47,231	8	66 (58·4)	97·3
Sub-total	17,287	162,457 (100)	10·0 (9·0-11·0)	9·1 (8·1-10·2)	54	31-47,231	36	113 (100)	97.8
**2000-2004**									
Americas	1,156	9,177 (39·2)	10·1 (6·7-14·0)	9·9 (6·6-13·8)	60	85-3,280	3	11 (34·4)	96·6
Africa	21	442 (1·9)	4·8 (3·1-7·2)	4·8 (3·1-7·2)	21	442-442	1	1 (3·1)	-
Eastern Mediterranean	306	1,174 (5·0)	25·8 (23·3-28·3)	25·8 (23·3-28·3)	153	388-786	1	2 (6·3)	-
Europe	119	2,474 (10·6)	7·1 (3·5-11·8)	3·1 (1·0-6·3)	21	99-1,387	4	5 (15·6)	89·7
South-East Asia	639	7,407 (31·6)	7·6 (3·9-12·4)	6·5 (2·9-11·3)	65	110-2,399	5	7 (21·9)	97·8
Western Pacific	173	2,746 (11·7)	7·4 (3·8-12·1)	7·4 (3·8-12·1)	23	144-1,396	3	6 (18·8)	92·7
Sub-total	2,414	23,420 (100)	9·2 (7·0-11·8)	8·1 (5·9-10·6)	41	85-3,280	17	32 (100)	97·6

*Heterogeneity between subgroups in all subgroup analyses except for the period 2015-2020 had a P value <0.001, while the period 2015-2020 had a P value of 0·07.

Table S5. Sensitivity and specificity of HIV diagnostic tests

Diagnostic tests	Sensitivity (%)	Specificity (%)	No. of data points	No. of MSM tested
≥ 2 HIV diagnostics	~100	~100	305	586,139
NAAT/ PCR	100	100	1	382
EIA/ ELISA (overall) [13]	100	99.4~100	7	2,677
Genscreen ULTRA HIV Ag-Ab [14]	100	98.7~100	1	397
Rapid test (overall) [13]	97.6~100	97.6~100	1	423
Abbott Determine HIV-1/2[13]	100	99.4	1	171
Alere Determine HIV-1/2 Ag/Ab rapid test [13]	100	98.9	1	788
Hexagon HIV [15]	99.0	99.0	1	400
Murex HIV Ag/Ab combination [13]	100	99.3	1	300
Uni-Gold HIV-1/2 [13]	99.8~100	99.9~100	1	2,296
HIV antibody/ No information about HIV diagnostic	97.6~100	97.6~100	11	6,145

EIA: enzyme immunoassay, ELISA: enzyme-linked immunosorbent assay, NAAT: nucleic acid amplification techniques, PCR: polymerase chain reaction

Table S6. Pooled syphilis prevalence estimates among MSM in regions of the Sustainable Development Goals between 2000-2020 in five-year intervals

SDG regions	No. of MSM tested positive	No. of MSM tested (% of total)	Uncorrected pooled prevalence estimates (95%CI)	Corrected pooled prevalence estimates (95%CI)	Median no. of positive diagnoses	Range of study sample size	No. of countries	No. of point prevalence data (% of total)	Heterogeneity I^2 (%)*
**2015-2020**									
Australia and New Zealand	-	-	-	-	-	-	0	0 (0)	-
Central and Southern Asia	90	4,471 (11·1)	3·0 (1·2-5·6)	2·4 (0·7-5·0)	12	42-1,573	6	7 (14·3)	94·1
Eastern and South-Eastern Asia	1,619	21,427 (53·1)	7·7 (6·1-9·5)	7·3 (5·6-9·1)	43	102-3,613	4	19 (38·8)	95·5
Europe and Northern America	264	4,309 (10·7)	8·3 (2·5-17·0)	6·2 (1·2-14·4)	38	200-2,296	3	6 (12·2)	98·5
Latin America and the Caribbean	598	4,215 (10·4)	16·8 (9·3-26·0)	14·0 (6·8-23·2)	84	83-1,398	5	7 (14·3)	98·2
Northern Africa and Western Asia	203	2,136 (5·3)	6·9 (0-23·5)	6·3 (0·1-21·0)	32	400-700	2	4 (8·2)	99·1
Oceania (excluding Australia and New Zealand)	49	899 (2·2)	4·8 (3·4-6·4)	4·8 (3·4-6·4)	24·5	39-860	2	2 (4·1)	-
Sub-Saharan Africa	151	2,900 (7·2)	8·4 (0·5-23·8)	7·6 (0·1-24·1)	29·5	109-1,288	4	4 (8·2)	99·3
Total	2,974	40,357 (100)	7·9 (6·0-9·9)	6·9 (5·1-8·9)	38	39-3,613	26	49 (100)	98·2
**2010-2014**									
Australia and New Zealand	11	915 (0·2)	1·1 (0·4-1·9)	1·1 (0·4-1·9)	4	98-617	1	3 (2·0)	-
Central and Southern Asia	545	16,507 (4·3)	3·6 (2·0-5·6)	3·3 (1·9-5·0)	26	207-11,997	5	6 (4·0)	93·8
Eastern and South-Eastern Asia	26,211	333,431 (87·7)	8·5 (7·9-9·1)	7·9 (7·3-8·5)	78	151-42,680	8	88 (58·3)	97·5
Europe and Northern America	306	4,010 (1·1)	5·8 (2·2-10·7)	3·5 (0·8-7·8)	9	43-442	10	15 (9·9)	97·1
Latin America and the Caribbean	1,651	12,492 (3·3)	10·5 (7·4-14·0)	10·2 (6·9-14·0)	33	62-5,101	11	17 (11·3)	97·2
Northern Africa and Western Asia	322	2,519 (0·7)	11·1 (4·0-21·1)	9·7 (3·1-19·4)	68	216-662	4	7 (4·6)	98·0
Oceania (excluding Australia and New Zealand)	1	39 (0·01)	2·6 (0·5-13·2)	2·6 (0·5-13·2)	1	39-39	1	1 (0·7)	-
Sub-Saharan Africa	345	10,085 (2·7)	2·2 (1·3-3·4)	1·3 (0·6-2·1)	7	205-2,442	11	14 (9·3)	89·9
Sub-total	29,392	379,998 (100)	7·4 (6·8-8·0)	6·5 (5·9-7·1)	44	39-42,680	51	151 (100)	98.0
**2005-2009**									
Australia and New Zealand	7	183 (0·1)	3·8 (1·9-7·7)	2·6 (1·1-6·0)	7	183-183	1	1 (0·9)	-
Central and Southern Asia	576	8,694 (5·4)	5·7 (2·9-9·3)	5·7 (2·9-9·3)	58	400-3,739	4	7 (6·2)	97·3
Eastern and South-Eastern Asia	15,763	141,286 (87·0)	11·7 (10·7-12·8)	11·1 (10·0-12·2)	65	50-47,231	8	74 (65·5)	97·3
Europe and Northern America	73	2,380 (1·5)	3·5 (1·2-6·7)	1·9 (0·2-4·9)	6	31-850	6	7 (6·2)	91·6
Latin America and the Caribbean	740	6,877 (4·2)	10·1 (6·0-15·0)	9·0 (4·5-14·6)	54	78-833	9	15 (13·3)	98·1
Northern Africa and Western Asia	71	310 (0·2)	21·1 (7·7-38·7)	14·7 (0-46·6)	19	70-140	2	3 (2·7)	-
Oceania (excluding Australia and New Zealand)	1	100 (0·1)	1·0 (0·2-5·4)	1·0 (0·2-5·4)	1	100-100	1	1 (0·9)	-
Sub-Saharan Africa	56	2,627 (1·6)	2·0 (0·1-5·7)	1·7 (0-5·6)	7	290-879	5	5 (4·4)	96·5
Sub-total	17,287	162,457 (100)	10·0 (9·0-11·0)	9·1 (8·1-10·2)	54	31-47231	36	113 (100)	97.8
**2000-2004**									
Australia and New Zealand	42	1,396 (6·0)	3·0 (2·2-4·0)	3·0 (2·2-4·0)	42	1,396-1,396	1	1 (3·1)	-
Central and Southern Asia	797	7,704 (32·9)	10·1 (4·6-17·4)	8·8 (3·6-16·0)	99	132-2,399	4	7 (21·9)	98·8
Eastern and South-Eastern Asia	279	2,227 (9·5)	10·7 (7·4-14·5)	10·7 (7·4-14·5)	21	110-767	4	7 (21·9)	85·1
Europe and Northern America	120	2,919 (12·5)	5·4 (2·2-9·7)	2·4 (0·7-5·1)	17	99-1,387	5	6 (18·8)	91·3
Latin America and the Caribbean	1,155	8,732 (37·3)	11·9 (9·2-14·9)	11·7 (9·0-14·7)	84·5	85-3,280	2	10 (31·3)	93·1
Northern Africa and Western Asia	-	-	-	-	-	-	0	0 (0)	-
Oceania (excluding Australia and New Zealand)	-	-	-	-	-	-	0	0 (0)	-
Sub-Saharan Africa	21	442 (1·9)	4·8 (3·1-7·2)	4·8 (3·1-7·2)	21	442-442	1	1 (3·1)	-
Sub-total	2,414	23,420 (100)	9·2 (7·0-11·8)	8·1 (5·9-10·6)	41	85-3,280	17	32 (100)	97·6

*Heterogeneity between subgroups in all sub-group analyses had a *P* value <0·05

Table S7. Pooled syphilis prevalence estimates among MSM by country between 2000-2020

Country	No. of point prevalence data	Corrected pooled Prevalence estimates	95% CI
Afghanistan	1	13·3	9·3-18·6
Angola	1	0	0-1·2
Argentina	5	20·5	10·6-32·7
Armenia	5	1·1	0·4-2·1
Australia	5	1·9	1·0-3·1
Azerbaijan	2	3·7	2·2-5·4
Bangladesh	8	4·2	2·1-6·9
Belgium	1	7·5	4·6-12·0
Belize	1	0·8	0·1-4·3
Bhutan	1	9·5	3·8-22·1
Bolivia	1	8·9	7·1-11·0
Bosnia-Herzegovina	2	0	0-0·4
Botswana	1	2·7	1·5-4·6
Brazil	9	12·5	4·7-23·3
Burkina Faso	1	6·6	5·0-8·8
Cambodia	2	1·8	0·9-2·9
Cameroon	1	0·4	0·1-1·4
Canada	1	9·7	5·1-17·8
China	153	9·7	9·2-10·3
Costa Rica	1	18·0	14·1-22·7
Cote d'Ivoire	1	0	0-0·3
Croatia	3	4·7	0·6-12·1
Dominican Republic	1	13·0	7·8-21·0
Ecuador	3	7·6	5·0-10·6
El Salvador	2	11·7	10·1-13·4
Estonia	1	0	0-8·2
Gambia	1	0·5	0·1-2·7
Georgia	6	26·5	16·4-38·1
Guatemala	2	0·8	0·3-1·5
Guyana	1	0	0-1·0
Haiti	2	11·0	9·6-12·6
Honduras	1	8·0	6·2-10·3
India	5	4·7	2·7-7·3
Indonesia	3	15·3	12·7-18·0
Italy	1	5·5	3·4-8·8
Jamaica	2	5·4	3·0-8·4
Japan	1	4·9	3·3-7·4
Kenya	2	1·2	0·6-1·9
Lesotho	1	11·2	9·2-13·5

Country	No. of point prevalence data	Corrected pooled Prevalence estimates	95% CI
Liberia	1	1·0	0·3-3·5
Macedonia	2	0·4	0-1·0
Malawi	3	0·6	0·3-1·0
Malaysia	1	28·3	22·4-35·2
Mauritius	2	12·7	9·8-15·9
Mexico	2	9·2	7·8-10·7
Moldova	3	2·9	1·5-4·6
Mongolia	3	10·3	2·9-21·4
Morocco	1	11·0	8·9-13·6
Myanmar	6	2·4	0·2-6·6
Nepal	6	2·6	1·8-3·6
Netherlands	1	0	0-1·0
Nicaragua	1	8·3	6·4-10·7
Nigeria	1	0	0-0·4
Pakistan	3	25·0	16·9-34·2
Panama	1	23·4	20·2-26·9
Papua New Guinea	1	5·7	4·3-7·5
Peru	14	12·3	10·0-14·7
Philippines	1	0	0-1·3
Republic of Korea	1	19·1	12·8-27·5
Russia	5	4·8	0·1-14·7
Senegal	2	4·0	2·8-5·4
Serbia	2	3·0	1·8-4·6
Solomon Islands	1	0	0-9·0
Spain	2	2·3	1·6-3·2
Sri Lanka	2	0·6	0·3-0·9
Switzerland	1	4·3	3·0-6·3
Tajikistan	1	0	0-0·8
Tanzania	3	0·4	0-1·4
Thailand	10	7·0	4·7-9·7
Timor-Leste	2	3·2	1·6-5·1
Togo	2	0·3	0·1-0·7
Tonga	1	1·0	0·2-5·4
Uganda	1	10·3	7·3-14·4
United Kingdom	1	0	0-1·0
United States	8	6·9	1·8-14·9
Vanuatu	1	2·6	0·5-13·2
Viet Nam	4	3·5	1·0-7·3

Table S8. Individual studies identified by systematic review containing syphilis prevalence estimates among MSM

Study	Country	Study year(s)	Income level	Study design	Sampling method*	Diagnostic method	HIV prevalence (crude)	No. syphilis positive	No. syphilis tested	Syphilis prevalence (crude)†	Axis score
**Australia and New Zealand**											
Jin, 2007 [16]	Australia	2001-2004	High	Cross-sectional	Convenience	TPPA+FTA-ABS	0.0%	42	1396	3.0%	12
Lee, 2010 [17]	Australia	2009	High	Cross-sectional	Convenience	POCT	-	7	183	3.8%	9
Read, 2013 [18]	Australia	2010-2011	High	Cross-sectional	Convenience	RPR+TPPA+FTA-ABS	-	1	98	1.0%	13
Zou, 2014 [19]	Australia	2010-2012	High	Cross-sectional	Convenience	RPR+EIA	0.0%	4	200	2.0%	11
Goddard, 2019 [20]	Australia	2010-2015	High	Cohort	Convenience	RPR≥1:4+TPPA±FTA-ABS	35.8%	6	617	1.0%	12
**Central and Southern Asia**											
MOH, 2012 [21]	Afghanistan	2012	Low	Cross-sectional	RDS	POCT	0.5%	36	207	17.4%	15
MOH, 2002 [22]	Bangladesh	2002	Low	Cross-sectional	Convenience	RPR+TPHA	0.2%	316	2399	13.2%	14
MOH, 2007 [23]	Bangladesh	2003-2004	Low	Cross-sectional	Convenience	RPR≥1:8+TPHA/TPPA	0.1%	99	2276	4.3%	14
MOH, 2005 [24]	Bangladesh	2004-2005	Low	Cross-sectional	Convenience	RPR≥1:8+TPPA	0.4%	65	1365	4.8%	14
MOH, 2007 [25]	Bangladesh	2006	Low	Cross-sectional	Convenience	RPR≥1:8+TPPA	0.5%	38	1038	3.7%	13
MOH, 2008 [26]	Bangladesh	2007	Low	Cross-sectional	Convenience	RPR≥1:8+TPPA	0.2%	58	1481	3.9%	14
MOH, 2011 [27]	Bangladesh	2011	Low	Cross-sectional	Convenience	RPR≥1:8+TPPA	0.3%	72	1797	4.0%	14
Rana, 2016¶ [28]	Bangladesh	2012	Low	Cross-sectional	VCT	RPR≥1:8+TPPA	0.8%	16	889	1.8%	12
MOH, 2017 [29]	Bangladesh	2015	Lower-middle	Cross-sectional	Convenience	RPR≥1:8+TPPA	0.6%	16	1307	1.2%	14
SPMER, 2016 [30]	Bhutan	2016	Lower-middle	Cross-sectional	RDS	RPR+TPPA	0.0%	4	42	9.5%	13
Schneider, 2010 [31]	India	2004-2005	Low	Cross-sectional	Probability	ELISA	7.6%	5	132	3.8%	15
Solomon, 2010 [32]	India	2008	Lower-middle	Cross-sectional	RDS	RPR+TPPA	9.0%	58	721	8.0%	13
Ramakrishnan, 2015 [33]	India	2009-2010	Lower-middle	Cross-sectional	TLS	RPR+TPHA	12.5%	228	3739	6.1%	14
Solomon, 2015 [34]	India	2012-2013	Lower-middle	Cross-sectional	RDS	RPR+TPHA	9.6%	401	11997	3.3%	11
Das, 2019 [35]	India	2017	Lower-middle	Cross-sectional	Snowball	RPR≥1:8	3.2%	22	247	8.9%	14
CREHPA, 2005 [36]	Nepal	2004	Low	Cross-sectional	RDS	RPR≥1:8+TPPA	3.9%	6	358	1.7%	13
New ERA, 2008 [37]	Nepal	2007	Low	Cross-sectional	RDS	RPR+TPHA	3.3%	11	400	2.8%	15
New ERA, 2009 [38]	Nepal	2009	Low	Cross-sectional	RDS	RPR≥1:8+TPHA	3.8%	6	400	1.5%	12
MOH, 2013 [39]	Nepal	2012	Low	Cross-sectional	RDS	RPR+TPPA	6.3%	10	400	2.5%	17
MOH, 2015 [40]	Nepal	2015	Low	Cross-sectional	RDS	RPR+TPPA	5.3%	19	400	4.8%	15
MOH, 2017 [41]	Nepal	2017	Low	Cross-sectional	RDS	RPR+TPPA	6.3%	12	400	3.0%	17
MOH, 2005¶‡ [42]	Pakistan	2004	Low	Cross-sectional	Snowball	RPR+TPHA	2.2%	166	786	21.1%	14
MOH, 2005¶ [42]	Pakistan	2004	Low	Cross-sectional	Probability	RPR+TPHA	1.0%	140	388	36.1%	14
Hawkes, 2009¶‡ [43]	Pakistan	2007	Low	Cross-sectional	RDS	RPR+TPHA	0.8%	177	915	19.3%	13
NSACP, 2015 [44]	Sri Lanka	2014	Lower-middle	Cross-sectional	RDS	VDRL≥16+POCT	0.6%	10	1217	0.8%	15
NSACP, 2018 [45]	Sri Lanka	2017	Lower-middle	Cross-sectional	RDS	VDRL+TPPA	0.3%	6	1573	0.4%	15
Gulov, 2016 [46]	Tajikistan	2015	Lower-middle	Cross-sectional	Convenience	Syphilis antibody	2.6%	11	502	2.2%	12
**Eastern and South-Eastern Asia**											
Girault, 2004 [47]	Cambodia	2000	Low	Cross-sectional	TLS	RPR+TPPA	14.1%	11	206	5.3%	14
Sopheab, 2005 [48]	Cambodia	2005	Low	Cross-sectional	RDS	RPR+TPPA	5.2%	5	541	0.9%	13
Jiang, 2006 [49]	China	2003	Lower-middle	Cross-sectional	Convenience	RPR≥1:8+TPPA	0.0%	10	144	6.9%	12
Ma, 2007 [50]	China	2004	Lower-middle	Cross-sectional	RDS	RPR+ELISA	1.5%	25	325	7.7%	13
He, 2006 [51]	China	2004	Lower-middle	Cross-sectional	Convenience	TRUST+ELISA	0.0%	21	200	10.5%	9
Choi, 2007 [52]	China	2004-2005	Lower-middle	Cross-sectional	Snowball	TRUST+TPPA	1.5%	64	475	13.5%	11
Ruan, 2007 [53]	China	2005	Lower-middle	Cross-sectional	Convenience	TPPA	3.2%	59	526	11.2%	12
Ma, 2007 [50]	China	2005	Lower-middle	Cross-sectional	RDS	RPR+ELISA	6.1%	54	427	12.6%	13
Zhang, 2007 [54]	China	2005-2006	Lower-middle	Cross-sectional	VCT	RPR+TPHA	2.1%	53	753	7.0%	13
Ye, 2012 [55]	China	2006	Lower-middle	Cross-sectional	Snowball	RPR+TPPA	2.3%	518	5076	10.2%	11
Xiao, 2010 [56]	China	2006	Lower-middle	Cross-sectional	Snowball	RPR+TPPA	2.9%	490	4983	9.8%	11

Study	Country	Study year(s)	Income level	Study design	Sampling method*	Diagnostic method	HIV prevalence (crude)	No. syphilis positive	No. syphilis tested	Syphilis prevalence (crude)†	Axis score
Ma, 2007 [50]	China	2006	Lower-middle	Cross-sectional	RDS	RPR+ELISA	6.9%	54	540	10.0%	13
Zhang, 2012 [57]	China	2006	Lower-middle	Cross-sectional	Snowball	RPR+TPPA	10.9%	89	953	9.3%	13
Zeng, 2014 [58]	China	2006	Lower-middle	Cross-sectional	Snowball	ELISA	13.0%	54	561	9.6%	12
He, 2009 [59]	China	2006	Lower-middle	Cross-sectional	RDS	RPR+ELISA	1.7%	20	409	4.9%	11
Wang, 2012 [60]	China	2006	Lower-middle	Cross-sectional	Snowball	RPR+TPPA	1.0%	37	401	9.2%	13
Guo, 2009 [61]	China	2006	Lower-middle	Cross-sectional	Convenience	RPR+TPPA	4.7%	79	296	26.7%	10
Xu, 2010 [62]	China	2006	Lower-middle	Cohort	Convenience	RPR+TPPA	0.0%	73	218	33.5%	13
Feng, 2008 [63]	China	2005-2007	Lower-middle	Cross-sectional	Convenience	TRUST+TPPA	3.3%	264	1376	19.2%	12
Ruan, 2009 [64]	China	2006-2007	Lower-middle	Cross-sectional	Convenience	TPHA	4.8%	107	541	19.8%	12
Ye, 2012 [55]	China	2007	Lower-middle	Cross-sectional	Snowball	RPR+TPPA	3.4%	538	4981	10.8%	11
Zheng, 2012 [65]	China	2007	Lower-middle	Cross-sectional	Convenience	RPR+TPPA	2.5%	11	157	7.0%	12
Zou, 2010 [66]	China	2007	Lower-middle	Cross-sectional	Convenience	TPPA	4.8%	48	420	11.4%	11
Feng, 2010 [67]	China	2007	Lower-middle	Cross-sectional	Snowball	ELISA	9.1%	144	513	28.1%	11
Xiao, 2009 [68]	China	2007	Lower-middle	Cross-sectional	Convenience	RPR+TPPA	10.9%	56	742	7.5%	11
Zhang, 2012 [57]	China	2007	Lower-middle	Cross-sectional	Snowball	RPR+TPPA	12.8%	89	1015	8.8%	13
Wang, 2012 [60]	China	2007	Lower-middle	Cross-sectional	Snowball	RPR+TPPA	2.9%	65	419	15.5%	13
Guo, 2013 [69]	China	2007	Lower-middle	Cohort	Convenience	RPR+TPHA	8.1%	35	161	21.7%	13
Zhu, 2008 [70]	China	2007	Lower-middle	Cross-sectional	Convenience	ELISA	3.9%	18	73	24.7%	12
Guo, 2009 [61]	China	2007	Lower-middle	Cross-sectional	Convenience	RPR+TPPA	8.0%	41	137	29.9%	10
Ye, 2012 [55]	China	2008	Lower-middle	Cross-sectional	Snowball	RPR+TPPA	4.9%	748	5460	13.7%	11
Wang, 2014 [71]	China	2008	Lower-middle	Cross-sectional	Convenience	RPR+ELISA	1.7%	59	1146	5.1%	13
Li, 2011 [72]	China	2008	Lower-middle	Cross-sectional	Convenience	TPPA	4.5%	161	550	29.3%	12
She, 2013 [73]	China	2008	Lower-middle	Cross-sectional	Snowball	RPR+TPPA	6.9%	206	1693	12.2%	11
Zhang, 2012 [57]	China	2008	Lower-middle	Cross-sectional	Snowball	RPR+TPPA	16.6%	63	743	8.5%	13
Zeng, 2014 [58]	China	2008	Lower-middle	Cross-sectional	Snowball	ELISA	16.3%	70	602	11.6%	12
Ma, 2016 [74]	China	2008	Lower-middle	Cross-sectional	RDS	RPR+TPPA	16.2%	72	617	11.7%	13
Zhong, 2014 [75]	China	2008	Lower-middle	Cross-sectional	Snowball	RPR+TPPA	5.0%	66	379	17.4%	14
Wang, 2012 [60]	China	2008	Lower-middle	Cross-sectional	Snowball	RPR+TPPA	3.5%	65	451	14.4%	13
Yan, 2016 [76]	China	2008	Lower-middle	Cross-sectional	RDS	RPR+TPPA	4.7%	54	430	12.6%	13
Cai, 2010¶ [77]	China	2008	Lower-middle	Cross-sectional	TLS	RPR+TPPA	5.3%	56	394	14.2%	14
Bai, 2011 [78]	China	2008	Lower-middle	Cross-sectional	Snowball	RPR/TRUST+TPPA	7.1%	42	280	15.0%	11
Xu, 2011 [79]	China	2008-2009	Lower-middle	Cross-sectional	Convenience	RPR+TPPA	3.0%	22	436	5.0%	13
Wu, 2013 [80]	China	2008-2009	Lower-middle	Cross-sectional	Snowball	RPR/TRUST+TPPA	4.9%	5552	47231	11.8%	13
Chen, 2016 [81]	China	2008-2009	Lower-middle	Cross-sectional	RDS	RPR+TPPA	7.7%	422	2958	14.3%	11
Cao, 2014 [82]	China	2008-2009	Lower-middle	Cross-sectional	Snowball	RPR+TPPA	8.0%	154	858	17.9%	12
Gao, 2015 [83]	China	2008-2009	Lower-middle	Cross-sectional	Snowball	RPR+TPPA	6.0%	108	600	18.0%	12
Zhao, 2014 [84]	China	2008-2009	Lower-middle	Cross-sectional	TLS	RPR+TPPA	7.0%	162	801	20.2%	15
Liao, 2011 [85]	China	2008-2009	Lower-middle	Cross-sectional	Convenience	RPR+TPPA	2.5%	166	2994	5.5%	11
Qin, 2016 [86]	China	2009	Lower-middle	Cross-sectional	Snowball	RPR+ELISA	5.5%	1586	17431	9.1%	12
Ye, 2012 [55]	China	2009	Lower-middle	Cross-sectional	Snowball	RPR+TPPA	5.3%	692	5326	13.0%	11
Wang, 2014 [71]	China	2009	Lower-middle	Cross-sectional	Convenience	RPR+ELISA	1.3%	90	1351	6.7%	13
Fan, 2012 [87]	China	2009	Lower-middle	Cross-sectional	RDS	RPR+TPPA	7.2%	110	500	22.0%	12
Mao, 2014 [88]	China	2009	Lower-middle	Cohort	Convenience	RPR+TPPA	6.6%	162	547	29.6%	12
Li, 2012 [89]	China	2009	Lower-middle	Cohort	Convenience	RPR+TPPA	0.0%	131	797	16.4%	12
Chen, 2015 [90]	China	2009	Lower-middle	Cross-sectional	Snowball	RPR+TPPA	12.3%	61	826	7.4%	12
Zhang, 2012 [91]	China	2009	Lower-middle	Cross-sectional	RDS	RPR+TPPA	11.5%	26	503	5.2%	12
Zhang, 2012 [57]	China	2009	Lower-middle	Cross-sectional	Snowball	RPR+TPPA	19.1%	72	603	11.9%	13
Zhong, 2014 [75]	China	2009	Lower-middle	Cross-sectional	Snowball	RPR+TPPA	3.9%	32	385	8.3%	14
Wang, 2012 [60]	China	2009	Lower-middle	Cross-sectional	Snowball	RPR+TPPA	5.1%	101	450	22.4%	13

Study	Country	Study year(s)	Income level	Study design	Sampling method*	Diagnostic method	HIV prevalence (crude)	No. syphilis positive	No. syphilis tested	Syphilis prevalence (crude)†	Axis score
Yang, 2014 [92]	China	2009	Lower-middle	Cross-sectional	Convenience	TRUST+ELISA	14.7%	178	839	21.2%	13
Jia, 2015 [93]	China	2007-2012	Lower-middle	Cohort	Convenience	RPR+TPPA	8.4%	481	5800	8.3%	12
Li, 2016 [94]	China	2009-2010	Lower-middle	Cohort	Convenience	RPR+TPPA	6.3%	170	962	17.7%	12
Wang, 2014 [95]	China	2009-2010	Lower-middle	Cohort	Snowball	TRUST+ELISA/TPPA	10.5%	118	622	19.0%	15
Huan, 2011 [96]	China	2010	Upper-middle	Cross-sectional	Snowball	TRUST≥1:8+ELISA	-	44	328	13.4%	11
Qin, 2016 [86]	China	2010	Upper-middle	Cross-sectional	Snowball	RPR+ELISA	5.7%	2940	34191	8.6%	12
Luo, 2012 [97]	China	2010	Upper-middle	Cross-sectional	Snowball	RPR+TPPA	8.2%	44	1118	3.9%	11
Chen, 2017 [98]	China	2010	Upper-middle	Cross-sectional	Snowball	TRUST+ELISA	8.9%	410	3061	13.4%	13
Hu, 2017 [99]	China	2010	Upper-middle	Cross-sectional	Snowball	RPR+ELISA	1.0%	145	3087	4.7%	12
Wang, 2014 [71]	China	2010	Upper-middle	Cross-sectional	Convenience	RPR+ELISA	3.2%	99	1257	7.9%	13
Zhang, 2013 [100]	China	2010	Upper-middle	Cross-sectional	Snowball	RPR+ELISA	9.9%	58	302	19.2%	13
Zeng, 2014 [58]	China	2010	Upper-middle	Cross-sectional	Snowball	TRUST+ELISA	12.5%	25	400	6.3%	12
Zhong, 2014 [75]	China	2010	Upper-middle	Cross-sectional	Snowball	RPR+TPPA	7.7%	28	405	6.9%	14
Wu, 2016 [101]	China	2010	Upper-middle	Cross-sectional	Convenience	TPPA	3.4%	23	522	4.4%	13
Wang, 2012 [60]	China	2010	Upper-middle	Cross-sectional	Snowball	RPR+TPPA	7.5%	65	413	15.7%	13
Xu, 2013 [102]	China	2009-2011	Upper-middle	Cohort	Convenience	RPR+TPPA	0.0%	22	378	5.8%	13
Zhao, 2015 [103]	China	2010	Upper-middle	Cross-sectional	RDS	RPR+TPPA	9.5%	122	621	19.6%	15
Zhao, 2015 [103]	China	2010	Upper-middle	Cross-sectional	TLS	RPR+TPPA	9.4%	75	533	14.1%	17
Zhou, 2011 [104]	China	2010	Upper-middle	Cross-sectional	Snowball	RPR+TPPA	2.3%	43	400	10.8%	9
Liu, 2013 [105]	China	2010	Upper-middle	Cross-sectional	Convenience	ELISA	13.1%	40	388	10.3%	12
Tao, 2013 [106]	China	2010-2011	Upper-middle	Cross-sectional	Snowball	RPR+ELISA	5.4%	150	894	16.8%	11
Fan, 2014 [107]	China	2010-2011	Upper-middle	Cross-sectional	Snowball	RPR+TPPA	5.3%	148	887	16.7%	11
Zhang, 2015 [108]	China	2009-2012	Upper-middle	Cross-sectional	Snowball	RPR+TPPA	7.9%	187	1230	15.2%	13
Huang, 2014 [109]	China	2009-2012	Upper-middle	Cross-sectional	RDS	RPR+TPPA	15.4%	61	1239	4.9%	11
Cai, 2014 [110]	China	2009-2012	Upper-middle	Cross-sectional	Snowball	TRUST+TPPA	8.0%	179	813	22.0%	10
Wang, 2012 [111]	China	2011	Upper-middle	Cross-sectional	Snowball	RPR/TRUST+ELISA	6.3%	2902	37084	7.8%	10
Qin, 2016 [86]	China	2011	Upper-middle	Cross-sectional	Snowball	RPR+ELISA	6.3%	2894	37099	7.8%	12
Qi, 2015 [112]	China	2011	Upper-middle	Cross-sectional	Convenience	RPR+rapid test	6.3%	2189	32701	6.7%	12
Chen, 2017 [98]	China	2011	Upper-middle	Cross-sectional	Snowball	TRUST+ELISA	7.9%	300	3337	9.0%	13
Lan, 2014 [113]	China	2011	Upper-middle	Cross-sectional	Convenience	RPR/TRUST+TPPA	3.0%	53	462	11.5%	10
Hu, 2016 [114]	China	2011	Upper-middle	Cross-sectional	Convenience	TRUST+ELISA	8.1%	249	2433	10.2%	12
Hu, 2017 [99]	China	2011	Upper-middle	Cross-sectional	Snowball	RPR+ELISA	1.9%	156	3192	4.9%	12
Wang, 2014 [71]	China	2011	Upper-middle	Cross-sectional	Convenience	RPR+ELISA	2.7%	81	1314	6.2%	13
Wang, 2016 [115]	China	2011	Upper-middle	Cross-sectional	RDS	RPR+TPPA	19.2%	16	391	4.1%	11
Zhong, 2014 [75]	China	2011	Upper-middle	Cross-sectional	Snowball	RPR+TPPA	9.3%	25	400	6.3%	14
Luo, 2015¶ [116]	China	2011	Upper-middle	Cross-sectional	Snowball	RPR+TPHA	10.4%	37	259	14.3%	13
Zhang, 2013 [117]	China	2011	Upper-middle	Cross-sectional	Snowball	RPR+TPPA	9.5%	66	463	14.3%	13
Liao, 2014 [118]	China	2011	Upper-middle	Cross-sectional	Convenience	RPR+TPPA	1.6%	84	1228	6.8%	13
Wu, 2018 [119]	China	2011	Upper-middle	Cross-sectional	Snowball	RPR+ELISA	3.8%	28	400	7.0%	11
Fan, 2016 [120]	China	2011-2012	Upper-middle	Cross-sectional	Convenience	TPPA	19.2%	16	391	4.1%	11
Pan, 2016 [121]	China	2009-2014	Upper-middle	Cross-sectional	VCT	RPR+TPPA	11.9%	232	1981	11.7%	12
Qu, 2016 [122]	China	2012	Upper-middle	Cross-sectional	Snowball	TRUST+ELISA	6.8%	381	1611	23.6%	12
Qin, 2016 [86]	China	2012	Upper-middle	Cross-sectional	Snowball	RPR+ELISA	6.7%	2993	39910	7.5%	12
Chen, 2017 [98]	China	2012	Upper-middle	Cross-sectional	Snowball	TRUST+ELISA	9.6%	298	4380	6.8%	13
Hu, 2016 [114]	China	2012	Upper-middle	Cross-sectional	Convenience	TRUST+ELISA	9.6%	196	2678	7.3%	12
Hu, 2017 [99]	China	2012	Upper-middle	Cross-sectional	Snowball	RPR+ELISA	2.2%	170	3202	5.3%	12
Liang, 2014 [123]	China	2012	Upper-middle	Cross-sectional	Snowball	TRUST+EIA	11.1%	147	2783	5.3%	10
Wang, 2014 [71]	China	2012	Upper-middle	Cross-sectional	Convenience	RPR+ELISA	3.7%	109	1301	8.4%	13

Study	Country	Study year(s)	Income level	Study design	Sampling method*	Diagnostic method	HIV prevalence (crude)	No. syphilis positive	No. syphilis tested	Syphilis prevalence (crude)†	Axis score
Zeng, 2014 [58]	China	2012	Upper-middle	Cross-sectional	Snowball	TRUST+ELISA	19.5%	11	390	2.8%	12
Zhong, 2014 [75]	China	2012	Upper-middle	Cross-sectional	Snowball	RPR+TPPA	10.0%	36	401	9.0%	14
Yan, 2016 [76]	China	2012	Upper-middle	Cross-sectional	RDS	RPR+TPPA	9.2%	67	589	11.4%	13
Chu, 2018 [124]	China	2011-2013	Upper-middle	Cohort	Snowball	RPR+TPPA	9.1%	69	657	10.5%	12
Wu, 2018 [119]	China	2012	Upper-middle	Cross-sectional	Snowball	RPR+ELISA	7.3%	25	400	6.3%	11
Mao, 2018 [125]	China	2012-2013	Upper-middle	Cross-sectional	Convenience	RPR+TPPA	9.9%	381	4496	8.5%	14
Xu, 2014 [126]	China	2012-2013	Upper-middle	Cross-sectional	Convenience	RPR+TPPA	9.2%	466	3830	12.2%	12
Qin, 2016 [86]	China	2013	Upper-middle	Cross-sectional	Snowball	RPR+ELISA	7.3%	2689	42680	6.3%	12
Chen, 2017 [98]	China	2013	Upper-middle	Cross-sectional	Snowball	TRUST+ELISA	8.6%	231	4208	5.5%	13
Guanghua, 2018 [127]	China	2013	Upper-middle	Cross-sectional	Convenience	RPR+ELISA	6.6%	185	1996	9.3%	14
Hu, 2016 [114]	China	2013	Upper-middle	Cross-sectional	Convenience	TRUST+ELISA	8.8%	169	2591	6.5%	12
Hu, 2017 [99]	China	2013	Upper-middle	Cross-sectional	Snowball	RPR+ELISA	3.6%	193	3164	6.1%	12
Zeng, 2014 [58]	China	2013	Upper-middle	Cross-sectional	Snowball	TRUST+ELISA	19.7%	11	376	2.9%	12
Zhang, 2019 [128]	China	2013	Upper-middle	Cross-sectional	Snowball	RPR+TRUST+ELISA	23.0%	28	875	3.2%	13
Zhong, 2014 [75]	China	2013	Upper-middle	Cross-sectional	Snowball	RPR+TPPA	11.4%	21	633	3.3%	14
Wu, 2019 [129]	China	2013	Upper-middle	Case-control	Convenience	TRUST+TPPA	.	71	447	15.9%	13
Ding, 2014 [130]	China	2013	Upper-middle	Cross-sectional	Snowball	Syphilis antibody	1.3%	2	151	1.3%	10
Zhang, 2018 [131]	China	2012-2014	Upper-middle	Cross-sectional	Convenience	RPR+TPPA	10.8%	71	646	11.0%	13
Cai, 2016 [132]	China	2011-2015	Upper-middle	Cross-sectional	VCT	TRUST+TPPA	9.8%	556	3040	18.3%	11
Wu, 2018 [119]	China	2013	Upper-middle	Cross-sectional	Snowball	RPR+ELISA	5.5%	22	400	5.5%	11
Liu, 2016 [133]	China	2013-2014	Upper-middle	Cross-sectional	Convenience	TRUST+TPPA	12.7%	269	3588	7.5%	12
Li, 2016 [134]	China	2013-2014	Upper-middle	Cross-sectional	RDS	TRUST+TPPA	14.7%	201	1316	15.3%	13
Liu, 2016 [135]	China	2013-2014	Upper-middle	Cross-sectional	Snowball	ELISA	12.4%	56	587	9.5%	13
Chen, 2017 [98]	China	2014	Upper-middle	Cross-sectional	Snowball	TRUST+ELISA	8.9%	324	4766	6.8%	13
Guanghua, 2018 [127]	China	2014	Upper-middle	Cross-sectional	Convenience	RPR+ELISA	8.4%	193	1965	9.8%	14
Hu, 2016 [114]	China	2014	Upper-middle	Cross-sectional	Convenience	TRUST+ELISA	10.6%	204	2610	7.8%	12
Hu, 2017 [99]	China	2014	Upper-middle	Cross-sectional	Snowball	RPR+ELISA	4.4%	138	3060	4.5%	12
Zhang, 2019 [128]	China	2014	Upper-middle	Cross-sectional	Snowball	RPR+TRUST+ELISA	21.5%	66	1149	5.7%	13
Huang, 2016 [136]	China	2014	Upper-middle	Cross-sectional	TLS	RPR+TPPA	9.7%	105	965	10.9%	14
Wu, 2018 [119]	China	2014	Upper-middle	Cross-sectional	Snowball	RPR+ELISA	6.0%	26	400	6.5%	11
Li, 2019 [137]	China	2014-2015	Upper-middle	Cohort	Convenience	RPR+TPPA	0.0%	34	523	6.5%	14
Zhang, 2017 [138]	China	2014-2015	Upper-middle	Cross-sectional	Convenience	TRUST+TPPA	17.0%	34	300	11.3%	14
Lu, 2017 [139]	China	2015	Upper-middle	Cross-sectional	Snowball	TRUST+ELISA	9.8%	10	102	9.8%	11
Guanghua, 2018 [127]	China	2015	Upper-middle	Cross-sectional	Convenience	RPR+ELISA	11.2%	104	1697	6.1%	14
Hu, 2016 [114]	China	2015	Upper-middle	Cross-sectional	Convenience	TRUST+ELISA	10.1%	212	2541	8.3%	12
Zhang, 2019 [128]	China	2015	Upper-middle	Cross-sectional	Snowball	RPR+TRUST+ELISA	20.3%	71	1329	5.3%	13
Liu, 2017 [140]	China	2015	Upper-middle	Cross-sectional	VCT	RPR+TPPA	11.6%	53	732	7.2%	13
An, 2019 [141]	China	2015	Upper-middle	Cross-sectional	Convenience	RPR+TPPA	22.5%	33	267	12.4%	15
Wu, 2018 [119]	China	2015	Upper-middle	Cross-sectional	Snowball	RPR+ELISA	8.3%	20	400	5.0%	11
Zhang, 2019 [128]	China	2016	Upper-middle	Cross-sectional	Snowball	RPR+TRUST+ELISA	19.8%	107	1604	6.7%	13
Wei, 2019 [142]	China	2015-2017	Upper-middle	Cross-sectional	TLS	RPR+TPPA	13.5%	484	3613	13.4%	11
Bai, 2019 [143]	China	2016	Upper-middle	Cross-sectional	Convenience	TRUST+ELISA	3.8%	32	400	8.0%	11
Wu, 2018 [119]	China	2016	Upper-middle	Cross-sectional	Snowball	RPR+ELISA	9.0%	34	400	8.5%	11
Zhang, 2019 [128]	China	2017	Upper-middle	Cross-sectional	Snowball	RPR+TRUST+ELISA	18.9%	102	1527	6.7%	13
Bai, 2019 [143]	China	2017	Upper-middle	Cross-sectional	Convenience	TRUST+ELISA	3.3%	17	400	4.3%	11
Wu, 2018 [119]	China	2017	Upper-middle	Cross-sectional	Snowball	RPR+ELISA	6.3%	43	400	10.8%	11
Liao, 2019 [144]	China	2018	Upper-middle	Cross-sectional	Snowball	TRUST+ELISA	3.0%	96	3474	2.8%	12
Bai, 2019 [143]	China	2018	Upper-middle	Cross-sectional	Convenience	TRUST+ELISA	3.0%	31	400	7.8%	11
Pisani, 2004 [145]	Indonesia	2002	Low	Cross-sectional	Convenience	RPR+TPPA	9.0%	131	767	17.1%	14

Study	Country	Study year(s)	Income level	Study design	Sampling method*	Diagnostic method	HIV prevalence (crude)	No. syphilis positive	No. syphilis tested	Syphilis prevalence (crude)†	Axis score
MOH, 2013 [146]	Indonesia	2013	Lower-middle	Cross-sectional	RDS	RPR+TPHA	17.3%	122	960	12.7%	11
MOH, 2013¶ [146]	Indonesia	2013	Lower-middle	Cross-sectional	Probability	RPR+TPHA	19.0%	159	980	16.2%	11
Itoda, 2013 [147]	Japan	2008-2011	High	Cross-sectional	VCT	POCT	3.1%	43	423	10.2%	13
Wickersham, 2017¶ [148]	Malaysia	2014	Upper-middle	Cross-sectional	RDS	RPR+TPPA	12.4%	53	187	28.3%	15
Davaalkham, 2009 [149]	Mongolia	2007	Lower-middle	Cross-sectional	Convenience	POCT	0.0%	15	50	30.0%	11
MOH, 2012 [150]	Mongolia	2012	Lower-middle	Cross-sectional	RDS	RPR≥1:2+TPPA	10.7%	8	196	4.1%	14
MOH, 2014 [151]	Mongolia	2014	Upper-middle	Cross-sectional	RDS	RPR+TPHA	13.7%	18	255	7.1%	14
MOH, 2007 [152]	Myanmar	2007	Low	Cross-sectional	Convenience	VDRL	29.3%	28	400	7.0%	11
MOH, 2009 [153]	Myanmar	2008	Low	Cross-sectional	Convenience	VDRL	28.8%	56	397	14.1%	12
MOH, 2010 [154]	Myanmar	2009	Low	Cross-sectional	Convenience	VDRL	22.3%	25	400	6.3%	12
MOH, 2011 [155]	Myanmar	2010	Low	Cross-sectional	Convenience	VDRL	11.0%	4	400	1.0%	12
MOH, 2012 [156]	Myanmar	2011	Low	Cross-sectional	Convenience	VDRL	7.8%	10	400	2.5%	11
MOH, 2013 [157]	Myanmar	2012	Low	Cross-sectional	Convenience	VDRL	8.9%	25	763	3.3%	11
Dept of Health, 2014¶ [158]	Philippines	2013	Lower-middle	Cross-sectional	Probability	TPPA	3.7%	7	296	2.4%	12
Jung, 2012 [159]	South Korea	2008	High	Cross-sectional	TLS	TPPA	6.5%	22	108	20.4%	13
Ananwaranich, 2013 [160]	Thailand	2005	Lower-middle	Cross-sectional	VCT	RPR+POCT	24.6%	10	200	5.0%	10
Ananwaranich, 2013 [160]	Thailand	2006	Lower-middle	Cross-sectional	VCT	RPR+POCT	23.0%	16	505	3.2%	10
Ananwaranich, 2013 [160]	Thailand	2007	Lower-middle	Cross-sectional	VCT	RPR+POCT	25.3%	27	440	6.1%	10
Holtz, 2012 [161]	Thailand	2006-2010	Lower-middle	Cross-sectional	Convenience	RPR≥1:8+POCT	21.6%	68	1541	4.4%	14
Ananwaranich, 2013 [160]	Thailand	2008	Lower-middle	Cross-sectional	VCT	RPR+POCT	26.6%	27	611	4.4%	10
Ananwaranich, 2013 [160]	Thailand	2009	Lower-middle	Cross-sectional	VCT	RPR+POCT	27.4%	63	707	8.9%	10
Van Griensven, 2013 [162]	Thailand	2006-2012	Lower-middle	Cohort	Convenience	RPR≥1:8+POCT	21.3%	82	1744	4.7%	12
Ananwaranich, 2013 [160]	Thailand	2010	Upper-middle	Cross-sectional	VCT	RPR+POCT	34.1%	157	867	18.1%	10
Ananwaranich, 2013 [160]	Thailand	2011	Upper-middle	Cross-sectional	VCT	RPR+POCT	29.4%	124	994	12.5%	10
Phanuphak, 2018 [163]	Thailand	2016-2017	Upper-middle	Cross-sectional	Convenience	RPR+TPHA	0.0%	113	1626	6.9%	13
Pisani, 2006 [164]	Timor -Leste	2003	Low	Cross-sectional	Convenience	RPR+TPHA	0.9%	17	110	15.5%	10
ASHM, 2017 [165]	Timor-Leste	2017	Lower-middle	Cross-sectional	RDS	POCT	0.3%	21	310	6.8%	15
Pham, 2012 [166]	Vietnam	2009	Lower-middle	Cross-sectional	Convenience	TPHA	6.3%	5	381	1.3%	12
MOH, 2011 [167]	Vietnam	2009-2010	Lower-middle	Cross-sectional	RDS	RPR+TPHA	13.7%	17	1595	1.1%	15
Nguyan, 2016 [168]	Vietnam	2010-2012	Lower-middle	Cross-sectional	Convenience	RPR+TPHA	2.6%	40	2467	1.6%	14
Colby, 2016¶ [169]	Vietnam	2015	Lower-middle	Cross-sectional	Snowball	RPR+POCT	18.0%	36	205	17.6%	12
**Europe and Northern America**											
Revniers, 2018 [170]	Belgium	2015-2016	High	Cross-sectional	Convenience	RPR≥1:4 +TPPA/TPA	0.0%	15	200	7.5%	15
Ravlija, 2011 [171]	Bosnia- Herzegovina	2010-2011	Upper-middle	Cross-sectional	Snowball	ELISA	1.8%	1	168	0.6%	12
Ravlija, 2012 [172]	Bosnia- Herzegovina	2012	Upper-middle	Cross-sectional	Snowball	ELISA	1.2%	2	330	0.6%	12
Nelson, 2019 [173]	Canada	2011-2013	High	Cross-sectional	Convenience	EIA	38.4%	15	86	17.4%	12
Bozicevic, 2009 [174]	Croatia	2006	Upper-middle	Cross-sectional	RDS	TPHA	5.0%	31	360	8.6%	15
Bozicevic, 2012 [175]	Croatia	2010-2011	High	Cross-sectional	RDS	RPR+TPHA	3.6%	3	387	0.8%	15
Vilibic-Cavlek, 2018 [176]	Croatia	2011-2013	High	Cross-sectional	VCT	ELISA+Western Blotting	3.3%	11	90	12.2%	12
Ruutel, 2015 [177]	Estonia	2013	High	Cross-sectional	Convenience	Syphilis antibody	7.0%	2	43	4.7%	15
Zorzi, 2017 [178]	Itary	2013-2014	High	Cross-sectional	RDS	RPR+TPPA	.	16	289	5.5%	14
Mikik, 2011 [179]	Macedonia	2010	Upper-middle	Cross-sectional	RDS	Real time PCR (blood)	0.5%	2	382	0.5%	12
MOH, 2014 [180]	Macedonia	2013-2014	Upper-middle	Cross-sectional	RDS	Syphilis antibody+TPH-test	2.3%	5	426	1.2%	13
WHO (Europe), 2004 [181]	Moldova	2003	Low	Cross-sectional	Convenience	Treponema antibody	1.7%	12	99	12.1%	11
NCHM, 2008 [182]	Moldova	2007	Lower-middle	Cross-sectional	Convenience	TPHA	4.8%	2	31	6.5%	14
Zohrabyan, 2013 [183]	Moldova	2010	Lower-middle	Cross-sectional	RDS	TPHA	1.5%	27	397	6.8%	13
Hoornenborg, 2018 [184]	Netherland	2015-2016	High	Cross-sectional	Convenience	CLIA	0.0%	6	374	1.6%	13
Amirkhanian, 2006 [185]	Russia	2003	Lower-middle	Cross-sectional	Convenience	Wasserman reaction assay+TPHA	.	13	125	10.4%	10
WHO (Europe), 2004 [181]	Russia	2003	Lower-middle	Cross-sectional	Snowball	Treponema antibody	2.5%	21	253	8.3%	11

Study	Country	Study year(s)	Income level	Study design	Sampling method*	Diagnostic method	HIV prevalence (crude)	No. syphilis positive	No. syphilis tested	Syphilis prevalence (crude)†	Axis score
Baral, 2010¶ [186]	Russia	2005-2006	Upper-middle	Cohort	RDS	RPR	16.0%	6	50	12.0%	11
WHO (Europe), 2007 [187]	Russia	2006	Upper-middle	Cross-sectional	Convenience	RPGA	2.2%	10	558	1.8%	15
Wirtz, 2015 [188]	Russia	2012-2013	High	Cross-sectional	RDS	RPR+RPGA	47.2%	20	123	16.3%	14
MOH, 2012 [189]	Serbia	2012	Upper-middle	Cross-sectional	Snowball	POCT	3.8%	46	400	11.5%	13
Tahirukaj, 2014 [190]	Serbia (Kosovo)	2014	Lower-middle	Cross-sectional	RDS	Syphilis antibody	2.3%	9	217	4.1%	15
Hurtado, 2011 [191]	Spain	2000-2006	High	Cross-sectional	VCT	RPR+ELISA±FTA-ABS	.	33	1387	2.4%	9
Hurtado, 2010 [192]	Spain	2008	High	Cross-sectional	Convenience	POCT	4.1%	6	141	4.3%	11
Borelli, 2009 [193]	Switzerland	2004	High	Cross-sectional	Convenience	TPPA	.	40	610	6.6%	10
Manavi, 2012 [194]	United Kingdom	2009-2010	High	Cross-sectional	Convenience	POCT	1.5%	1	390	0.3%	8
Golden, 2006 [195]	United States	2002-2005	High	Cross-sectional	Convenience	RPR+TPPA	5.0%	1	445	0.2%	12
Mayer, 2012 [196]	United States	2004-2010	High	Cross-sectional	Convenience	RPR+FTA-ABS	2.3%	17	850	2.0%	12
Katz, 2018 [197]	United States	2010-2014	High	RCT	Convenience	RPR+TPPA	0.0%	4	230	1.7%	15
Watson, 2019 [198]	United States	2013-2014	High	Cross-sectional	RDS	Treponema antibody	36.8%	143	442	32.4%	13
Fujimoto, 2018 [199]	United States	2014-2016	High	Cross-sectional	RDS	RPR≥1:4+FTA-ABS	40.5%	105	365	28.8%	12
Reback, 2018 [200]	United States	2014-2016	High	Cohort	Convenience	RPR+FTA-ABS	41.6%	38	286	13.3%	12
Obafemi, 2019 [201]	United States	2016-2018	High	Cross-sectional	Convenience	RPR+TPPA	1.8%	38	2296	1.7%	11
Shannon, 2019 [202]	United States	2017-2019	High	Cross-sectional	Convenience	POCT	17.0%	62	788	7.9%	15
**Latin America and the Caribbean**											
Pando, 2006 [203]	Argentina	2000-2001	Upper-middle	Cross-sectional	Convenience	VDRL+TPHA±FTA-ABS	13.8%	112	662	16.9%	14
Berini, 2007 [204]	Argentina	2000-2003	Upper-middle	Cross-sectional	Convenience	VDRL+TPHA+FTA-ABS	13.2%	109	682	16.0%	12
Segura, 2010 [205]	Argentina	2003-2004	Upper-middle	Cohort	Convenience	RPR+TPPA/TPHA±FTA-ABS	7.5%	60	877	6.8%	9
Avila, 2017¶ [206]	Argentina	2006-2009	Upper-middle	Cross-sectional	Convenience	VDRL+TPHA±FTA-ABS	34.1%	130	258	50.4%	14
Pando, 2012 [207]	Argentina	2007-2009	Upper-middle	Cross-sectional	RDS	VDRL+TPHA±FTA-ABS	17.2%	102	496	20.6%	13
MOH, 2012 [208]	Belize	2012	Upper-middle	Cross-sectional	RDS	RPR+TPPA	13.8%	1	129	0.8%	11
MOH, 2010 [209]	Bolivia	2008	Lower-middle	Cross-sectional	RDS	RPR+TPPA	13.6%	72	811	8.9%	15
Soares, 2014 [210]	Brazil	2005-2006	Lower-middle	Cross-sectional	RDS	POCT	7.3%	62	558	11.1%	11
Ribeiro, 2012 [211]	Brazil	2007	Upper-middle	Cross-sectional	Convenience	ELISA	.	21	833	2.5%	13
Brignol, 2015 [212]	Brazil	2008-2009	Upper-middle	Cross-sectional	RDS	POCT	6.3%	32	363	8.8%	13
de Albuquerque, 2014 [213]	Brazil	2011	Upper-middle	Cross-sectional	Convenience	VDRL+MHA-TP	4.8%	10	62	16.1%	10
Fernandes, 2015 [214]	Brazil	2011-2013	Upper-middle	Cross-sectional	Convenience	VDRL+EIA	14.4%	149	430	34.7%	12
Hoagland, 2017 [215]	Brazil	2014-2015	Upper-middle	Cross-sectional	Convenience	RPR≥1:8+MHA-TP	0.0%	43	444	9.7%	15
Grinsztejn, 2017¶ [216]	Brazil	2015-2016	Upper-middle	Cross-sectional	RDS	VDRL≥1:8+TPHA	41.2%	112	342	32.7%	14
Da Motta, 2018 [217]	Brazil	2016	Upper-middle	Cross-sectional	Probability	VDRL+FTA-ABS	.	59	1279	4.6%	16
Ferreira, 2019¶ [218]	Brazil	2015-2017	Upper-middle	Cohort	RDS	VDRL≥1:8+POCT	54.0%	88	322	27.3%	11
MOH, 2010 [219]	Costa Rica	2009	Upper-middle	Cross-sectional	RDS	VDRL+TPHA	12.7%	54	300	18.0%	14
Brito, 2015 [220]	Dominican Republic	2013	Upper-middle	Cross-sectional	Snowball	VDRL+FTA-ABS	5.0%	13	100	13.0%	12
Hernandez, 2017 [221]	Ecuador	2010	Upper-middle	Cross-sectional	Convenience	VDRL+TPPA	9.5%	33	306	10.8%	15
Jacobson, 2014 [222]	Ecuador	2010-2011	Upper-middle	Cross-sectional	RDS	RPR≥1:8+FTA-ABS	12.8%	24	414	5.8%	14
Sanchez-Gomez, 2015 [223]	Ecuador	2011-2012	Upper-middle	Cross-sectional	RDS	RPR≥1:8+FTA-ABS	13.6%	25	370	6.8%	14
Ham, 2015 [224]	El Salvador	2007-2008	Lower-middle	Cross-sectional	RDS	RPR+TPPA	13.5%	70	704	9.9%	8
Creswell, 2012 [225]	El Salvador	2008	Lower-middle	Cross-sectional	RDS	RPR+TPHA	13.4%	94	690	13.6%	14
Lahuerta, 2011 [226]	Guatemala	2006-2009	Lower-middle	Cross-sectional	VCT	VDRL+TPHA	0.8%	0	385	0.0%	12
Ham, 2015 [224]	Guatemala	2012	Lower-middle	Cross-sectional	RDS	RPR≥1:8+TPPA	10.4%	11	541	2.0%	8
MOH, 2014 [227]	Guyana	2014	Lower-middle	Cross-sectional	Convenience	RPR	.	4	387	1.0%	15
OHMaSS, 2015 [228]	Haiti	2014-2015	Low	Cross-sectional	RDS	RPR+POCT	16.0%	175	1239	14.1%	16
Zalla, 2019 [229]	Haiti	2016-2017	Low	Cross-sectional	Probability	POCT	4.7%	49	488	10.0%	17
Ham, 2015 [224]	Honduras	2012	Lower-middle	Cross-sectional	RDS	RPR+TPPA	14.4%	54	677	8.0%	8
Figueroa, 2013 [230]	Jamaica	2007-2008	Upper-middle	Cross-sectional	Convenience	TRUST+TPPA	32.3%	12	201	6.0%	11
Logie, 2018¶ [231]	Jamaica	2015	Upper-middle	Cross-sectional	Snowball	POCT	.	8	83	9.6%	13

Study	Country	Study year(s)	Income level	Study design	Sampling method*	Diagnostic method	HIV prevalence (crude)	No. syphilis positive	No. syphilis tested	Syphilis prevalence (crude)†	Axis score
Pitpitan, 2015 [232]	Mexico	2012-2013	Upper-middle	Cross-sectional	RDS	RPR+TPHA	17.3%	14	191	7.3%	15
Martinez, 2019 [233]	Mexico	2016-2018	Upper-middle	Cross-sectional	VCT	POCT	16.7%	198	1398	14.2%	5
Ham, 2015 [224]	Nicaragua	2009-2010	Lower-middle	Cross-sectional	RDS	RPR+TPPA	7.8%	54	652	8.3%	8
Hakre, 2014 [234]	Panama	2011-2012	Upper-middle	Cross-sectional	RDS	RPR+TPHA	20.6%	141	603	23.4%	14
Sanchez, 2007 [235]	Peru	2000	Lower-middle	Cross-sectional	Snowball	RPR+MHA-TP	19.7%	201	1357	14.8%	14
Clark, 2007 [236]	Peru	2000-2001	Lower-middle	Cross-sectional	Probability	RPR+TPPA	0.0%	2	85	2.4%	15
Caceres, 2008 [237]	Peru	2001-2003	Lower-middle	Cross-sectional	Convenience	RPR+TPPA	9.6%	48	166	28.9%	12
Lama, 2006 [238]	Peru	2002-2003	Lower-middle	Cross-sectional	Snowball	RPR+MHA-TP	13.9%	440	3280	13.4%	13
Clark, 2008 [239]	Peru	2003-2004	Lower-middle	Cross-sectional	Convenience	RPR+TPPA	1.5%	5	197	2.5%	14
Park, 2016 [240]	Peru	2002-2005	Lower-middle	Cohort	Convenience	TPPA	.	124	913	13.6%	12
Snowden, 2010 [241]	Peru	2003-2005	Lower-middle	Cross-sectional	Convenience	RPR≥1:8+TPPA	11.9%	54	513	10.5%	12
Blas, 2007 [242]	Peru	2006	Lower-middle	Cross-sectional	Convenience	RPR	5.5%	6	78	7.7%	11
Perez-Brumer, 2013 [243]	Peru	2007	Lower-middle	Cross-sectional	Convenience	RPR≥1:16+TPPA	8.2%	8	122	6.6%	12
Silva-Santisteban, 2012¶ [244]	Peru	2009	Upper-middle	Cross-sectional	RDS	RPR≥1:8	29.8%	23	426	5.4%	14
Pizzicato, 2017 [245]	Peru	2011	Upper-middle	Cross-sectional	Convenience	RPR+MHA-TP	8.0%	706	5101	13.8%	13
Galea, 2015 [246]	Peru	2012-2013	Upper-middle	Cross-sectional	Convenience	POCT	0.0%	105	600	17.5%	12
Blair, 2019 [247]	Peru	2012-2014	Upper-middle	Cross-sectional	Convenience	RPR≥1:16+MHA-TP	.	143	898	15.9%	13
Allan-Blitz, 2019 [248]	Peru	2016	Upper-middle	Cross-sectional	Convenience	RPR+TPPA	22.8%	84	303	27.7%	11
**Northern Africa and Western Asia**											
MOH, 2011 [249]	Armenia	2010-2011	Lower-middle	Cross-sectional	RDS	RPR+TPHA	2.2%	4	270	1.5%	10
MOH, 2013 [250]	Armenia	2012	Lower-middle	Cross-sectional	RDS	RPR+TPHA	2.0%	7	300	2.3%	11
MOH, 2015 [251]	Armenia	2014	Lower-middle	Cross-sectional	RDS	RPR+TPHA	1.3%	6	300	2.0%	11
MOH, 2016 [252]	Armenia	2016	Lower-middle	Cross-sectional	RDS	RPR+TPHA	1.3%	1	400	0.3%	14
MOH, 2018 [253]	Armenia	2018	Upper-middle	Cross-sectional	RDS	Rapid test+TPHA	2.0%	4	700	0.6%	16
MOH, 2008 [254]	Azerbaijan	2007-2008	Lower-middle	Cross-sectional	Convenience	Syphilis antibody	1.0%	8	100	8.0%	11
MOH, 2015 [255]	Azerbaijan	2014-2015	Upper-middle	Cross-sectional	RDS	ELISA	2.2%	68	500	13.6%	13
Tanadgoma, 2005 [256]	Georgia	2005	Lower-middle	Cross-sectional	RDS	RPR+TPHA+ ELISA	4.3%	19	70	27.1%	12
Save the Children, 2008 [257]	Georgia	2007	Lower-middle	Cross-sectional	RDS	RPR+TPHA+ syphilis antibody	3.7%	44	140	31.4%	14
Curatio Intl, 2010 [258]	Georgia	2010	Lower-middle	Cross-sectional	RDS	TPHA	7.0%	93	271	34.3%	15
Curatio Intl, 2012 [259]	Georgia	2012	Lower-middle	Cross-sectional	RDS	TPHA	13.0%	71	216	32.9%	16
Curatio Intl, 2015 [260]	Georgia	2015	Upper-middle	Cross-sectional	RDS	ELISA	20.7%	138	415	33.3%	14
Curatio Intl, 2018 [261]	Georgia	2018	Upper-middle	Cross-sectional	RDS	RPR+TPHA+LIA	16.2%	60	621	9.7%	13
Johnston, 2013 [262]	Morocco	2010-2011	Lower-middle	Cross-sectional	RDS	VDRL+TPHA	4.9%	73	662	11.0%	11
**Oceania (excluding Australia and New Zealand)**											
Hakim, 2019 [263]	Papua New Guinea	2016-2017	Lower-middle	Cross-sectional	RDS	POCT	6.4%	49	860	5.7%	15
MOH, 2018 [264]	Solomon Islands	2017	Lower-middle	Cross-sectional	RDS	RPR≥1:8	0.0%	0	39	0.0%	17
MOH, 2008 [265]	Tonga	2007-2008	Lower-middle	Cross-sectional	Convenience	RPR≥1:8+TPPA	0.0%	1	100	1.0%	13
Veronese, 2015 [266]	Vanuatu	2011-2012	Lower-middle	Cross-sectional	RDS	RPR+POCT	0.0%	1	39	2.6%	15
**Sub-Saharan Africa**											
Kendall, 2014 [267]	Angola	2011	Upper-middle	Cross-sectional	RDS	POCT	8.2%	1	310	0.3%	14
MOH, 2013 [268]	Botswana	2012	Upper-middle	Cross-sectional	RDS	RPR+TPHA	13.1%	12	450	2.7%	15
Goodman, 2016 [269]	Brukina Faso	2013	Low	Cross-sectional	RDS	RPR+POCT	4.8%	44	665	6.6%	13
Park, 2013 [270]	Cameroon	2011	Lower-middle	Cross-sectional	RDS	RPR+TPHA	37.0%	2	511	0.4%	15
MoH, 2016 [271]	Cote d'Ivoire	2015-2016	Lower-middle	Cross-sectional	RDS	POCT	11.6%	6	1288	0.5%	17
Mason, 2013 [272]	Gambia	2011	Low	Cross-sectional	Snowball	RPR+TPHA	9.8%	1	205	0.5%	13
Sanders, 2013 [273]	Kenya	2005-2011	Low	Cohort	Convenience	RPR+TPHA	0.0%	7	449	1.6%	12
Muraguri, 2015 [274]	Kenya	2010	Low	Cross-sectional	RDS	RPR+TPHA	25.6%	5	563	0.9%	11
MoH, 2019 [275]	Lesotho	2018	Lower-middle	Cross-sectional	RDS	POCT+EIA	22.7%	92	825	11.2%	14
MOH, 2013¶ [276]	Liberia	2013	Low	Cross-sectional	Convenience	RPR+TPHA	19.8%	2	207	1.0%	12

Study	Country	Study year(s)	Income level	Study design	Sampling method*	Diagnostic method	HIV prevalence (crude)	No. syphilis positive	No. syphilis tested	Syphilis prevalence (crude)†	Axis score
Wirtz, 2013 [277]	Malawi	2011-2012	Low	Cross-sectional	RDS	TPHA	15.4%	18	338	5.3%	14
Wirtz, 2017 [278]	Malawi	2011-2014	Low	Cross-sectional	RDS	TPHA	17.3%	110	2442	4.5%	14
Wirtz, 2014 [279]	Malawi	2011-2014	Low	Cross-sectional	RDS	TPHA	17.3%	110	2441	4.5%	12
MOH, 2010 [280]	Mauritius	2010	Upper-middle	Cross-sectional	RDS	VDRL+TPHA	10.6%	21	362	5.8%	15
PILS, 2017¶ [281]	Mauritius	2017	Upper-middle	Cross-sectional	RDS	RPR/VDRL+TPHA	28.4%	53	109	48.6%	15
Merrigan, 2011 [282]	Nigeria	2007	Low	Cross-sectional	RDS	RPR+TPHA	12.4%	0	879	0.0%	14
Wade, 2005 [283]	Senegal	2004	Low	Cross-sectional	Snowball	RPR+TPHA	21.5%	21	442	4.8%	13
Wade, 2010 [284]	Senegal	2007	Low	Cross-sectional	Snowball	RPR+TPHA	21.8%	17	500	3.4%	12
Johnston, 2010 [285]	Tanzania	2007	Low	Cross-sectional	RDS	POCT	12.8%	2	509	0.4%	11
Ross, 2014 [286]	Tanzania	2012-2013	Low	Cross-sectional	RDS	POCT	23.7%	5	262	1.9%	12
Mmbaga, 2018 [287]	Tanzania	2014	Low	Cross-sectional	RDS	VDRL+TPHA	22.3%	7	646	1.1%	14
Horacio, 2019 [288]	Togo	2012-2013	Low	Cross-sectional	RDS	VDRL+TPHA	9.1%	7	683	1.0%	14
Ekouevi, 2019 [289]	Togo	2017	Low	Cross-sectional	RDS	POCT	22.0%	0	678	0.0%	13
Kim, 2016 [290]	Uganda	2008-2009	Low	Cross-sectional	RDS	RPR+ELISA	13.4%	30	290	10.3%	11

*If snowball sampling, RDS, or TLS was used with other convenience sampling methods, it was described and counted as snowball sampling, RDS, or TLS, respectively. †If a study did not have crude prevalence data of syphilis and there was no response from authors (or there was no contact information), the study was excluded from our study. ¶Data points exclusively with male sex workers, transgender women, transgender women sex workers. ‡All of the participants were male or transgender women sex workers. For male sex workers in these two studies, it is possible that heterosexual male sex workers were included to some extent.

Text S1. *Search terms*

Search on MEDLINE (full text and subject heading search terms)
(syphili* OR exp syphilis [MeSH] OR treponema pallidum OR treponema pallidum [MeSH] OR great pox) AND (men who have sex with men OR exp “Sexual and Gender Minorities” [MeSH] OR gay OR homosexual* OR Homosexuality, Male [MeSH] OR bisexual* OR Bisexuality [MeSH] OR transgender OR high risk population) AND limit to yr=“2000-Current” AND “each country name”
**Search on EMBASE (full text and subject heading search terms)**
(syphili* OR exp syphilis/ OR treponema pallidum OR Treponema pallidum/ OR great pox) AND (men who have sex with men OR men who have sex with men/ OR gay OR homosexual male/ OR homosexual* OR bisexual* OR bisexual male/ OR men who have sex with men and women/ OR transgender OR transgender/ OR high risk population OR high risk population/) AND limit to yr=“2000-Current” AND “each country name”
**Search on LILACS (full text and subject heading search terms)**
tw:((tw:(syphili*)) OR (mh:("syphilis")) OR (tw:(great pox)) OR (tw:(treponema pallidum)) OR (mh:("treponema pallidum")) AND (tw:(men who have sex with men)) OR (tw:(msm)) OR (tw:(gay*)) OR (tw:(bisexual*)) OR (tw:(homosexual*)) OR (tw:(transgender*)) OR (tw:(high risk population*)) OR (tw:(same sex*)) OR (mh:("homosexuality, male")) OR (mh:("bisexual")) OR (mh:("gay")) OR (mh:("transgender"))) AND (db:("LILACS")) AND (year_cluster:[2000 TO 2020])
**Search on African Index Medicus (AIM) (full text search terms)**
Syphili$ OR Treponema pallidum

Text S2. *Data extraction form*

**General information**						
Study ID (No.)			Date form completed	DD / MM / YYYY		
Name of person extracting data		1.	2.	3.		
Study title						
First author			Year of publication			
Source	Database / reference	1. MEDLINE or EMBASE	2. References of review studies			
	Other data sets	1. IBBS (for syphilis)	2. LILACS	3.AIM		
Included STIs in this article		1. Syphilis	2. Gonorrhoea	3. Chlamydia	4. Trichomoniasis	
**Study eligibility**						
Country		______________				
SDG region		1. Sub-Saharan Africa	2. Northern Africa & Western Asia	3. Central & Southern Asia	4. Eastern & South -Eastern Asia	
		5. Latin America & the Caribbean	6. Australia & New Zealand	7. Europe & Northern America	8. Oceania (excluding Australia & New Zealand)	
Study type		1. Cross sectional	2. Cohort	3. Case-control	4. Other studies using primary data (______________)	
Study duration		From DD / MM / YYYY		To DD / MM / YYYY		
Age of participants (years)		________ If available, mean/median (_____), range/IQR (_______)				
Location		1. Community			2. Clinic	
Population definition						
Sex at birth		1. Male	2. Female	3. Intersex		
Sexuality		1. MSM	2. Bisexual	3. Transgender	4. Heterosexual	5. Others (________)
Language (of full-text)		1. English		2. Others (______________)		
Eligibility criteria met?		1. Yes		2. No		
Reason for exclusion						
**For syphilis**						
Definition of syphilis diagnosis according to authors						
Diagnostic test		1. Both NTT & TT	2. NTT only	3. TT only		
Non-treponemal test (NTT)		1. RPR	2. VDRL	3. TRUST		
Treponemal test (TT)		1. TPPA	2.TPHA	3. FTA-ABS	4. MHA-TP	5. ELISA
		6. EIA	7.CIA	8. POCT (ICS)	9. Others ( )	
Any NTT titer information?		1. Yes		2. No		
NTT Cutoff titer for active syphilis		1. 1:8	2. 1:16	3. Other		
**Outcomes (Prevalence)**						
Reported prevalence		_______%		95% CI:		
Number tested				Number positive		
**Others**						
Reference to other relevant studies		1. Yes		2. No		
Notes		HIV prevalence = (No. tested positive) / (No. tested) = ____% (diagnosed with ______________________)				

Text S3. *Correction of observed prevalence*

We corrected errors of known magnitude with the following formula based on the sensitivity and specificity of each assay used.[291] If there was a range for either the sensitivity or specificity of a specific biological assay, we used the midpoint to calculate the corrected point estimate.

Corrected prevalence = (Observed prevalence + Specificity – 1) / (Sensitivity + Specificity – 1)

Text S4. *Appraisal tool for cross-sectional studies (AXIS)*

	Question	Yes	No	Don't know/ Comment
Introduction				
1	Were the aims/objectives of the study clear?			
Methods				
2	Was the study design appropriate for the stated aim(s)?			
3	Was the sample size justified?			
4	Was the target/reference population clearly defined? (Is it clear who the research was about?)			
5	Was the sample frame taken from an appropriate population base so that it closely represented the target/reference population under investigation?			
6	Was the selection process likely to select subjects/participants that were representative of the target/reference population under investigation?			
7	Were measures undertaken to address and categorise non-responders?			
8	Were the risk factor and outcome variables measured appropriate to the aims of the study?			
9	Were the risk factor and outcome variables measured correctly using instruments/measurements that had been trialled, piloted or published previously?			
10	Is it clear what was used to determined statistical significance and/or precision estimates? (e.g. p-values, confidence intervals)			
11	Were the methods (including statistical methods) sufficiently described to enable them to be repeated?			
Results				
12	Were the basic data adequately described?			
13	Does the response rate raise concerns about non-response bias?			
14	If appropriate, was information about non-responders described?			
15	Were the results internally consistent?			
16	Were the results presented for all the analyses described in the methods?			
Discussion				
17	Were the authors' discussions and conclusions justified by the results?			
18	Were the limitations of the study discussed?			
Other				
19	Were there any funding sources or conflicts of interest that may affect the authors' interpretation of the results?			
20	Was ethical approval or consent of participants attained?			

Text S5. References

1. Hambie EA, Larsen SA, Perryman MW, Pettit DE, Mullally RL, Whittington W. Comparison of a new rapid plasma reagin card test with the standard rapid plasma reagin 18-mm circle card test and the venereal disease research laboratory slide test for serodiagnosis of syphilis. *J Clin Microbiol* 1983; **17**(2): 249-54.
2. Perryman MW, Larsen SA, Hambie EA, Pettit DE, Mullally RL, Whittington W. Evaluation of a new rapid plasma reagin card test as a screening test for syphilis. *J Clin Microbiol* 1982; **16**(2): 286-90.
3. Parham CE, Pettit DE, Larsen SA, Hambie EA, Perryman MW, McGrew BE. Interlaboratory comparison of the toluidine red unheated serum test antigen preparation. *J Clin Microbiol* 1984; **20**(3): 434-7.
4. Cantor AG, Pappas M, Daeges M, Nelson HD. Screening for Syphilis: Updated Evidence Report and Systematic Review for the US Preventive Services Task Force. *JAMA* 2016; **315**(21): 2328-37.
5. Sena AC, White BL, Sparling PF. Novel Treponema pallidum serologic tests: a paradigm shift in syphilis screening for the 21st century. *Clin Infect Dis* 2010; **51**(6): 700-8.
6. Park IU, Fakile YF, Chow JM, et al. Performance of Treponemal Tests for the Diagnosis of Syphilis. *Clin Infect Dis* 2019; **68**(6): 913-8.
7. Tinajeros F, Grossman D, Richmond K, et al. Diagnostic accuracy of a point-of-care syphilis test when used among pregnant women in Bolivia. *Sex Transm Infect* 2006; **82 Suppl 5**: v17-21.
8. Nakku-Joloba E, Kiragga A, Mbazira JK, et al. Clinical Evaluation of 2 Point-of-Care Lateral Flow Tests for the Diagnosis of Syphilis. *Sex Transm Dis* 2016; **43**(10): 623-5.
9. Montoya PJ, Lukehart SA, Brentlinger PE, et al. Comparison of the diagnostic accuracy of a rapid immunochromatographic test and the rapid plasma reagin test for antenatal syphilis screening in Mozambique. *Bull World Health Organ* 2006; **84**(2): 97-104.
10. Matthias J, Dwiggins P, Totten Y, Blackmore C, Wilson C, Peterman TA. Notes from the Field: Evaluation of the Sensitivity and Specificity of a Commercially Available Rapid Syphilis Test - Escambia County, Florida, 2016. *MMWR Morb Mortal Wkly Rep* 2016; **65**(42): 1174-5.
11. Shakya G, Singh DR, Ojha HC, et al. Evaluation of SD Bioline HIV/syphilis Duo rapid test kits in Nepal. *BMC Infect Dis* 2016; **16**(1): 450.
12. Gayet-Ageron A, Lautenschlager S, Ninet B, Perneger TV, Combescure C. Sensitivity, specificity and likelihood ratios of PCR in the diagnosis of syphilis: a systematic review and meta-analysis. *Sex Transm Infect* 2013; **89**(3): 251-6.
13. HIV assays, laboratory performance and other operational characteristics, rapidd diagnostic tests (combined detection of HIV-1/2 antibodies and discriminatory detection of HIV-1 and HIV-2 antibodies), report 18. Geneva, Switzerland: World Health Organization, 2015. Available at: https://www.who.int/diagnostics_laboratory/evaluations/hiv/150819_hiv_assay_report18_final_version.pdf?ua=1 Accessed 1 March, 2020.
14. Bio-Rad. Genscreen ULTRA HIV Ag-Ab. Available at: http://www.bio-rad.com/webroot/web/pdf/inserts/CDG/en/883605_EN.pdf Accessed 1 March 2020.
15. Flores K, Leon SR. Diagnostic accuracy among 4 point-of-care tests for the screening of HIV infection. Lab Medicine 2013;44:172-175.
16. Jin F, Prestage GP, Zablotska I, et al. High rates of sexually transmitted infections in HIV positive homosexual men: Data from two community based cohorts. *Sexually Transmitted Infections* 2007; **83**(5): 397-9.
17. Lee D, Fairley C, Cummings R, Bush M, Read T, Chen M. Men who have sex with men prefer rapid testing for syphilis and may test more frequently using it. *Sexually Transmitted Diseases* 2010; **37**(9): 557-8.
18. Read PJ, Knight V, Bourne C, et al. Community event-based outreach screening for syphilis and other sexually transmissible infections among gay men in Sydney, Australia. *Sexual Health* 2013; **10**(4): 357-62.
19. Zou H, Prestage G, Fairley CK, et al. Sexual behaviors and risk for sexually transmitted infections among teenage men who have sex with men. *Journal of Adolescent Health*; **55**(2): 247-53.
20. Goddard SL, Poynten IM, Petoumenous K, et al. Prevalence, incidence and predictors of anal Chlamydia trachomatis, anal Neisseria gonorrhoeae and syphilis among older gay and bisexual men in the longitudinal Study for the Prevention of Anal Cancer (SPANC). *Sexually Transmitted Infections* 2019: 24.
21. Integrated biological & behavioral surveillance (IBBS) in selected cities of Afghanistan. Afghanistan: National AIDS Control Program (NACP), Ministry of Health, Afghanistan, 2012.
22. National HIV serological and behavioural surveillance, 2002, Bangladesh, fourth round technical report. Bangladesh: National AIDS/STD Programme, Directorate General of Services, Ministry of Health and Family Welfare, Government of the People's Republic of Bangladesh, 2002.
23. National HIV serological and behavioural surveillance, 2003-2004, Bangladesh, fifth round technical report. Bangladesh: National AIDS/STD Programme, Directorate General of Services, Ministry of Health and Family Welfare, Government of the People's Republic of Bangladesh, 2007.
24. National HIV serological surveillance, 2004-2005, Bangladesh, sixth round technical report. Bangladesh: National AIDS/STD Programme, Directorate General of Services, Ministry of Health and Family Welfare, Government of the People's Republic of Bangladesh, 2005.

25. National HIV serological surveillance, 2006, Bangladesh, seventh round technical report. Bangladesh: National AIDS/STD Programme, Directorate General of Services, Ministry of Health and Family Welfare, Government of the People's Republic of Bangladesh, 2007.
26. National HIV serological surveillance, 2007, Bangladesh, eighth round technical report. Bangladesh: National AIDS/STD Programme, Directorate General of Services, Ministry of Health and Family Welfare, Government of the People's Republic of Bangladesh, 2008.
27. National HIV serological surveillance, 2011, Bangladesh, ninth round technical report. Bangladesh: National AIDS/STD Programme, Directorate General of Services, Ministry of Health and Family Welfare, Government of the People's Republic of Bangladesh, 2011.
28. Rana AK, Reza MM, Alam MS, Khatun M, Khan SI, Azim T. Effects of In-country and Cross-Border Mobility on Condom Use Among Transgender Women (hijras) in Bangladesh: A Cross-Sectional Study. *AIDS and behavior* 2016; **20**(10): 2165-77.
29. Behavioural and serological surveillance on males having sex with males, male sex workers and hijra, 2015. Bangladesh: AIDS/STD Programme, Directorate General of Services, Ministry of Health and Family Welfare, Government of the People's Republic of Bangladesh, 2017.
30. Integrated biological and behavioral surveillance (IBBS) surveys among vulnerable and key populations at higher risk in Bhutan, 2016. Bhutan: School of Planning Monitoring Evaluation and Research (SPMER), 2016. .
31. Schneider JA, Lakshmi V, Dandona R, Kumar GA, Sudha T, Dandona L. Population-based seroprevalence of HSV-2 and syphilis in Andhra Pradesh state of India. *BMC Infectious Diseases* 2010; **10 (no pagination)**(59).
32. Solomon SS, Srikrishnan AK, Sifakis F, et al. The emerging HIV epidemic among men who have sex with men in Tamil Nadu, India: geographic diffusion and bisexual concurrency. *AIDS and behavior* 2010; **14**(5): 1001-10.
33. Ramakrishnan L, Ramanathan S, Chakrapani V, et al. Comparison of Sexual Risk, HIV/STI Prevalence and Intervention Exposure Among Men Who Have Sex with Men and Women (MSMW) and Men Who Have Sex with Men Only (MSMO) in India: Implications for HIV Prevention. *AIDS and behavior* 2015; **19**(12): 2255-69.
34. Solomon SS, Mehta SH, Srikrishnan AK, et al. High HIV prevalence and incidence among MSM across 12 cities in India. *Aids* 2015; **29**(6): 723-31.
35. Das A, George B, Ranebennur V, et al. Getting to the First 90: Incentivized Peer Mobilizers Promote HIV Testing Services to Men Who Have Sex With Men Using Social Media in Mumbai, India. *Global health, science and practice* 2019; **7**(3): 469-77.
36. Integrated bio-behavioral survey (IBBS) among MSM population in Kathmandu Valley. Nepal: Center for Research on Environment Health and Population Activities (CREHPA), 2005.
37. Integrated bio-behavioral survey (IBBS) among men who have sex with men in the Kathmandu Valley-2007. Nepal: New ERA, 2008.
38. Integrated biological and behavioral surveillance survey (IBBS) among men who have sex with men (MSM) in the Kathmandu Valley Round III-2009. Nepal: New ERA, 2009.
39. Integrated biological and behavioral surveillance (IBBS) survey among men who have sex with men (MSM) and transgender (TG) people in Kathmandu Valley, Nepal Round IV-2012. Kathmandu, Nepal: Ministry of Health, National Centre for AIDS and STD Control, 2013.
40. Integrated biological and behavioral surveillance survey (IBBS) among men who have sex with men (MSM) & transgender (TG) people in Kathmandu Valley, 2015, Round V. Kathmandu, Nepal: Ministry of Health, National Centre for AIDS and STD Control, 2015.
41. Integrated biological and behavioral surveillance (IBBS) survey among men who have sex with men (MSM) and transgender (TG) in Kathmandu Valley, Round VI. Kathmandu, Nepal: Ministry of Health, National Centre for AIDS and STD Control, 2017.
42. National study of reproductive tract and sexually transmitted infections, survey of high risk groups in Lahore and Karachi, 2005. Pakistan: National AIDS Control Program, Ministry of Health, Government of Pakistan, 2005.
43. Hawkes S, Collumbien M, Platt L, et al. HIV and other sexually transmitted infections among men, transgenders and women selling sex in two cities in Pakistan: A cross-sectional prevalence survey. *Sexually Transmitted Infections* 2009; **85**(SUPPL. 2): ii8-ii16.
44. Integrated biological and behavioural surveillance (IBBS) survey among key populations at higher risk of HIV in Sri Lanka. Sri Lanka: National STD/AIDS Control Programme (NSACP), 2015.
45. Integrated biological and behavioural surveillance (IBBS) survey among key populations at higher risk of HIV in Sri Lanka. Sri Lanka: National STD/AIDS Control Programme (NSACP), 2018.
46. Gulov K, Coulter RW, Matthews DD, Uzzi M, Stall R. HIV and STIs Among MSM in Tajikistan: Laboratory-Confirmed Diagnoses and Self-Reported Testing Behaviors. *AIDS and behavior* 2016; **20**(Supplement 3): 341-9.
47. Girault P, Saidel T, Song N, et al. HIV, STIs, and sexual behaviors among men who have sex with men in Phnom Penh, Cambodia. *AIDS Education and Prevention* 2004; **16**(1): 31-44.
48. Sopheab H, Morineau G, Neal JJ, et al. 2005 Cambodia STI prevalence survey, integrated biological and behavioral survey, sexually transmitted infections and related behaviors among brothel-based female sex workers, police, and men who have sex with men. Cambodia: National Center for HIV/AIDS, Dermatology and STDs, 2005.
49. Jiang J, Cao N, Zhang J, et al. High prevalence of sexually transmitted diseases among men who have sex with men in Jiangsu Province, China. *Sexually Transmitted Diseases* 2006; **33**(2): 118-23.

50. Ma X, Zhang Q, He X, et al. Trends in prevalence of HIV, syphilis, hepatitis C, hepatitis B, and sexual risk behavior among men who have sex with men: Results of 3 consecutive respondent-driven sampling surveys in Beijing, 2004 through 2006. *Journal of Acquired Immune Deficiency Syndromes* 2007; **45**(5): 581-7.
51. He Q, Wang Y, Lin P, et al. Potential bridges for HIV infection to men who have sex with men in Guangzhou, China. *AIDS & Behavior* 2006; **10**(4 Suppl): S17-23.
52. Choi KH, Ning Z, Gregorich SE, Pan QC. The influence of social and sexual networks in the spread of HIV and syphilis among men who have sex with men in Shanghai, China. *Journal of Acquired Immune Deficiency Syndromes* 2007; **45**(1): 77-84.
53. Ruan Y, Li D, Li X, et al. Relationship between syphilis and HIV infections among men who have sex with men in Beijing, China. *Sexually Transmitted Diseases* 2007; **34**(8): 592-7.
54. Zhang X, Wang C, Hengwei W, et al. Risk factors of HIV infection and prevalence of co-infections among men who have sex with men in Beijing, China. *Aids* 2007; **21**(SUPPL. 8): S53-S7.
55. Ye S, Xiao Y, Jin C, et al. Effectiveness of Integrated HIV Prevention Interventions among Chinese Men Who Have Sex with Men: Evaluation of a 16-City Public Health Program. *PLoS ONE* 2012; **7 (12) (no pagination)**(e50873).
56. Xiao Y, Sun J, Li C, et al. Prevalence and correlates of HIV and syphilis infections among men who have sex with men in seven Provinces in China with historically low HIV prevalence. *Journal of Acquired Immune Deficiency Syndromes* 2010; **53**(SUPPL. 1): S66-S73.
57. Zhang Y, Chen P, Lu R, et al. Prevalence of HIV among men who have sex with men in chongqing, China, 2006-2009: Cross-sectional biological and behavioural surveys. *Sexually Transmitted Infections* 2012; **88**(6): 444-50.
58. Zeng G, Feng L, Ouyang L, et al. The dynamic trends of HIV prevalence, risks, and prevention among men who have sex with men in Chongqing, China. *BioMed Research International* 2014; **2014 (no pagination)**(602719).
59. He Q, Wang Y, Lin P, et al. High prevalence of risk behaviour concurrent with links to other high-risk populations: A potentially explosive HIV epidemic among men who have sex with men in Guangzhou, China. *Sexually Transmitted Infections* 2009; **85**(5): 383-90.
60. Wang K, Yan H, Liu Y, Leng Z, Wang B, Zhao J. Increasing prevalence of HIV and syphilis but decreasing rate of self-reported unprotected anal intercourse among men who had sex with men in Harbin, China: Results of five consecutive surveys from 2006 to 2010. *International Journal of Epidemiology* 2012; **41**(2): 423-32.
61. Guo H, Wei JF, Yang H, Huan X, Tsui SKW, Zhang C. Rapidly increasing prevalence of HIV and syphilis and HIV-1 subtype characterization among men who have sex with men in Jiangsu, China. *Sexually Transmitted Diseases* 2009; **36**(2): 120-5.
62. Xu JJ, Zhang M, Brown K, et al. Syphilis and HIV seroconversion among a 12-month prospective cohort of men who have sex with men in Shenyang, China. *Sexually Transmitted Diseases* 2010; **37**(7): 432-9.
63. Feng TJ, Liu XL, Cai YM, et al. Prevalence of syphilis and human immunodeficiency virus infections among men who have sex with men in shenzhen, China: 2005 to 2007. *Sexually Transmitted Diseases* 2008; **35**(12): 1022-4.
64. Ruan Y, Luo F, Jia Y, et al. Risk factors for syphilis and prevalence of HIV, hepatitis B and C among men who have sex with men in Beijing, China: Implications for HIV prevention. *AIDS and Behavior* 2009; **13**(4): 663-70.
65. Zheng J, Wu Z, Poundstone KE, Pang L, Rou K. HIV, syphilis infection, and risky sexual behaviors among male university students who have sex with men in Beijing, China: A cross-sectional study. *AIDS Education and Prevention* 2012; **24**(1): 78-88.
66. Zou H, Wu Z, Yu J, et al. Sexual risk behaviors and HIV infection among men who have sex with men who use the internet in Beijing and Urumqi, China. *Journal of Acquired Immune Deficiency Syndromes* 2010; **53**(SUPPL. 1): S81-S7.
67. Feng Y, Wu Z, Detels R, et al. HIV/STD prevalence among men who have sex with men in Chengdu, China and associated risk factors for HIV infection. *Journal of Acquired Immune Deficiency Syndromes* 2010; **53**(SUPPL. 1): S74-S80.
68. Xiao Y, Ding X, Li C, Liu J, Sun J, Jia Y. Prevalence and correlates of hiv and syphilis infections among men who have sex with men in chongqing municipality, China. *Sexually Transmitted Diseases* 2009; **36**(10): 647-56.
69. Guo W, Wu ZY, Song AJ, Poundstone K. Impact of HIV/sexually transmitted infection testing on risky sexual behaviors among men who have sex with men in Langfang, China. *Chinese Medical Journal* 2013; **126**(7): 1257-63.
70. Zhu WM, Lin HJ, Zhang YF, et al. Human immunodeficiency virus/sexually transmitted infection, risk behavior and sexual networks among men who have sex with men in Taizhou city, Zhejiang province. [Chinese]. *Zhonghua liu xing bing xue za zhi = Zhonghua liuxingbingxue zazhi* 2008; **29**(10): 994-8.
71. Wang X, Lan G, Shen Z, et al. HIV and syphilis prevalence trends among men who have sex with men in Guangxi, China: Yearly cross-sectional surveys, 2008-2012. *BMC Infectious Diseases* 2014; **14 (1) (no pagination)**(367).
72. Li SM, Zhou ZH, Jiang SL, et al. [Incidence and risk factors of HIV and syphilis seroconversion among men who have sex with men in Beijing]. [Chinese]. *Zhonghua yu fang yi xue za zhi [Chinese journal of preventive medicine]* 2011; **45**(2): 118-22.
73. She M, Zhang H, Wang J, et al. Associated factors for HIV and syphilis infection among men who have sex with men only and men who have sex with both men and women in cities of China. *International Journal of STD and AIDS* 2013; **24**(4): 293-300.

74. Ma W, Wu G, Zheng H, et al. Prevalence and risk factors of HIV and syphilis, and knowledge and risk behaviors related to HIV/AIDS among men who have sex with men in Chongqing, China. *Journal of Biomedical Research* 2016; **30**(2): 101-11.
75. Zhong F, Liang B, Xu H, et al. Increasing HIV and decreasing syphilis prevalence in a context of persistently high unprotected anal intercourse, six consecutive annual surveys among men who have sex with men in Guangzhou, China, 2008 to 2013. *PLoS ONE* 2014; **9 (7) (no pagination)**(e103136).
76. Yan H, Li J, Raymond HF, et al. Increased HIV testing among men who have sex with men from 2008 to 2012, Nanjing, China. *PLoS ONE* 2016; **11 (4) (no pagination)**(e0154466).
77. Cai WD, Zhao J, Zhao JK, et al. HIV prevalence and related risk factors among male sex workers in Shenzhen, China: Results from a time-location sampling survey. *Sexually Transmitted Infections* 2010; **86**(1): 15-20.
78. Bai H, Huan X, Tang W, et al. A survey of HIV infection and related high-risk factors among men who have sex with men in Suzhou, Jiangsu, China. *Journal of Biomedical Research* 2011; **25**(1): 17-24.
79. Xu JJ, Reilly KH, Lu CM, et al. A cross-sectional study of HIV and syphilis infections among male students who have sex with men (MSM) in northeast China: implications for implementing HIV screening and intervention programs. *BMC public health* 2011; **11**: 287.
80. Wu Z, Liu E, Mao Y, et al. HIV and syphilis prevalence among men who have sex with men: A cross-sectional survey of 61 cities in China. *Clinical Infectious Diseases* 2013; **57**(2): 298-309.
81. Chen L, Mahapatra T, Fu G, et al. Male clients of male sex workers in China: An ignored high-risk population. *Journal of Acquired Immune Deficiency Syndromes* 2016; **71**(3): 316-22.
82. Cao Z, Xu J, Zhang H, et al. Risk factors for syphilis among married men who have sex with men in China. *Sexually Transmitted Diseases* 2014; **41**(2): 98-102.
83. Gao W, Li Z, Li Y, Qiao X. Sexual practices and the prevalence of HIV and syphilis among men who have sex with men in Lanzhou, China. *Japanese Journal of Infectious Diseases* 2015; **68**(5): 370-5.
84. Zhao J, Chen L, Cai WD, et al. HIV infection and sexual behaviors among non-commercial men who have sex with men at different venues. *Archives of sexual behavior* 2014; **43**(4): 801-9.
85. Liao M, Kang D, Jiang B, et al. Bisexual behavior and infection with HIV and syphilis among men who have sex with men along the east coast of China. *AIDS Patient Care and STDs* 2011; **25**(11): 683-91.
86. Qin Q, Tang W, Ge L, et al. Changing trend of HIV, Syphilis and Hepatitis C among Men Who Have Sex with Men in China. *Scientific reports* 2016; **6**: 31081.
87. Fan S, Lu H, Ma X, et al. Behavioral and serologic survey of men who have sex with Men in Beijing, China: Implication for HIV intervention. *AIDS Patient Care and STDs* 2012; **26**(3): 148-55.
88. Mao H, Ma W, Lu H, et al. High incidence of HIV and syphilis among migrant men who have sex with men in Beijing, China: A prospective cohort study. *BMJ Open* 2014; **4 (9) (no pagination)**(e005351).
89. Li D, Li S, Liu Y, et al. HIV incidence among men who have sex with men in Beijing: A prospective cohort study. *BMJ Open* 2012; **2 (6) (no pagination)**(e001829).
90. Chen X, Li X, Zheng J, et al. Club drugs and HIV/STD infection: An exploratory analysis among men who have sex with men in Changsha, China. *PLoS ONE* 2015; **10 (5) (no pagination)**(e0126320).
91. Zhang L, Ding X, Lu R, et al. Predictors of hiv and syphilis among men who have sex with men in a chinese metropolitan city: Comparison of risks among students and non-students. *PLoS ONE* 2012; **7 (5) (no pagination)**(e37211).
92. Yang HT, Tang W, Xiao ZP, et al. Worsening epidemic of HIV and syphilis among men who have sex with men in Jiangsu Province, China. *Clinical Infectious Diseases* 2014; **58**(12): 1753-9.
93. Jia Z, Huang X, Wu H, et al. HIV burden in men who have sex with men: a prospective cohort study 2007-2012. *Scientific reports* 2015; **5**: 11205.
94. Li D, Yang X, Zhang Z, et al. Incidence of Co-infections of HIV, herpes simplex virus type 2 and syphilis in a large cohort of men who have sex with men in Beijing, China. *PLoS ONE* 2016; **11 (1) (no pagination)**(e0147422).
95. Wang QQ, Chen XS, Yin YP, et al. HIV prevalence, incidence and risk behaviours among men who have sex with men in Yangzhou and Guangzhou, China: A cohort study. *Journal of the International AIDS Society* 2014; **17 (no pagination)**(18849).
96. Huan XP, Yin YP, Fu GF, et al. [Analysis on sexually transmitted diseases and the related risk factors among men who have sex with men in Jiangsu province]. [Chinese]. *Zhonghua yu fang yi xue za zhi [Chinese journal of preventive medicine]* 2011; **45**(11): 975-8.
97. Luo HB, Mei JY, Song LJ, et al. A comprehensive surveillance on HIV among 1,237 men who have sex with men in thirteen cities of Yunnan. [Chinese]. *Zhonghua liu xing bing xue za zhi = Zhonghua liuxingbingxue zazhi* 2012; **33**(7): 706-9.
98. Chen Y, Tang W, Chen L, et al. Changing Epidemic of HIV and Syphilis Among Resident and Migrant Men Who Have Sex with Men in Jiangsu, China. *Scientific reports* 2017; **7**(1): 9478.
99. Hu J, Gu X, Tao X, et al. Prevalence and trends of HIV, syphilis, and HCV in migrant and resident men who have sex with men in Shandong, China: Results from a serial cross-sectional study. *PLoS ONE* 2017; **12 (1) (no pagination)**(e0170443).
100. Zhang X, Yu J, Sun X, et al. Prevalence and related risk behaviors of HIV, syphilis, and anal HPV infection among men who have sex with men from Beijing, China. *AIDS and behavior* 2013; **17**(3): 1129-36.

101. Wu J, Wu H, Li P, Lu C. HIV/STIs risks between migrant MSM and local MSM: A cross-sectional comparison study in China. *PeerJ* 2016; **2016 (7) (no pagination)**(e2169).
102. Xu J, An M, Han X, et al. Prospective cohort study of HIV incidence and molecular characteristics of HIV among men who have sex with men(MSM) in Yunnan Province, China. *BMC Infectious Diseases* 2013; **13**: 3.
103. Zhao J, Cai R, Chen L, et al. A comparison between respondent-driven sampling and time-location sampling among men who have sex with men in Shenzhen, China. *Archives of sexual behavior* 2015; **44**(7): 2055-65.
104. Zhou H, Zhao C, Liu X, et al. The analysis of AIDS monitoring conditions among 400 MSMs in Shijiazhuang. J Med Pest Control 2011; 27(11): 1007-9.
105. Liu J, Qu B, Ezeakile MC, Zhang Y, Liang S. Factors associated with HIV infection among men who have sex with men in Henan Province, China: a cross-sectional study. *BMC public health* 2013; **13**: 356.
106. Tao J, Ruan Y, Yin L, et al. Sex with women among men who have sex with men in China: Prevalence and sexual practices. *AIDS Patient Care and STDs* 2013; **27**(9): 524-8.
107. Fan W, Yin L, Qian HZ, et al. HIV risk perception among HIV negative or status-unknown men who have sex with men in China. *BioMed Research International* 2014; **2014 (no pagination)**(232451).
108. Zhang H, Lu H, Pan SW, et al. Correlates of unprotected anal intercourse: the influence of anal sex position among men who have sex with men in Beijing, china. *Arch Sex Behav* 2015; **44**(2): 375-87.
109. Huang D, Hu Y, Wu G, et al. HIV prevention services and testing utilization behaviors among men who have sex with men at elevated risk for HIV in Chongqing, china. *BioMed Research International* 2014; **2014 (no pagination)**(174870).
110. Cai Y, Liu H, Song Y, Hong F. Factors associated with HIV and syphilis infection among men who have sex with men blood donors in Shenzhen. [Chinese]. *Zhonghua yu fang yi xue za zhi [Chinese journal of preventive medicine]* 2014; **48**(2): 128-32.
111. Wang L, Norris JL, Li DM, Guo W, Ding ZW, Wang N. HIV prevalence and influencing factors analysis of sentinel surveillance among men who have sex with men in China, 2003 - 2011. *Chinese medical journal* 2012; **125**(11): 1857-61.
112. Qi J, Zhang D, Fu X, et al. High risks of HIV transmission for men who have sex with men - A comparison of risk factors of HIV infection among MSM associated with recruitment channels in 15 cities of China. *PLoS ONE* 2015; **10 (4) (no pagination)**(e0121267).
113. LAN G, Chen Y, Zhang H, Zhu Q. HIV prevalence and risk factors among MSM in Bathrooms in Guangxi. J Prev Med Inf 2014; 30(2): 87-9.
114. Hu H, Liu X, Zhang Z, et al. Increasing HIV Incidence among Men Who Have Sex with Men in Jiangsu Province, China: Results from Five Consecutive Surveys, 2011-2015. *International Journal of Environmental Research & Public Health [Electronic Resource]* 2016; **13**(8): 06.
115. Wang N, Wu G, Lu R, et al. Investigating HIV Infection and HIV Incidence Among Chinese Men Who Have Sex with Men with Recent Sexual Debut, Chongqing, China, 2011. *AIDS and behavior* 2016; **20**(12): 2976-82.
116. Luo Y, Zhu C, Chen S, et al. Risk factors for HIV and syphilis infection among male sex workers who have sex with men: a cross-sectional study in Hangzhou, China, 2011. *BMJ open* 2015; **5**(4): e006791.
117. Zhang L, Zhang D, Yu B, et al. Prevalence of HIV Infection and Associated Risk Factors among Men Who Have Sex with Men (MSM) in Harbin, P. R. China. *PLoS ONE* 2013; **8 (3) (no pagination)**(e58440).
118. Liao M, Kang D, Tao X, et al. Alcohol use, stigmatizing/discriminatory attitudes, and HIV high-risk sexual behaviors among men who have sex with men in China. *Biomed Res Int* 2014; **2014**: 143738.
119. Wu ZL, Guan GY, Zhao JH, et al. Dynamic Characteristics and HIV Infection of Men who have Sex with Men from 2011 to 2017 in Yinchuan, Ningxia, China. *Current HIV research* 2018; **16**(5): 364-73.
120. Fan W, Lu R, Wu G, et al. Alcohol drinking and HIV-related risk among men who have sex with men in Chongqing, China. *Alcohol* 2016; **50**: 1-7.
121. Pan S, Xu JJ, Han XX, et al. Internet-Based Sex-Seeking Behavior Promotes HIV Infection Risk: A 6-Year Serial Cross-Sectional Survey to MSM in Shenyang, China. *BioMed Research International* 2016; **2016 (no pagination)**(2860346).
122. Qu L, Wang W, Gao Y, et al. A Cross-sectional Survey of HIV Transmission and Behavior among Men Who Have Sex with Men in Different Areas of Inner Mongolia Autonomous Region, China. *BMC public health* 2016; **16**(1): 1161.
123. Liang L, Zhang J, Liu L, Luo Y, Pei X, Hu Y. Infection status of HIV and its influence factors among men who have sex with men in Sichuan province. [Chinese]. *Zhonghua yu fang yi xue za zhi [Chinese journal of preventive medicine]* 2014; **48**(11): 980-4.
124. Chu ZX, Xu JJ, Zhang YH, et al. Poppers use and Sexual Partner Concurrency Increase the HIV Incidence of MSM: a 24-month Prospective Cohort Survey in Shenyang, China. *Scientific reports* 2018; **8**(1): 24.
125. Mao X, Hu Q, Huang C, et al. HIV incidence is rapidly increasing with age among young men who have sex with men in China: a multicentre cross-sectional survey. *HIV Medicine* 2018; **19**(8): 513-22.
126. Xu JJ, Zhang C, Hu QH, et al. Recreational drug use and risks of HIV and sexually transmitted infections among Chinese men who have sex with men: Mediation through multiple sexual partnerships. *BMC Infectious Diseases* 2014; **14 (1) (no pagination)**(642).

127. Guanghua L, Yi C, Shuai T, et al. HIV, syphilis and behavioral risk factors among men who have sex with men in a drug-using area of southwestern China: Results of 3 cross-sectional surveys from 2013 to 2015. *Medicine* 2018; **97**(16): e0404.
128. Zhang Y, Wu G, Lu R, et al. What has changed HIV and syphilis infection among men who have sex with men (MSM) in Southwest China: a comparison of prevalence and behavioural characteristics (2013-2017). *BMC public health* 2019; **19**(1): 1314.
129. Wu H, Xiu C, Fu X, et al. Syphilis associated with recreational drug use, depression and high-risk sexual behaviour in men who have sex with men: A case-control study in China. *Sexually Transmitted Infections* 2019.
130. Ding W, Zhang Y. Survery on knowledge, attitude and practice about HIV/syphilis among MSM, Qingzhou city, 2013. Prev Med Trib 2014; 20(2): 112-4.
131. Zhang J, Xu JJ, Song W, et al. HIV Incidence and Care Linkage among MSM First-Time-Testers in Shenyang, China 2012-2014. *AIDS & Behavior* 2018; **22**(3): 711-21.
132. Cai YM, Song YJ, Liu H, Hong FC. Factors associated with commercial sexual behavior among men who have sex with men in Shenzhen, China, in 2011-2015. [Chinese]. *Zhonghua yu fang yi xue za zhi [Chinese journal of preventive medicine]* 2016; **50**(11): 943-8.
133. Liu Y, Li D, Vermund SH, et al. Associations of current marital status and living arrangements with HIV and syphilis risk: findings from a community-based sample of men who have sex with men in China. *AIDS Care - Psychological and Socio-Medical Aspects of AIDS/HIV* 2016; **28**(11): 1461-6.
134. Li R, Pan X, Ma Q, et al. Prevalence of prior HIV testing and associated factors among MSM in Zhejiang Province, China: a cross-sectional study. *BMC Public Health* 2016; **16**(1): 1152.
135. Liu YY, Tao HD, Liu J, et al. Prevalence and associated factors of HIV infection among men who have sex with men in Hefei, China, 2013-2014: a cross-sectional study. *International Journal of STD and AIDS* 2016; **27**(4): 305-12.
136. Huang Y, Zhang Y, Li K, Zhao J. Changes in prevalence of HIV or syphilis among male sex workers and non-commercial men who have sex with men in Shenzhen, China: Results of a second survey. *PLoS ONE* 2016; **11 (12) (no pagination)**(e0167619).
137. Li Q, Li X, Luo Y, et al. HIV incidence and cohort retention among men who have sex with men in Hangzhou, China: A prospective cohort study. *Medicine (United States)* 2019; **98 (40) (no pagination)**(e17419).
138. Zhang X, Jia M, Chen M, et al. Prevalence and the associated risk factors of HIV, STIs and HBV among men who have sex with men in Kunming, China. *International Journal of STD and AIDS* 2017; **28**(11): 1115-23.
139. Dorjiwangmo, Lu S, Iasi, et al. Epidemiological survey of prevalence of HIV infection, syphilis and hepatitis C in female sex workers and other 6 risk populations in Tibet Autonomous Region. [Chinese]. *Chinese Journal of Endemiology* 2017; **38**(7): 921-5.
140. Liu Y, Tang H, Ning Z, Zheng H, He N, Zhang Y. Co-infections of HIV, syphilis and HSV-2 among men who have sex with men at the voluntary IIIV counseling and testing clinics in Shanghai. [Chinese]. *Chinese Journal of Endemiology* 2017; **38**(10): 1363-6.
141. An X, Sun Q, Fang F, et al. The Prevalence of Depression Associated with the Infection Status and Sexual Behaviors among Men Who Have Sex with Men in Shenzhen, China: A Cross-Sectional Study. *International Journal of Environmental Research & Public Health [Electronic Resource]* 2019; **17**(1): 23.
142. Wei L, Chen L, Zhang H, et al. Use of gay app and the associated HIV/syphilis risk among non-commercial men who have sex with men in Shenzhen, China: A serial cross-sectional study. *Sexually Transmitted Infections* 2019.
143. Bai JY, Ning TL, Zhou N, Guo Y, Yu MH. [HIV infection status and related factors in men who have sex with men in sentinel surveillance in Tianjin, 2016-2018][Chinese]. *Chung-Hua Liu Hsing Ping Hsueh Tsa Chih Chinese Journal of Epidemiology*; **40**(9): 1106-10.
144. Liao M, Liu D, Zhu X, et al. Behavior and influencing factors of HIV infection among men who have sex with men only and men who have sex with both men and women in Shandong province. [Chinese]. *Chinese Journal of Endemiology* 2019; **40**(8): 941-6.
145. Pisani E, Girault P, Gultom M, et al. HIV, syphilis infection, and sexual practices among transgenders, male sex workers, and other men who have sex with men in Jakarta, Indonesia. *Sexually Transmitted Infections* 2004; **80**(6): 536-40.
146. Technical Report: 2013 sero-surveillance survey and 2013 rapid behavioral survey. Indonesia: Ministry of Health, National AIDS Commision, 2013.
147. Itoda I, Hoshino S, Sawada T, et al. [Prevalence of HIV and sexually transmitted infections and characteristics of men who have sex with men at a community-based center in Yokohama, Japan]. [Japanese]. *[Nihon koshu eisei zasshi] Japanese journal of public health* 2013; **60**(5): 253-61.
148. Wickersham JA, Gibson BA, Bazazi AR, et al. Prevalence of Human Immunodeficiency Virus and Sexually Transmitted Infections among Cisgender and Transgender Women Sex Workers in Greater Kuala Lumpur, Malaysia: Results from a Respondent-Driven Sampling Study. *Sexually Transmitted Diseases* 2017; **44**(11): 663-70.
149. Davaalkham J, Unenchimeg P, Baigalmaa C, et al. High-risk status of HIV-1 infection in the very low epidemic country, Mongolia, 2007. *Int J STD AIDS* 2009; **20**(6): 391-4.
150. Second generation HIV and STI surveillance report, 2011. Mongolia: Ministry of Health, 2012.
151. HIV/STI surveillance survey report, 2014. Mongolia: Ministry of Health and Sports, 2014.
152. Report of the HIV sentinel sero-surveillance survey 2007 Myanmar. Myanmar: National AIDS Programme, Department of Health, Ministry of Health, 2007.

153. Report of the HIV sentinel sero-surveillance survey 2008 Myanmar. Myanmar: National AIDS Programme, Department of Health, Ministry of Health, 2009.
154. Results of HIV sentinel sero-surveillance 2009 Myanmar. Myanmar: National AIDS Programme, Department of Health, Ministry of Health, 2010.
155. Results of HIV sentinel sero-surveillance 2010 Myanmar. Myanmar: National AIDS Programme, Department of Health, Ministry of Health, 2011.
156. Results of HIV sentinel sero-surveillance 2011 Myanmar. Myanmar: National AIDS Programme, Department of Health, Ministry of Health, 2012.
157. HIV sentinel sero-surveillance survey report, 2012. Myanmar: National AIDS Programme, Department of Health, Ministry of Health, 2013.
158. 2013 Integrated HIV behavioral and serologic surveillance (IHBSS) among higher risk transgender women in Cebu city, Philippines. Philippines: National Epidemiology Center, Department of Health, 2014.
159. Jung M, Lee J, Kwon DS, Park BJ. Comparison of sexual risky factors of men who have sex with men and sex-buying men as groups vulnerable to sexually transmitted diseases. *Journal of Preventive Medicine and Public Health* 2012; **45**(3): 156-63.
160. Ananworanich J, Chitwarakorn A, Wimonsate W, et al. HIV and syphilis infection among men who have sex with men - Bangkok, Thailand, 2005-2011. *Morbidity and Mortality Weekly Report* 2013; **62**(25): 518-20.
161. Holtz TH, Thienkrua W, McNicholl JM, et al. Prevalence of Treponema pallidum seropositivity and herpes simplex virus type 2 infection in a cohort of men who have sex with men, Bangkok, Thailand, 2006-2010. *International Journal of STD and AIDS* 2012; **23**(6): 424-8.
162. Van Griensven F, Thienkrua W, McNicholl J, et al. Evidence of an explosive epidemic of HIV infection in a cohort of men who have sex with men in Thailand. *Aids* 2013; **27**(5): 825-32.
163. Phanuphak N, Sungsing T, Jantarapakde J, et al. Princess PrEP program: The first key population-led model to deliver pre-exposure prophylaxis to key populations by key populations in Thailand. *Sexual Health* 2018; **15**(6): 542-55.
164. Pisani E, Purnomo H, Sutrisna A, et al. Basing policy on evidence: Low HIV, STIs, and risk behaviour in Dili, East Timor argue for more focused interventions. *Sexually Transmitted Infections* 2006; **82**(1): 88-93.
165. Integrated biological-behavioral surveillance surveys among female sex workers and men who have sex with men in Timor-Leste, 2016-2017. Timor-Leste: Australasian Society for HIV, Viral Hepatitis and Sexual Health Medicine (ASHM), 2017.
166. Pham QD, Nguyen TV, Hoang CQ, et al. Prevalence of HIV/STIs and associated factors among men who have sex with men in An Giang, Vietnam. *Sexually Transmitted Diseases* 2012; **39**(10): 799-806.
167. Results from the HIV/STI integrated biological and behavioral surveillance (IBBS) in Vietnam - Round II 2009. Vietnam: Ministry of Health, Vietnam, 2011.
168. Nguyen TV, Van Khuu N, Nguyen PD, et al. Sociodemographic Factors, Sexual Behaviors, and Alcohol and Recreational Drug Use Associated with HIV Among Men Who Have Sex with Men in Southern Vietnam. *AIDS and behavior* 2016; **20**(10): 2357-71.
169. Colby D, Nguyen NA, Le B, et al. HIV and Syphilis Prevalence Among Transgender Women in Ho Chi Minh City, Vietnam. *AIDS and behavior* 2016; **20**(Supplement 3): 379-85.
170. Reyniers T, Nostlinger C, Laga M, et al. Choosing Between Daily and Event-Driven Pre-exposure Prophylaxis: Results of a Belgian PrEP Demonstration Project. *J Acquir Immune Defic Syndr* 2018; **79**(2): 186-94.
171. Ravlija J, Jandric L, Zeljko M, Kurtovic A. Research of risk behaviour with regard to HIV prevalence in groups at increased risk. Sarajevo, Bosnia-Herzegovina: Partnerships in Health, 2011.
172. Ravlija J, Jandric L, Vilic-Svraka A, Stojisavljevic S. Research on risk behaviour in relation to HIV/STI prevalence among groups exposed to higher risk (MSM and SW) in Bosnia and Herzegovina, 2012. Bosnia and Herzegovina: 2012.
173. Nelson LE, Tharao W, Husbands W, et al. The epidemiology of HIV and other sexually transmitted infections in African, Caribbean and Black men in Toronto, Canada. *BMC Infectious Diseases* 2019; **19 (1) (no pagination)**(294).
174. Bozicevic I, Rode OD, Lepej SZ, et al. Prevalence of sexually transmitted infections among men who have sex with men in Zagreb, Croatia. *AIDS and Behavior* 2009; **13**(2): 303-9.
175. Bozicevic I, Lepej SZ, Rode OD, et al. Prevalence of HIV and sexually transmitted infections and patterns of recent HIV testing among men who have sex with men in Zagreb, Croatia. *Sexually Transmitted Infections* 2012; **88**(7): 539-44.
176. Vilibic-Cavlek T, Kolaric B, Pavlic J, Kosanovic-Licina ML, Nemeth-Blazic T. Seroprevalence and risk factors for HIV, hepatitis B, and syphilis in populations with high-risk behaviors in Croatia. *Acta Dermatovenerologica Croatica* 2018; **26**(4): 314-20.
177. Ruutel K, Lohmus L, Janes J. Internet-based recruitment system for HIV and STI screening for men who have sex with men in Estonia, 2013: Analysis of preliminary outcomes. *Eurosurveillance* 2015; **20 (15) (no pagination)**(21094).
178. Zorzi A, Cordioli M, Gios L, et al. Field evaluation of two point-of-care tests for syphilis among men who have sex with men, Verona, Italy. *Sexually transmitted infections* 2017; **93**(S4): S51-S8.
179. Mikik V, Kuzmanovska G, Memeti S. Report from the bio behavioural survey and assessment of population size of man having sex with man in Macedonia, 2010. Institute for Public Health of Republic of Macedonia, Ministry of Health of Republic of Macedonia, 2011.

180. Report on the bio-behavioral study among "men having sex with men" pupulation in Skopje, Macedonia 2013-2014. Skopje, Macedonia: Ministry of Health of Republic of Macedonia, 2014.
181. HIV sentinel suerveillance in high-risk groups in Azerbaijan, the Republic of Moldova and the Russian Federation. Copenhagen Denmark: Regional Office for Europe of the World Health Organization, 2004.
182. [Studiul comportamental și de seroprevalenţă Moldova 2007].[Romanian]. Moldova: National Center of Health Management, 2008.
183. Zohrabyan L, Johnston L, Scutelniciuc O, et al. HIV, hepatitis and syphilis prevalence and correlates of condom use during anal sex among men who have sex with men in the Republic of Moldova. *International Journal of STD and AIDS* 2013; **24**(5): 357-64.
184. Hoornenborg E, Achterbergh RCA, van der Loeff MFS, et al. Men who have sex with men more often chose daily than event-driven use of pre-exposure prophylaxis: Baseline analysis of a demonstration study in Amsterdam. *Journal of the International AIDS Society* 2018; **21 (3) (no pagination)**(e25105).
185. Amirkhanian YA, Kelly JA, Kirsanova AV, et al. HIV risk behaviour patterns, predictors, and sexually transmitted disease prevalence in the social networks of young men who have sex with men in St Petersburg, Russia. *International Journal of STD and AIDS* 2006; **17**(1): 50-6.
186. Baral S, Kizub D, Masenior NF, et al. Male sex workers in Moscow, Russia: A pilot study of demographics, substance use patterns, and prevalence of HIV-1 and sexually transmitted infections. *AIDS Care - Psychological and Socio-Medical Aspects of AIDS/HIV* 2010; **22**(1): 112-8.
187. HIV prevalence and risks among men having sex with men in Moscow and Saint Petersburg. Copenhagen Denmark: Regional Office for Europe of the World Health Organization, 2007.
188. Wirtz AL, Zelaya CE, Peryshkina A, et al. Anal human papillomavirus and HIV: A cross-sectional study among men who have sex with men in Moscow, Russia, 2012-2013. *Eurosurveillance* 2015; **20 (15) (no pagination)**(21095).
189. [Research among populations most at risk to HIV].[Serbian]. Serbia: Ministry of Health, 2012.
190. HIV integrated behavioral and biological surveillance surveys-Kosovo, 2014, people who inject drugs in Prishtina and Prizren, men who have sex with men in Prishtina. Kosovo: Kosovo survey reference group, 2014.
191. Hurtado I, Alastrue I, Pavlou M, Tasa T, Perez-Hoyos S. Increased syphilis trend among patients in an AIDS information and prevention center. *Gaceta Sanitaria* 2011; **25**(5): 368-71.
192. Hurtado I, Alastrue I, Garcia de Olalla P, Albiach D, Martin M, Perez-Hoyos S. Preventive intervention in venues for interaction used by men who have sex with men. [Spanish]. *Gaceta Sanitaria* 2010; **24**(1): 78-80.
193. Borelli S, Monn A, Meyer J, Berger U, Honegger HP, Lautenschlager S. Evaluation of a particle gel immunoassay as a screening test for syphilis. *Infection* 2009; **37**(1): 26-8.
194. Manavi K, Williams G, Newton R. The uptake of HIV and syphilis testing in a nurse-delivered service during Gay Pride events. *International Journal of STD & AIDS* 2012; **23**(12): 887-9.
195. Golden MR, Gift TL, Brewer DD, et al. Peer referral for HIV case-finding among men who have sex with men. *AIDS* 2006; **20**(15): 1961-8.
196. Mayer KH, Ducharme R, Zaller N, et al. Unprotected sex, underestimated risk, undiagnosed HIV and sexually transmitted diseases among men who have sex with men accessing testing services in a new england bathhouse. []. *JAIDS Journal of Acquired Immune Deficiency Syndromes* 2011.
197. Katz DA, Golden MR, Hughes JP, Farquhar C, Stekler JD. HIV Self-testing increases HIV testing frequency in high-risk men who have sex with men: A randomized controlled trial. *Journal of Acquired Immune Deficiency Syndromes* 2018; **78**(5): 505-12.
198. Watson DL, Liao C, Ridgway J, Schneider JA. Behavioral characteristics and their association with syphilis seroprevalence among a cohort of young Black men who have sex with men. *International Journal of STD and AIDS* 2019; **30**(4): 396-403.
199. Fujimoto K, Flash CA, Kuhns LM, Kim JY, Schneider JA. Social networks as drivers of syphilis and HIV infection among young men who have sex with men. *Sexually Transmitted Infections* 2018; **94**(5): 365-71.
200. Reback CJ, Fletcher JB. Elevated HIV and STI prevalence and incidence among methamphetamine-using men who have sex with men in Los Angeles county. *AIDS Education and Prevention* 2018; **30**(4): 350-6.
201. Obafemi OA, Wendel KA, Anderson TS, et al. Rapid Syphilis Testing for Men Who Have Sex with Men in Outreach Settings: Evaluation of Test Performance and Impact on Time to Treatment. *Sexually Transmitted Diseases* 2019; **46**(3): 191-5.
202. Shannon CL, Keizur EM, Fehrenbacher A, et al. Sexually Transmitted Infection Positivity among Adolescents with or at High-Risk for Human Immunodeficiency Virus Infection in Los Angeles and New Orleans. *Sexually Transmitted Diseases* 2019; **46**(11): 737-42.
203. Pando MA, Bautista CT, Maulen S, et al. Epidemiology of human immunodeficiency virus, viral hepatitis (B and C), Treponema pallidum, and human T-cell lymphotropic I/II virus among men who have sex with men in Buenos Aires, Argentina. *Sexually Transmitted Diseases* 2006; **33**(5): 307-13.
204. Berini CA, Pando MA, Bautista CT, et al. HTLV-1/2 among high-risk groups in Argentina: Molecular diagnosis and prevalence of different sexual transmitted infections. *Journal of Medical Virology* 2007; **79**(12): 1914-20.
205. Segura M, Bautista CT, Marone R, et al. HIV/STI co-infections, syphilis incidence, and hepatitis B vaccination: The Buenos Aires cohort of men who have sex with men. *AIDS Care - Psychological and Socio-Medical Aspects of AIDS/HIV* 2010; **22**(12): 1459-65.

206. Avila MM, Dos Ramos Farias MS, Fazzi L, et al. High Frequency of Illegal Drug Use Influences Condom Use Among Female Transgender Sex Workers in Argentina: Impact on HIV and Syphilis Infections. *AIDS and behavior* 2017; **21**(7): 2059-68.
207. Pando MA, Balan IC, Marone R, et al. HIV and other sexually transmitted infections among men who have sex with men recruited by RDS in Buenos Aires, Argentina: High HIV and HPV infection. *PLoS ONE* 2012; **7 (6) (no pagination)**(e39834).
208. Central Americans behavioural seroprevalence survey of HIV and other STIs in more vulnerable populations: sex workers, men who have sex with men and persons with HIV, Belize 2011-2012. Belize: National TB, HIV & other STIs Programme, Ministry of Health of Belize, 2012.
209. [Sexualidad masculine y VIH en Bolivia (SEMVBO)].[Spanish]. Bolivia: Ministry of Health and Sports, 2010.
210. Soares CC, Georg I, Lampe E, et al. HIV-1, HBV, HCV, HTLV, HPV-16/18, and Treponema pallidum infections in a sample of Brazilian men who have sex with men. *PLoS ONE* 2014; **9 (8) (no pagination)**(e102676).
211. Ribeiro D, Rezende EF, Pinto VM, Pereira GFM, Miranda AE. Prevalence of and risk factors for syphilis in Brazilian armed forces conscripts. *Sexually Transmitted Infections* 2012; **88**(1): 32-4.
212. Brignol S, Dourado I, Amorim LD, Kerr LR. Vulnerability in the context of HIV and syphilis infection in a population of men who have sex with men (MSM) in Salvador, Bahia State, Brazil. *Cadernos de saude publica* 2015; **31**(5): 1035-48.
213. de Albuquerque AC, da Silva DM, Rabelo DC, et al. [Seroprevalence and factors associated with human immunodeficiency virus (HIV) and syphilis in inmates in the state of Pernambuco, Brazil][Portuguese]. *Ciencia & Saude Coletiva*; **19**(7): 2125-32.
214. Fernandes FRP, Zanini PB, Rezende GR, et al. Syphilis infection, sexual practices and bisexual behaviour among men who have sex with men and transgender women: A cross-sectional study. *Sexually Transmitted Infections* 2015; **91**(2): 142-9.
215. Hoagland B, Moreira RI, De Boni RB, et al. High pre-exposure prophylaxis uptake and early adherence among men who have sex with men and transgender women at risk for HIV Infection: The PrEP Brasil demonstration project. *Journal of the International AIDS Society* 2017; **20 (1) (no pagination)**(21472).
216. Grinsztejn B, Jalil EM, Monteiro L, et al. Unveiling of HIV dynamics among transgender women: a respondent-driven sampling study in Rio de Janeiro, Brazil. *The Lancet HIV* 2017; **4**(4): e169-e76.
217. Da Motta LR, Dea Sperhacke R, De Gregori Adami A, et al. Syphilis prevalence and risk factors among young men presenting to the Brazilian Army in 2016 Results from a national survey. *Medicine (United States)* 2018; **97 (47) (no pagination)**(e13309).
218. Ferreira ACG, Coelho LE, Jalil EM, et al. Transcendendo: A Cohort Study of HIV-Infected and Uninfected Transgender Women in Rio de Janeiro, Brazil. *Transgender Health* 2019; **4**(1): 107-17.
219. [Prevalencia de VIH, sifilis y comportamiento de riesgo en hombres que tienen sexo con hombres del gran area metropolitana de Costa Rica].[Spanish]. Costa Rica: Ministry of Health, 2010.
220. Brito MO, Hodge D, Donastorg Y, Khosla S, Lerebours L, Pope Z. Risk behaviours and prevalence of sexually transmitted infections and HIV in a group of Dominican gay men, other men who have sex with men and transgender women. *BMJ open* 2015; **5**(4): e007747.
221. Hernandez I, Johnson A, Reina-Ortiz M, et al. Syphilis and HIV/Syphilis Co-infection Among Men Who Have Sex With Men (MSM) in Ecuador. *American journal of men's health* 2017; **11**(4): 823-33.
222. Jacobson JO, Sanchez-Gomez A, Montoya O, et al. A continuing HIV epidemic and differential patterns of HIV-STI risk among MSM in Quito, Ecuador: an urgent need to scale up HIV testing and prevention. *AIDS and behavior* 2014; **18**(1): 88-98.
223. Sanchez-Gomez A, Jacobson JO, Montoya O, et al. HIV, STI and Behavioral Risk Among Men Who have Sex with Men in a Setting of Elevated HIV Prevalence Along Ecuador's Pacific Coast. *AIDS and behavior* 2015; **19**(9): 1609-18.
224. Ham C, Northbrook S, Morales-Miranda S, et al. HIV and STIs among transgendered populations: Four Country Survey from Central America. Poster of the Conference on Retroviruses and Opportunistic Infections, 2015 Feb 23-26; Seattle, Washington.
225. Creswell J, Guardado ME, Lee J, et al. HIV and STI control in El Salvador: Results from an integrated behavioural survey among men who have sex with men. *Sexually Transmitted Infections* 2012; **88**(8): 633-8.
226. Lahuerta M, Sabido M, Giardina F, et al. Comparison of users of an HIV/syphilis screening community-based mobile van and traditional voluntary counselling and testing sites in Guatemala. *Sexually Transmitted Infections* 2011; **87**(2): 136-40.
227. Guyana biobehavioral surveillance survey (BBSS). Guyana: National AIDS Programme Secretariat, Ministry of Health, 2014.
228. [HAITI VIH 2014: Surveillance biologique et comportementale utilisant la méthodologie respondent driven sampling (RDS) auprès des hommes ayant des relations sexuelles avec d'autres hommes (HARSAH)].[French]. Haiti: Organisation Haïtienne de Marketing Social pour la Santé, 2015.
229. Zalla LC, Herce ME, Edwards JK, Michel J, Weir SS. The burden of HIV among female sex workers, men who have sex with men and transgender women in Haiti: results from the 2016 Priorities for Local AIDS Control Efforts (PLACE) study. *Journal of the International AIDS Society* 2019; **22**(7): e25281.

230. Figueroa JP, Weir SS, Jones-Cooper C, et al. High HIV prevalence among men who have sex with men in Jamaica is associated with social vulnerability and other sexually transmitted infections. *West Indian Medical Journal* 2013; **62**(4): 286-91.
231. Logie CH, Wang Y, Lacombe-Duncan A, et al. Factors Associated with Syphilis Testing and a History of Syphilis Infection among a Sample of Transgender Women in Jamaica. *Sexually Transmitted Diseases* 2018; **45**(3): 158-62.
232. Pitpitan EV, Goodman-Meza D, Burgos JL, et al. Prevalence and correlates of HIV among men who have sex with men in Tijuana, Mexico. *Journal of the International AIDS Society* 2015; **18 (1) (no pagination)**(19304).
233. Martinez Vizcaino G, Belaunzaran-Zamudio P, Galicia Juarez V. Syphilis prevalence among men who have sex with men attending community-based HIV testing clinic in the city of Guadalajara, Mexico (2016 to 2018). *Journal of the International AIDS Society Conference: HIV and Hepatitis in the Americas* 2019; **22**(Supplement 2).
234. Hakre S, Arteaga GB, Nunez AE, et al. Prevalence of HIV, syphilis, and other sexually transmitted infections among MSM from three cities in Panama. *Journal of urban health : bulletin of the New York Academy of Medicine* 2014; **91**(4): 793-808.
235. Sanchez J, Lama JR, Kusunoki L, et al. HIV-1, sexually transmitted infections, and sexual behavior trends among men who have sex with men in Lima, Peru. *Journal of Acquired Immune Deficiency Syndromes* 2007; **44**(5): 578-85.
236. Clark JL, Caceres CF, Lescano AG, et al. Prevalence of same-sex sexual behavior and associated characteristics among low-income urban males in Peru. *PLoS ONE* 2007; **2 (8) (no pagination)**(e778).
237. Caceres CF, Konda KA, Salazar X, et al. New populations at high risk of HIV/STIs in low-income, urban coastal Peru. *AIDS Behav* 2008; **12**(4): 544-51.
238. Lama JR, Lucchetti A, Suarez L, et al. Association of herpes simplex virus type 2 infection and syphilis with human immunodeficiency virus infection among men who have sex with men in Peru. *Journal of Infectious Diseases* 2006; **194**(10): 1459-66.
239. Clark JL, Konda KA, Munayco CV, et al. Prevalence of HIV, herpes simplex virus-2, and syphilis in male sex partners of pregnant women in Peru. *BMC Public Health* 2008; **8 (no pagination)**(65).
240. Park H, Konda KA, Roberts CP, et al. Risk factors associated with incident syphilis in a cohort of high-risk men in peru. *PLoS ONE* 2016; **11 (9) (no pagination)**(e0162156).
241. Snowden JM, Konda KA, Leon SR, et al. Recent syphilis infection prevalence and risk factors among male low-income populations in coastal Peruvian cities. *Sexually Transmitted Diseases* 2010; **37**(2): 75-80.
242. Blas MM, Alva IE, Cabello R, et al. Internet as a tool to access high-risk men who have sex with men from a resource-constrained setting: A study from Peru. *Sexually Transmitted Infections* 2007; **83**(7): 567-70.
243. Perez-Brumer AG, Konda KA, Salvatierra HJ, et al. Prevalence of HIV, STIs, and Risk Behaviors in a Cross-Sectional Community- and Clinic-Based Sample of Men Who Have Sex with Men (MSM) in Lima, Peru. *PLoS ONE* 2013; **8 (4) (no pagination)**(e59072).
244. Silva-Santisteban A, Raymond HF, Salazar X, et al. Understanding the HIV/AIDS epidemic in transgender women of Lima, Peru: results from a sero-epidemiologic study using respondent driven sampling. *AIDS and behavior* 2012; **16**(4): 872-81.
245. Pizzicato LN, Vagenas P, Gonzales P, et al. Active syphilis and its association with HIV and sexual risk behaviours in a multicity sample of men who have sex with men and transgender women in Peru. *Sexual Health* 2017; **14**(4): 304-12.
246. Galea JT, Kinsler JJ, Galan DB, et al. Factors associated with visible anogenital warts among HIV-uninfected Peruvian men who have sex with men and transwomen: A cross-sectional study. *Sexually Transmitted Diseases* 2015; **42**(4): 202-7.
247. Blair C, Passaro RC, Segura ER, et al. Sexual network characteristics of men who have sex with men with syphilis and/or gonorrhoea/chlamydia in Lima, Peru: Network patterns as roadmaps for STI prevention interventions. *Sexually Transmitted Infections* 2019.
248. Allan-Blitz LT, Herrera MC, Calvo GM, et al. Venue-Based HIV-Testing: An Effective Screening Strategy for High-Risk Populations in Lima, Peru. *AIDS and behavior* 2019; **23**(4): 813-9.
249. Results from the HIV biological and behavioural surveillance in the Republic of Armenia 2010-2011. Armenia: Ministry of Health, Republic of Armenia, 2011.
250. Results from the HIV biological and behavioural surveillance in the Republic of Armenia 2012. Yerevan, Armenia: Ministry of Health, Republic of Armenia, 2013.
251. Results from the HIV biological and behavioural surveillance in the Republic of Armenia 2014. Yerevan, Armenia: Ministry of Health, Republic of Armenia, 2015.
252. Integrated biological-behavioral surveillance survey among people who inject drugs, female sex workers and men who have sex with men in Yerevan, Gyumri and Vanadzor, Armenia-2016. Armenia: Ministry of Health, Republic of Armenia, 2016.
253. Integrated biological-behavioural surveillance survey among people who inject drugs, female sex workers, men who have sex with men and transgender persons 2018. Yerevan, Armenia: Ministry of Health, Republic of Armenia, 2018.
254. Prevalence of HIV, hepatitis and syphilis, and behavioural risk factors among most-at-risk groups in the Republic of Azerbaijan. Baku, Azerbaijan: Ministry of Health of the Republic of Azerbaijan, 2008.

255. Report on the integrated bio-behavioural surveillance surveys among key populations in Azerbaijan, 2015. Azerbaijan: Ministry of Health of the Republic of Azerbaijan, 2015.
256. Prevention of HIV/AIDS and sexually transmitted infections among commercial sex workers, men who have sex with men and their clients. Georgia: Tanadgoma, Scientific Research Institute of Dermatology and Venerology, AIDS and Clinical Immunology Research Center, 2005.
257. Characteristics, high-risk behaviors and knowledge of STI/HIV/AIDS, and prevalence of HIV, syphilis and hepatitis among MSM in Tbilisi, Georgia: 2007. Georgia: Save the Children, Tanadgoma, AIDS and Clinical Immunology Research Center, 2008.
258. Bio-behavioral surveillance survey among men who have sex with men in Tbilisi, Georgia (2010). Georgia: Curatio International Foundation, Tanadgoma, 2010.
259. HIV risk and prevention behaviour among men who have sex with men in Tbilisi, Georgia, bio-behavioural surveillance survey in 2012. Georgia: Curatio International Foundation, Tanadgoma, 2013.
260. HIV risk and prevention behavior among men who have sex with men in Tbilisi and Batumi, Georgia, bio-behavioural surveillance survey in 2015. Georgia: Curatio International Foundation, Tanadgoma, 2015.
261. HIV risk and prevention behavior among men who have sex with men in Tbilisi and Batumi and Kutaisi, Georgia, bio-behavioural surveillance survey in 2018. Georgia: Curatio International Foundation, Tanadgoma, 2018.
262. Johnston LG, Alami K, Rhilani MHE, et al. HIV, syphilis and sexual risk behaviours among men who have sex with men in Agadir and Marrakesh, Morocco. *Sexually Transmitted Infections* 2013; **89**(SUPPL. 3): iii45-iii8.
263. Hakim AJ, Coy K, Badman SG, et al. One size does not fit all: HIV prevalence and correlates of risk for men who have sex with men, transgender women in multiple cities in Papua New Guinea. *BMC Public Health* 2019; **19**(1): 623.
264. An integrated biological and behavioral surveillance survey (IBBSS) among female sex workers and men who have sex with men in Solomon Islands. Solomon Islands: HIV/STI Division of the Ministry of Health and Medical Services, 2018.
265. Second Generation Surveillance Survey of MSM in Tonga 2008. Tonga: The Tonga Ministry of Health, 2008.
266. Veronese V, van Gemert C, Bulu S, et al. Sexually transmitted infections among transgender people and men who have sex with men in Port Vila, Vanuatu. *Western Pac Surveill Response J* 2015; **6**(1): 55-9.
267. Kendall C, Kerr LRFS, Mota RMS, et al. Population size, HIV, and behavior among MSM in Luanda, Angola: Challenges and findings in the first ever HIV and syphilis biological and behavioral survey. *Journal of Acquired Immune Deficiency Syndromes* 2014; **66**(5): 544-51.
268. 2012 Mapping, size estimation & behavioral and biological surveillance survey (BBSS) of HIV/STI among select high-risk sub-populations in Botswana. Botswana: Ministry of Health, 2013.
269. Goodman SH, Grosso AL, Ketende SC, et al. Examining the Correlates of Sexually Transmitted Infection Testing among Men Who Have Sex with Men in Ouagadougou and Bobo-Dioulasso, Burkina Faso. *Sexually Transmitted Diseases* 2016; **43**(5): 302-9.
270. Park JN, Papworth E, Kassegne S, et al. HIV prevalence and factors associated with HIV infection among men who have sex with men in Cameroon. *Journal of the International AIDS Society* 2013; **16 Suppl 3**: 18752.
271. [Etude biologique et comportementale des IST, du VIH et du sida chez les Hommes ayant des rapports Sexuels avec des Hommes des villes d'Abidjan, Agboville, Bouaké, Gagnoa, et Yamoussoukro].[French]. Cote d'Ivoire: Ministry of Health and Public Hygiene, 2016.
272. Mason K, Ketende S, Peitzmeier S, et al. A cross-sectional analysis of population demographics, HIV knowledge and risk behaviors, and prevalence and associations of HIV among men who have sex with men in the Gambia. *AIDS Res Hum Retroviruses* 2013; **29**(12): 1547-52.
273. Sanders EJ, Okuku HS, Smith AD, et al. High HIV-1 incidence, correlates of HIV-1 acquisition, and high viral loads following seroconversion among MSM. *Aids* 2013; **27**(3): 437-46.
274. Muraguri N, Tun W, Okal J, et al. HIV and STI prevalence and risk factors among male sex workers and other men who have sex with men in nairobi, kenya. *Journal of Acquired Immune Deficiency Syndromes* 2015; **68**(1): 91-6.
275. Integrated bio-behavioural surveillance survey (IBBSS2) report for key populations in Lesotho. Lesotho: Lesotho Ministry of Health, 2019.
276. Integrated bio-behavioural surveillance survey (IBBSS) among MARPs in Liberia. Liberia: Ministry of Health and Social Welfare, 2013.
277. Wirtz AL, Jumbe V, Trapence G, et al. HIV among men who have sex with men in Malawi: elucidating HIV prevalence and correlates of infection to inform HIV prevention. *Journal of the International AIDS Society* 2013; **16 Suppl 3**: 18742.
278. Wirtz AL, Trapence G, Kamba D, et al. Geographical disparities in HIV prevalence and care among men who have sex with men in Malawi: results from a multisite cross-sectional survey. *The Lancet HIV* 2017; **4**(6): e260-e9.
279. Wirtz AL, Trapence G, Gama V, et al. HIV prevalence and sociobehavioral characteristics among men who have sex with men across seven sites in Malawi. Malawi: Johns Hopkins University and the Center for Development of People, 2014.
280. Integrated behavioral and biological surveillance survey among men who have sex with men. Mauritius: AIDS Unit, Ministry of Health and Quality of Life, 2010.
281. Integrated biological-behavioral surveillance survey and population size estimation among transgender persons in Mauritius-2017. Mauritius: Prevention information et Lutte contre le sida (PILS), 2017.

282. Merrigan M, Azeez A, Afolabi B, et al. HIV prevalence and risk behaviours among men having sex with men in Nigeria. *Sexually Transmitted Infections* 2011; **87**(1): 65-70.
283. Wade AS, Kane CT, Niang Diallo PA, et al. HIV infection and sexually transmitted infections among men who have sex with men in Senegal. *Aids* 2005; **19**(18): 2133-40.
284. Wade AS, Larmarange J, Diop AK, et al. Reduction in risk-taking behaviors among MSM in Senegal between 2004 and 2007 and prevalence of HIV and other STIs. ELIHoS Project, ANRS 12139. *AIDS Care - Psychological and Socio-Medical Aspects of AIDS/HIV* 2010; **22**(4): 409-14.
285. Johnston LG, Holman A, Dahoma M, et al. HIV risk and the overlap of injecting drug use and high-risk sexual behaviours among men who have sex with men in Zanzibar (Unguja), Tanzania. *International Journal of Drug Policy* 2010; **21**(6): 485-92.
286. Ross MW, Nyoni J, Ahaneku HO, Mbwambo J, McClelland RS, McCurdy SA. High HIV seroprevalence, rectal STIs and riskxy sexual behaviour in men who have sex with men in Dar es Salaam and Tanga, Tanzania. *BMJ Open* 2014; **4 (8) (no pagination)**(e006175).
287. Mmbaga EJ, Moen K, Leyna GH, Mpembeni R, Leshabari MT. HIV Prevalence and Associated Risk Factors Among Men Who Have Sex With Men in Dar es Salaam, Tanzania. *Journal of acquired immune deficiency syndromes (1999)* 2018; **77**(3): 243-9.
288. Ruisenor-Escudero H, Lyons C, Ketende S, et al. Consistent Condom Use Among Men Who Have Sex With Men in Lome and Kara, Togo. *AIDS Research & Human Retroviruses* 2019: 03.
289. Ekouevi DK, Bitty-Anderson AM, Gbeasor-Komlanvi FA, et al. Low prevalence of syphilis infection among key populations in Togo in 2017: a national cross-sectional survey. *Archives of Public Health*; **77**: 39.
290. Kim EJ, Hladik W, Barker J, et al. Sexually transmitted infections associated with alcohol use and HIV infection among men who have sex with men in Kampala, Uganda. *Sexually Transmitted Infections* 2016; **92**(3): 240-5.
291. Kelsey JL, Whittemore AS, Evans AS, Thompson WD. Methods in Observational Epidemiology. 2nd ed. New York, NY: Oxford University Press; 1996.
